# Osmoregulation in the Halophilic Bacterium *Halomonas elongata*: A Case Study for Integrative Systems Biology

**DOI:** 10.1371/journal.pone.0168818

**Published:** 2017-01-12

**Authors:** Viktoria Kindzierski, Silvia Raschke, Nicole Knabe, Frank Siedler, Beatrix Scheffer, Katharina Pflüger-Grau, Friedhelm Pfeiffer, Dieter Oesterhelt, Alberto Marin-Sanguino, Hans-Jörg Kunte

**Affiliations:** 1 Faculty of Mechanical Engineering, Specialty Division for Systems Biotechnology, Technische Universität München, München, Germany; 2 Federal Institute for Materials Research and Testing (BAM), Division 4.1 Biodeterioration and Reference Organisms, Berlin, Germany; 3 Department of Membrane Biochemistry, Max Planck Institute of Biochemistry, Martinsried, Germany; Universidad Autonoma Metropolitana, MEXICO

## Abstract

Halophilic bacteria use a variety of osmoregulatory methods, such as the accumulation of one or more compatible solutes. The wide diversity of compounds that can act as compatible solute complicates the task of understanding the different strategies that halophilic bacteria use to cope with salt. This is specially challenging when attempting to go beyond the pathway that produces a certain compatible solute towards an understanding of how the metabolic network as a whole addresses the problem. Metabolic reconstruction based on genomic data together with *Flux Balance Analysis* (FBA) is a promising tool to gain insight into this problem. However, as more of these reconstructions become available, it becomes clear that processes predicted by genome annotation may not reflect the processes that are active *in vivo*. As a case in point, *E*. *coli* is unable to grow aerobically on citrate in spite of having all the necessary genes to do it. It has also been shown that the realization of this genetic potential into an actual capability to metabolize citrate is an extremely unlikely event under normal evolutionary conditions. Moreover, many marine bacteria seem to have the same pathways to metabolize glucose but each species uses a different one. In this work, a metabolic network inferred from genomic annotation of the halophilic bacterium *Halomonas elongata* and proteomic profiling experiments are used as a starting point to motivate targeted experiments in order to find out some of the defining features of the osmoregulatory strategies of this bacterium. This new information is then used to refine the network in order to describe the actual capabilities of *H*. *elongata*, rather than its genetic potential.

## Introduction

A broad range of microorganisms thrives in environments with varying and high concentration of sodium chloride [1, 2]. An important osmoregulatory mechanism that allows microorganisms to cope with high salinities is the ability to amass highly water-soluble organic compounds, which do not disturb the cell’s metabolism even at high cytoplasmic concentrations and are therefore named compatible solutes [3]. The halophilic γ-proteobacterium *Halomonas elongata* DSM 2581^T^ synthesizes the aspartate derivative ectoine [4–7] as its main compatible solute [8]. By accumulating ectoine, *H*. *elongata* achieves a broad salt tolerance and can even survive in salt saturated brines (>5 M, 30% NaCl) [9]. Following a sudden increase in salt concentration, *H*. *elongata* increases the cytoplasmic K^+^ concentration along with the organic counter ion glutamate as a first response. Contrary to *Escherichia coli* and other mostly non-halophilic bacteria [10], the K^+^-glutamate level stays elevated in *H*. *elongata* for an extended time of at least two hours [11, 12]. Potassium is accumulated in the cytoplasm by transport via the two Trk-type transport systems TrkH and TrkI [13]. With increasing K^+^-glutamate levels, ectoine synthesis is switched on. The compatible solute ectoine is critical for the biology of *H*. *elongata* as shown by the mutation of genes involved in its biosynthesis. The genes *ectABC* encode the enzymes for conversion of the intermediate DABA (diaminobutyric acid) to ectoine. Cells with a deleted *ectA* gene (L-2,4-diaminobutyrate Nγ-acetyltransferase) failed to grow in medium containing more than 0.7 M NaCl (4%) [14, 15], a salt concentration that is within the optimal growth range. This result emphasizes the importance of ectoine for adaptation to high salinity in *H*. *elongata*. As shown by Göller [16], synthesis of ectoine is regulated at the level of transcription and at the level of enzyme activity. The *ectABC* genes are under the control of an osmoregulated σ^38^ dependent promoter upstream of *ectA*, a σ^70^ dependent promoter, and a σ^54^ dependent promoter in front of *ectC* [17]. This set of promoters is consistent with the observed transcription regulation data.

*Halomonas elongata* does not rely only on *de novo* synthesis of ectoine for osmoadaptation, but can also take up compatible solutes from the medium such as glycine-betaine via the two BCC-family transporters BetG and BetH [12]. However, only one transport system, the TRAP-transporter TeaABC, catalyzes specifically ectoine uptake in *H*. *elongata* [15]. TeaABC is not only required for the osmoregulatory accumulation of external ectoine, but also counterbalances an unknown system responsible for excreting endogenous ectoine to the medium. There is evidence that TeaABC might be linked to the regulation of ectoine synthesis [15, 18]. A model explaining the involvement of TeaABC with ectoine synthesis was developed [19], which helped to design an ectoine production strain of *H*. *elongata* with higher productivity for ectoine than the wild-type strain [18].

Ectoine is synthesized by a large number of aerobic, heterotrophic bacteria [20, 21] and is also a dominant compatible solute in halophilic methanotrophs and methylotrophs [22–24] However, despite the ability to synthesize ectoine, quite a few of these bacteria such as *Pseudomonas stutzeri* and many *Vibrio* species display reduced salt tolerance compared to *H*. *elongata* and are restricted to environments containing less than 1.7 M NaCl (10%) [25]. Apparently, different osmoregulatory mechanisms must exist in *H*. *elongata* and other halophilic bacteria that allow them to cope with salt concentrations well above 1.7 M NaCl (10%). In a recent paper, Pastor and coworkers analyzed the metabolism of the halophilic bacterium *Chromohalobacter salexigens* [26], which has a similar salt tolerance to *H*. *elongata* and synthesizes ectoine as its major compatible solute. By applying isotope label tracing they found out that the metabolism of *C*. *salexigens* is adapted to support high metabolic fluxes towards synthesis of ectoine. While this makes the metabolism well suited for survival in high saline environments, it is less suited for growth at low salinity.

To gain more information about the metabolic mechanisms that allow *H*. *elongata* to thrive at salinities above 1.7 M NaCl (10%), we identified target proteins by a preliminary quantitative profiling analysis of the membrane and soluble proteome at optimal (1 M NaCl), 6%), low (0.1 M, 0.6%), and high salinities (2 M, 12%). Target proteins were further analyzed by measuring the enzyme activity at different salinities and/or monitoring the expression of the corresponding genes by RT-qPCR. The data from these experiments were used to build a stoichiometric model of osmoadaptation in *H*. *elongata*. Our results indicate that significant osmoregulatory events go far beyond the ectoine synthesis pathway. There is a systemic response that requires coordinated action of different subsystems such as the PEP-pyruvate-oxaloacetate node and sodium transport.

## Materials and Methods

### Bacterial strains and growth conditions

*Halomonas elongata* DSM 2581^T^ was grown on minimal medium MM63 [27] at 30°C with 0.85 M NaCl or on complex medium LB containing 0.85 M NaCl (5% w/v). For proteomic analysis (see below) cells of *H*. *elongat*a were cultivated in a modified liquid glucose mineral-salt medium (GM) with 0.1 M (0.6%), 1.0 M (6%), and 2.0 M NaCl (12%), respectively [28]. For RT-qPCR analysis and characterization of metabolic pathways by enzyme assays, *H*. *elongata* cells were cultured under aerobic conditions in GM medium with 0.1 M (0.6%), 1.0 M (6%), and 2.0 M NaCl (12%), respectively, and harvested in the exponential growth phase. Bacterial cell mass was determined photometrically at 600 nm against medium as a reference (OD_600_).

### RNA isolation and quantitative RT-PCR (RT-qPCR)

Total RNA was isolated from exponentially growing cells using a modified hot phenol method [29] and further purified using the NucleoSpin RNA-isolation kit (Macherey & Nagel) according to the manufacturer's instructions. cDNA for quantitative RT-PCR analysis was synthesized by random priming using the iScript kit (Bio-Rad) according to the manufacturer’s instructions. After synthesis, cDNA was diluted 1:15 with DEPC-water and cDNA was quantified with a NanoDrop photometer (Thermo Scientific). cDNA (10 ng, approximately 2 μl) was added to iTaq Universal SYBR Green Supermix (total volume 10 μl), which contained forward primer and reverse primer at a concentration of 25 pmol each. The name of the target genes and the DNA sequences of the 23 primer pairs used to study the expression of the genes are provided in Table A in the S1 File (Supplementary Material). RT-qPCR amplification and quantification (2^ΔCt^ method) was carried out on a Bio-Rad CFX96 PCR-cycler applying the following cycle: 95°C for 3 min; 39 cycles of 95°C for 5 s to 15 s and 60°C for 30 s. For each gene, RNA isolation, cDNA synthesis and qPCR analysis was repeated at least twice (three biological replicates). For each biological replicate, cDNA synthesis and qPCR analysis (three runs in parallel) was repeated at least one time (two technical replicates). Statistical analysis of the obtained data was carried out using the Origin software package (OriginLaB).

### Enzyme assays

The halophilic bacterium *Halomonas elongata* DSM 2581^T^ [9] was used in all enzyme activity measurements in this study. Batch cultures were first grown in 2 ml GM medium. In a second step, 100 ml GM medium (1 M NaCl) in 500 ml flasks were inoculated with an OD_600nm_ of 0.015 and left to grow over night. Subsequently, the cultures were respectively adapted to varying salt concentrations (0.1; 1; 2 M) during three over night cultures with inoculation ODs of below 0.015 being used. The following main cultures were left to grow in their adapted salt concentration and harvested for the enzyme activity measurements the following day, during late exponential phase, by pelleting for 20 min at 25°C with 3,200 x g.

The enzyme assays were adapted for *H*. *elongata*, with enzyme activity being determined by monitoring the oxidation of the coenzymes NADH and NADPH (340 nm) or the formation of fumarate in the case of fumarase (240 nm), in a 96-well microplate reader (Infinite M200 PRO Series, Tecan) for 10 min at 30°C. Enzyme activity was defined as the micromoles of substrate consumed or product formed per minute and mg of total protein (Units mg^-1^).

In each case, cell pellets were put on ice and resuspended in 65 mM ice cold phosphate buffer (pH 7.5) containing 1 mM EDTA. Cells were sonicated on ice with a 3 mm diameter probe using a Digital Sonifier S450D (Branson) and centrifuged subsequently for 20 min at 4°C with 15,000 x g to remove cell debris. Afterwards the supernatant was filtered (0.4 μm pore size) to remove any intact cells and the resulting cell free extract was used for subsequent activity measurements. Protein concentration in cell-free extracts was determined by a BCA Protein Assay Kit (Pierce, Thermo Scientific).

#### Alanine dehydrogenase (*ald)*

A modified version of the assay described by Hutter & Dick [30] was used to measure the enzyme activity of the alanine dehydrogenase. The co-substrates NADH and NADPH were measured each, to define the co-substrate specificity of the enzyme, while the substrate pyruvate alone was used as a negative control. The assay mixture consisted of 50 mM TRIS-HCl buffer (pH 8.5) containing 1 mM EDTA, 100 mM NH_4_Cl, 15 mM pyruvate and either 0.5 mM NADH or 2 mM NADPH.

#### Fumarase (*fumC*)

The enzyme activity of fumarase was determined by a modified protocol of Van der Werf and colleagues [31] where the formation of fumarate was tracked at 240 nm. The reaction mixture consisted of 90 mM TRIS-HCl buffer (pH 8.0) and the substrate 50 mM malat (pH 7.0), the absence of which was also used as a negative control.

#### Glutamate dehydrogenase (*gdh*)

The activity of the glutamate dehydrogenase was measured according to Tesch and colleagues [31] with modifications. To determine the co-substrate specificity of the dehydrogenase, NADH and NADPH were measured each, while the absence of the substrate α-ketoglutarate was used as a negative control. The master mix consisted of 50 mM TRIS-HCl buffer (pH 8.5) containing 1 mM EDTA, 100 mM NH_4_Cl, 15 mM α-ketoglutarate and either 0.5 mM NADH or 2 mM NADPH.

#### Glutamate synthase (*gltDB*)

The enzyme activity of the glutamate synthase was determined by following the oxidation of NADPH in an assay originally described by Singh & Srivastava [32]. The enzyme catalyzes a reaction with the substrates L-glutamine and α-ketoglutarate, both of which were left out in negative controls. The assay mixture contained 25 mM phosphate buffer (pH 7.5) with 1 mM EDTA, 2.7 mM L-glutamine, 0.67 mM α-ketoglutarate, 3.3 mM KCl and 0.4 mM NADPH.

#### Malic enzyme (*Mae*)

The activity of the malic enzyme was measured according to Van der Werf and co-workers [33] with slight modifications. The reaction mixture contained 0.1 M Tris-HCl (pH 7.5), 2.5 mM MgCl_2_, 2 mM NADP^+^ and the substrate 10 mM malate (pH 7), the absence of which was used as a negative control.

#### Phosphofructokinase (*Pfk*)

To measure the co-substrate specificity of the phosphofructokinase, we used an assay according to Peng & Mansour [34] with modifications. The enzyme activity was determined in a coupled enzyme assay, with the standard assay master mix containing 50 mM Tris buffer (pH 8.5) with 1 mM EDTA and 1 mM DTT, 5 mM MgCl_2_, 0.2 mg ml^-1^ NADH, 1.5 U aldolase, 15 U triosephosphate isomerase, 5.1 U glycerolphosphat dehydrogenase (all from Sigma Aldrich, Germany), 1 mM Fru-6-P, 1 mM ATP and 1 mM PPi. The assays were run with the following controls: without Fru-6-P and without co-substrate ATP or PPi, respectively.

### Metabolic labeling for quantitative proteomic analysis

*Halomonas elongata* was cultivated on MM63 agar plates and transferred onto LB agar. After two days at 30°C the cells were transferred into GM liquid medium (20 ml) with 0.1 M, 1.0 M, and 2.0 M NaCl, respectively. The medium with 1.0 M NaCl (6%) contained ^15^NH_4_Cl as the sole nitrogen source and the media with 0.1 M NaCl and 2 M NaCl (12%) contained ^14^NH_4_Cl as the sole nitrogen source. After overnight incubation the cells were transferred into 15 ml GM medium with the corresponding salt concentration of 0.1 M (0.6%), 1 M (6%), and 2 M NaCl (12%), respectively. After overnight growth, cells were transferred into fresh 25 ml GM medium and the OD_600_ was adjusted to 0.5. The cells were further grown at 30°C until they reached an OD_600_ of 1.0 and harvested by centrifugation (10,000 x g) at 4°C. The cell pellet was resuspended in 500 μl TRIS buffer (0.1 M, 1 M, 2 M NaCl, pH 7.4) and spun down again and resuspended in 1 ml TRIS buffer with the corresponding salt concentration.

After washing, the OD_600_ for all three cell-samples was determined and the labeled cells (1.0 M NaCl, 6%) were mixed with the unlabeled cells from the cultures with 0.1 M NaCl (0.6%) and 2 M NaCl (12%), respectively, in a ratio of 1:1. Immediately afterwards, cells were lysed by French Pressing in 25 ml cell lysis buffer (0.1 mM TRIS, 0.5 M NaCl, which contained 187 mg MgCl_2_, 136 mg MnCl_2_, 250 μg DNase, 250 μg RNase (per 25 ml), one protease inhibitor tablet (Roche, Mannheim, Germany), pH 7.4). Cell debris was removed by centrifugation (10,000 x g) at 4°C and the supernatant was centrifuged at high speed (100,000 x g) for 1 h at 4°C in order to separate the membrane and cytoplasmic fraction of the cells.

After ultracentrifugation, 250 μl supernatant containing the cytoplasmic fraction was removed from the membrane pellet and treated with 5 volumes ice-cold acetone in order to precipitate the proteins. After 24 h incubation at -70°C, the proteins were pelleted by centrifugation (15,000 x g) for 30 min at 4°C. After vaporizing the acetone, the sample was resuspended in 300 μl H_2_O by sonication. Methanol (25 μl) was added and the sample was frozen in liquid nitrogen for 2 min and dried by lyophilisation.

The membrane pellet was resuspended in 2 ml TRIS buffer (0.1 M, pH 7.4) containing 0.5 M NaCl and filled up to 20 ml with TRIS-TWEEN buffer (0.1 M TRIS, pH 7.4, 0.5 M NaCl, 5% TWEEN). In order to remove remaining cytoplasmic proteins, the membrane was washed by stirring for 20 min at RT. The purified membrane was separated from the cytoplasmic proteins by high-speed centrifugation (100,000 x g, 1 h, 4°C). Afterwards the membrane pellet was resuspended in 10 ml TRIS buffer (0.1 M, pH 7.4, 0.5 M NaCl), centrifuged (100,000 x g, 4°C) for 1 h, again resuspended in TRIS buffer and centrifuged (100,000 x g, 4°C) for 30 min. Finally, the purified membrane was resuspended in 100 μl H_2_O and delipidated with chloroform/methanol according to Wessel and Flugge [35].

### 1-D-gel electrophoresis

Proteins were resuspended in 2x SDS-PAGE sample buffer (Rockland Immunochemicals) by vortexing and ultrasound treatment. Proteins were separated on NuPAGE Novex Bis-Tris Mini gels (Invitrogen) by one-dimensional SDS-gel electrophoresis at 200 V for 45 min. Gels were stained with Fermentas PageBlueProtein staining solution according to the manufacturer’s instruction. Gel lanes were cut into 16 slices, which were then cut into small pieces of approximately 1 mm^3^ for in-gel proteolytic digestion. Gel pieces were destained by incubation in 100 μl acetonitrile (ACN; 50%, v/v) for 15 min at RT. After removal of the supernatant 100 μl ammonium bicarbonate (ABC; 50 mM) was added followed by incubation for 15 min. The destaining procedure and ABC incubation was repeated twice. Proteins were reduced in 50 mM ABC solution with 100 mM dithiothreitol (100 μl) for 45 min at 56°C. Proteins were alkylated by incubation in 100 μl ABC solution (50 mM) with 55 mM iodacetamide in the dark for 30 min. After alkylation, gel pieces were washed by incubation with 50% ACN for 15 min. ACN was completely removed and gel pieces were washed with 50 mM ABC for another 15 min. After complete removal of ABC, the ACN/ABC washing steps were repeated twice. For in-gel digestion 26 μl trypsin solution (20 μg trypsin dissolved in 20 μl storage buffer and diluted with 5.2 ml 50 mM ABC) was added to the gel pieces, which were incubated over night at 37°C and 300 rpm (Eppendorf Thermomixer comfort). To elute the peptides, the gel pieces were shortly spun down and the liquid supernatant was transferred into a new tube. Remaining peptides were eluted from the gel by subsequently adding and removing (after 20 min incubation) 50 μl extraction solution. Extraction solutions were in consecutive order: H_2_O, ACN (50% v/v), and 0.1% TFA in ACN (50% v/v). All supernatants from one slice were combined and the samples were desalted using self-made extraction tips as described by Rappsilber and co-workers [36].

### Nano-LC-MS/MS analysis

The LC-MS/MS analysis was carried out according to Dulla and co-workers [37]. Briefly, peptides were solubilized with acetic acid (0.5%, v/v) and the nano-LC-MS/MS analysis was performed on a linear trap quadropole (LTQ) Orbitrap mass spectrometer (Thermo Fisher Scientific, Waltham, MA) equipped with a nanoACQUITY UPLC (Waters, Milford, MA). The peptide samples were separated via an analytical column (3 μm, ReproSil-Pur C_18_ AQ, Dr. Maisch GmbH) with a gradient from 100% buffer A (0.2% acetic acid (v/v) in water) to 100% buffer B (0.2% acetic acid (v/v) in acetonitrile) in a time frame of 180 min.

### Data analysis

All MS/MS spectra obtained were searched against the *H*. *elongata* protein sequence database, which was exported from the HaloLex database (www.halolex.mpg.de) [38, 39], using a locally installed MASCOT server (version 2.0) [40]. For the database search, carbamidomethylation was set as a required cysteine modification, whereas oxidation of methionine was considered as a variable modification. As a further criterion the ^15^N-labeling strategy was chosen. Furthermore, a missed cleavage site in trypsin digestion was enabled, the decoy database option was activated and the threshold was set to 5 ppm for MS and 0.5 Da for MS/MS. The obtained dataset was quantified using the Mascot Distiller software with the following settings: Using the ^15^N-labeling strategy the minimum ion score was set to 15. The function Bold Red was activated in order to allow for only non-ambiguous protein and peptide hits. The occurrence of both, light and heavy peptides (^14^N and ^15^N) was set as a prerequisite condition. For quantification the correlation threshold was set to 0.9 and the standard error threshold to 0.16. The peak-area of the light and heavy species of a peptide was considered for quantification. The ratio of the peak-area for ^14^N/^15^N was computed and normalized to result in 1.0 as the median value. Ratios below 1.0 were transformed by computation of the inverse value, being represented as a negative number. For the membrane proteome, datasets were generated from three independent experiments. Only proteins that were identified in at least two of the three datasets and had a regulation factor of 1.8 or higher were considered for final quantification. The cytoplasmic proteome was measured once and up-regulated proteins were further investigated by mutagenesis and quantitative RT-PCR in order to verify the proteome data. Outliers were identified by a modified Box-Plot analysis and removed. Proteins showing a regulation factor of >5 were manually inspected and adjusted if required.

### Flux Balance Analysis (FBA) and metabolic modeling

A stoichiometric model was formulated [41], which includes 85 reactions accounting for the function of 129 loci identified during genome annotation and a number of additional reactions. In addition to the ectoine synthesis pathway, the model includes other relevant pathways such as: Embden-Meyerhoff-Parnas, Pentose-phosphate cycle, Entner-Doudoroff (cytoplasmic and periplasmic variants), tricarboxylic acid cycle (TCA cycle), anaplerotic reactions, glyoxylate shunt and nitrogen assimilation through glutamate and alanine. It also includes lumped reactions to reflect the impact of different physiological states: Overall ATP consumption by the cell, overall NADPH consumption e.g. due to oxydative stress formation of PP_i_ by polymerization processes, proton leak through the membrane and uptake of compatible solute transporters. Proton sodium antiporters with the usual stoichiometries are also included. The latter reactions have not been linked to any locus because even though the transporters are identified (see section on sodium economy) the specific stoichiometry of each antiporter has not yet been established.

Optimal flux distributions for ectoine production were obtained by linear programming as commonly used for FBA. Optimization was carried out using python and the linear programming solvers GLPK (http://www.gnu.org/software/glpk/glpk.html) and Gurobi (http://www.gurobi.com).

## Results and Discussion

### General remarks

In the present study we employed a ^14^N/^15^N metabolic labeling method for LC-MS/MS analysis in order to identify *H*. *elongata* proteins with a potential role in adaptation to different salinities. Proteins of interest identified by this method were further characterized by measuring transcriptional regulation of the corresponding genes and by measuring changes in enzyme activity. Cultures of *H*. *elongata* were propagated in minimal medium containing 0.1 M (0.6%), 1 M (6%), and 2 M NaCl (12%) and harvested in the exponential growth phase for quantitative MS-analysis of the proteome, transcript quantification by RT-qPCR and quantification of enzyme activity. For measuring the protein content by Nano-LC-MS/MS, cells of the halophilic bacterium grown at an optimal salt concentration of 1 M NaCl (6%) were labeled with ^15^NH_4_Cl and mixed with unlabeled cells from low-salt (0.1 M NaCl, 0.6%) and high-salt (2 M NaCl, 12%), respectively.

According to genome annotation, *H*. *elongata* possesses 3473 proteins [17]. We were able to detect 1488 proteins, which represent 43% of the predicted proteome. In total, the protein content of 470 proteins was up or down regulated with increasing and decreasing salinity. We considered a protein as regulated and worthwhile for further investigation if the protein content was 1.8 times higher or lower at 0.1 M NaCl (0.6%) or 2 M NaCl (12%), respectively, compared to the protein level at 1 M NaCl (6%). From these 470 proteins, we chose 24 corresponding genes for transcript quantification by RT-qPCR. These genes encode proteins with function in glucose degradation (quinoprotein glucose dehydrogenase, phosphogluconate dehydratase, 2-dehydro-3-deoxy-6-phosphogalactonate aldolase of the ED-pathway), in anaplerosis and the TCA cycle (including malic enzyme, isocitrate lyase, fumarase), in nitrogen metabolism (including glutamate synthase, glutamate dehydrogenase), ectoine synthesis, ectoine degradation and ectoine transport. Furthermore, the activity of the enzymes glutamate synthase, glutamate dehydrogenase, alanine dehydrogenase, malic enzyme and fumarase at low, optimal and high salinity was determined. The activity of 6-phosphofructokinase was tested in the presence of ATP and PP_i_ to determine the source of phosphate for the reaction.

Genes and proteins, respectively, that are regulated according to the combined analyses by RT-qPCR, MS-analysis and enzyme assays have been grouped into functional categories and are listed in Tables B and C in the S2 File (Supplementary Material).

### Carbohydrate metabolism

**Fig 1:**
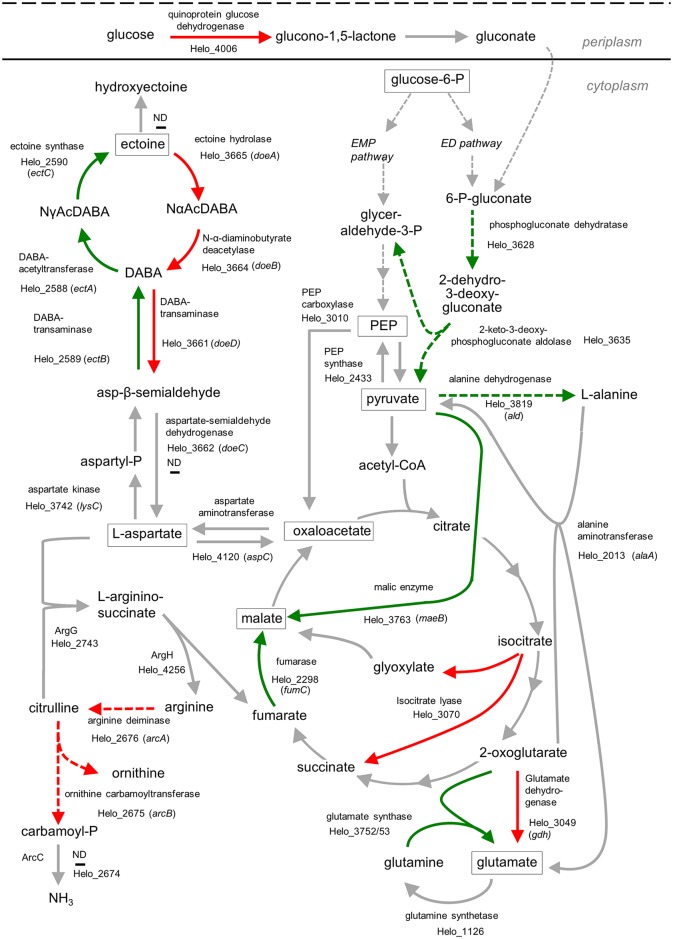
Metabolic pathways in *Halomonas elongata* that are regulated with increasing salt concentration (0.1 M, 1 M, and 2 M NaCl). Metabolic reactions shown in solid red (down regulated) and solid green (up regulated) lines are regulated by salinity according to mass spectrometry (MS) and RT-qPCR analysis. Dashed arrows show reactions that are regulated based on MS data only. In addition, the regulation of activity by salinity was determined for the enzymes phosphofructokinase, malic enzyme, glutamate synthase, glutamate dehydrogenase, alanine dehydrogenase and fumarase (for details see paragraphs Carbohydrate metabolism, Tricarboxylic acid cycle and Ammonia assimilation).) Depicted is an overview of glycolysis (EMPEmbden-Meyerhof-Parnas; ED = Entner-Doudoroff) and the TCA cycle with the affiliated pathways of ectoine synthesis and glutamate synthesis. Pathway for arginine degradation via ornithine/carbamoyl-P is shown as well. Gray arrows show pathways that remain unchanged or escaped detection by proteome profiling (ND). Proteins detected by quantitative MS-analysis were considered for further investigation (RT-qPCR of the corresponding genes, enzyme assay) if the protein content changed by the factor ≥1.8 with increasing salinity. The protein content of phosphogluconate dehydratase and 2-keto-3-deoxy-phosphogluconate aldolase (dashed green arrows) was up regulated (MS-analysis) but the transcription appeared unchanged (RT-qPCR). The protein content of alanine dehydrogenase appeared up regulated according to the MS data but the transcription and enzyme activity were unchanged by salt. However, the activity of this enzyme was much higher than any other enzyme included in this study. Quantitative MS analysis revealed down regulation of glutamate dehydrogenase (*gdh*) at 1 M NaCl by the factor 1.79 (threshold 1.8) but was still further analyzed by enzyme assay and RT-qPCR, which proved a significant decline in enzyme activity and transcription at high salinity. The exact regulation factors derived from the MS data and the qPCR results can be found in the Supplementary Material (Figures A to W in S1 File and Tables B and C in S2 File). DABA: L-2,4-diaminobutyrate; MS: mass spectrometry.

**Fig 2:**
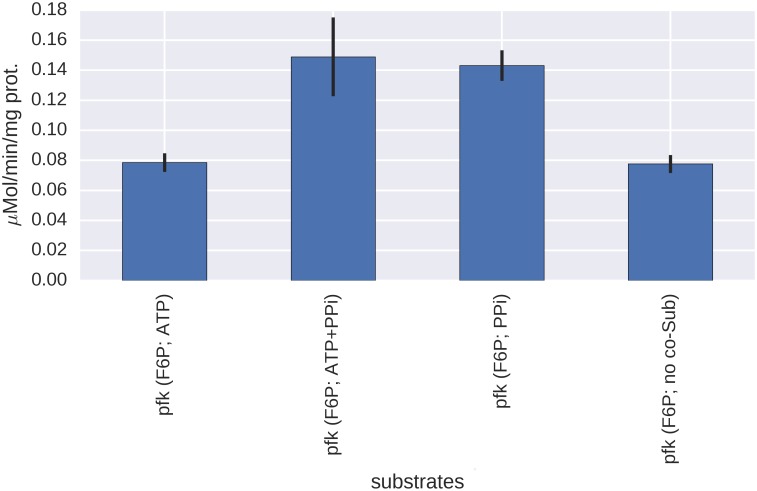
Phosphofructokinase (PFK) specificity. Activity of PFK in crude extract with fructose-6-phosphate and different co-substrates.

A recent detailed survey of a wide range of marine bacteria found that most of them had the required genes to metabolize glucose using three different pathways, namely the Entner-Doudoroff (ED), the Embden-Meyerhof-Parnas (EMP) and the pentose-phosphate pathway (PPP) [42]. ^13^C flux-profiling experiments showed that all of them catabolized glucose using either ED or EMP, sometimes complemented by PPP. All bacteria using EMP were shown to express an ATP-dependent 6-phosphofructokinase but no glucose-6-dehydrogenase while the opposite was found with those using ED. All Halomonadaceae included in the study used ED exclusively, and so does *C*. *salexigens*, the close relative of *H*. *elongata* [26]. The genome of *H*. *elongata* seems to follow the pattern, since it has the genes for all three pathways plus an additional non-phosphorylated ED, as it can be seen in Fig 1 depicting the upper glycolysis as annotated in the genome. It is noteworthy that no gene for fructose-1,6-bisphosphatase has been found in the genome of *H*. *elongata*, although it is reported that *H*. *elongata* is able to grow on lactate [9]. Another peculiarity is that the enzymes for both ED and EMP pathways were detected in the proteomic analysis unlike the cases studied by Klingner and co-workers [42]. However, only the expression of the ED-pathway is up regulated with increasing salinity (phosphogluconate dehydratase, 2-keto-3-deoxyphosphogluconate aldolase), while none of the enzymes participating in the EMP-pathway and the pentose-phosphate pathway are significantly regulated by salt. It should be pointed out that these findings are only based on the MS-analysis. It has been shown recently that the glucose catabolism in *C*. *salexigens* and other bacteria of the Halomonadaceae proceeds entirely via the ED-pathway [26, 42]. A possible explanation from these differences can be attributed to the specificity of the 6-phosphofructokinase present in *H*. *elongata*. All metabolic reconstructions of Halomonadaceae presented so far [43, 44] have assigned ATP specificity to the phosphofructokinase. However, the protein Helo_2186, which is annotated as 6-phosphofructokinase, shares only 30% similarity or less to experimentally characterized phosphofructokinases from bacteria such as *E*. *coli* and *B*. *subtilis*. The potential 6-phosphofructokinase from the closely related halophile *C*. *salexigens* is very similar to Helo_2186, and likewise, only remotely similar to experimentally characterized PFKs. Fig 2 shows the results of enzyme assays performed in crude extract. It can clearly be seen that the enzyme uses PP_i_ instead of ATP as phosphate donor. This can have deep implications since the reaction with pyrophosphate has a standard free energy of roughly one third of that with ATP (-4.4 kJ/mol vs -15.0 at pH 7.0 and ionic strength 0.1 according to the thermodynamic database Equilibrator at http://equilibrator.weizmann.ac.il/). The PP_i_ dependent 6-phosphofructokinase is reversible and a plausible substitute for the missing phosphatase and explains a functional gluconeogenesis in *H*. *elongata*.

### Tricarboxylic acid cycle

**Fig 3:**
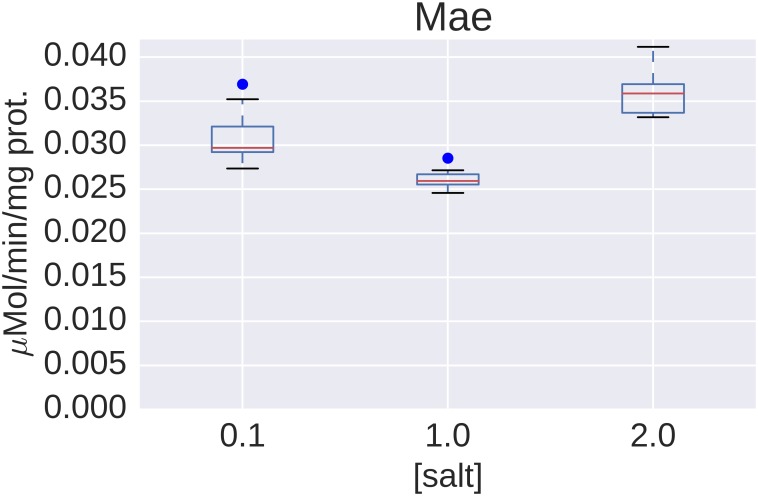
Activity of malic enzyme in crude extract of *H*. *elongata*. The box-plot shows data from a representative experiment in which three cultures were grown in each of the salt concentrations (0.1 M, 1 M and 2 M NaCl). Four crude extracts were prepared from each of the nine cultures, so each box summarizes twelve data points.

Among the anaplerotic reactions, the expression of the malic enzyme (MaeB, Helo_3763) seemed to be upregulated by salt in the proteome and at the level of transcription. The second putative NAD-dependent malic enzyme (MaeA, Helo_3817) could not be identified by MS analysis. All optimal flux distributions calculated using FBA to determine theoretical maximum yields of ectoine channeled the flux through malic enzyme: PEP is converted via pyruvate and malate to oxaloacetate, which can then be converted to ectoine via aspartate. Using this pair of reactions, the overall conversion of glucose to ectoine results in the generation of one ATP. Thus, the route via malic enzyme would be the most energy-efficient pathway. According to our previously reported thermodynamic calculations [17], the overall conversion is thermodynamically feasible. RT-qPCR showed a similar trend to that seen in the prospective proteome profiling but only a small, albeit statistically significant overexpression was detectable. This can be explained by the results of the enzyme assays (Fig 3), which show an extremely low activity of malic enzyme under all three conditions. The activities of the malic enzyme in *H*. *elongata* where consistently one order of magnitude below the values of *P*. *putida* reported in literature [45] and below the values measured by us. Our findings indicate that the malic enzyme plays no significant role as an anaplerotic reaction.

According to our metabolic modeling (see below), flux through the glyoxylate cycle at increased salinities cannot improve ectoine synthesis. The proteome analysis data indicate a down-regulation of the isocitrate lyase (Helo_3070) and the RT-qPCR data revealed a strong down-regulation of Helo_3070 transcription at salinities of 1 M (6%) and 2 M NaCl (12%) compared to 0.1 M NaCl (0.6%). Furthermore, experiments with labeled substrates have shown that the glyoxylate shunt plays no role in osmotic stress response in *H*. *elongata* [46].

The two remaining anaplerotic reactions catalyzed by oxaloacetate decarboxylase (Oad, Helo_3734–3736) and phosphoenolpyruvate carboxylase (Ppc, Helo_3010) can supply the required oxaloacetate to the TCA cycle through carboxylation of either phosphoenolpyruvate (PEP) or pyruvate. Carboxylating pyruvate through the Oad reaction enables the generation of one extra ATP molecule in comparison to the route via PEP-carboxylase. However, the Oad reaction leads to the influx of two sodium ions per carboxylation. The comparative metabolic costs of these two options will therefore depend on the active sodium transport systems.

### Sodium economy

In bacteria, sodium efflux is mediated by four principal types of ion transporters: proton-driven Na^+^/H^+^-antiporters [47], Na^+^-coupled respiratory enzymes (NADH:quinone oxidoreductases; NQR) [48], V-type ATPases [49], and biotin-containing decarboxylases [50]. We found at least 10 genes in the *H*. *elongata* genome encoding putative Na^+^ efflux systems that belong to these four groups of transporters.

**Fig 4:**
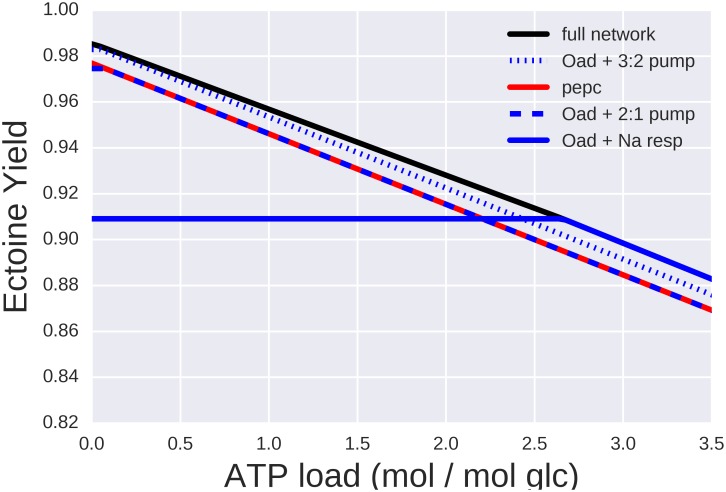
Maximal ectoine yield for different ATP loads (ATP demands external to the network) calculated using Flux Balance analysis (FBA). The black solid line shows the values when the full network is operational. Additional lines show yields when only the indicated subset of anaplerotic pathways and Na transporters are active (see text for details).

The presence of the anaplerotic reaction oxaloacetate decarboxylase, a biotin-containing decarboxylase that also functions as a sodium pump, ties the relative performance of anaplerotic alternatives to sodium transport. Transporting the two sodium ions [51] needed for a carboxylation of pyruvate has an “opportunity cost'' in terms of ATP, since the sodium ions must be extruded at the expense of protons. When the sodium translocation occurs through the NADH:quinone oxidoreductase (NQR) at the respiratory chain, each sodium ion is transported instead of a proton. Since the Oad reaction for the carboxylation of pyruvate leads to the influx of two Na^+^, the export of two Na^+^ via NQR costs 2 protons. This is equivalent to 1/2 ATP opportunity cost for every carboxylation, since the ATPase stoichiometry is four protons per ATP [52]. The sodium/proton antiporters have diverse stoichiomeries, with 2:3 and 1:2 being the most common, which would result in opportunity costs of 3/4 ATP and 1 ATP, respectively. To investigate the impact of these different costs within the context of the whole metabolic network, different combinations of active reactions were compared (Fig 4). When the full network is active, different combinations of anaplerotic and sodium transport strategies can result in maximum ectoine yield, which vary depending on the ATP load. For high ATP loads, the cost considerations stated above clearly make carboxylation through Oad and subsequent extrusion of the sodium ions through the respiratory chain the optimal choice. When the ATP load is low, the most efficient solution is the carboxylation through Oad and extrusion of sodium through the 2:3 antiporter. The least efficient solutions, although not by a large margin, are PEP carboxylase and the combined action of Oad and the 2:3 pump, which can be easily seen to be equivalent by inference from the opportunity costs. The reasons for NQR not always being the best solution instead of being the most efficient in terms of ATP costs results from the translocation of 2 H^+^ together with the 2 Na^+^, which results in the production of more ATP than needed.

Thus, the tandem of anaplerotic enzymes Oad and PEP carboxylase, in conjunction with the different sodium transport mechanisms, seems to be a very efficient method to obtain extra exergonism in the carboxylation by coupling it to the sodium gradient while remaining flexible under a variety of conditions.

### Potassium influx

In a first response to increasing NaCl concentrations in the surrounding, *H*. *elongata* accumulates K^+^ and the counter ion glutamate. Potassium is taken up into the cell by the two transporters TrkH (Helo_1371) and TrkI (Helo_1450) [13] and, in salt adapted cells, the cytoplasmic K^+^ concentration reaches a final concentration of approximately 500 mM [11]. The TrkH and TrkI membrane proteins, including the auxiliary protein TrkA, could not be detected in the *H*. *elongata* proteome. However, the putative potassium efflux system KefB (Helo_3903) could be quantified and was shown to be down regulated at elevated salinities, which in addition to potassium uptake, contributes to maintaining a potassium gradient. The membrane protein Helo_2045, which was formerly annotated as KefA, is also down regulated at elevated salinity. It was shown for the related KefA from *E*. *coli* that this protein functions as a K^+^ regulated mechanosensitive channel (now called MscK) [53] and we have re-annotated Helo_2045 accordingly.

### Ectoine metabolism and transport

The enzymes EctB (aspartate-semialdehyde transaminase, Helo_2589), EctA (diaminobutyrate acetyltransferase, Helo_2588) and EctC (ectoine synthase, Helo_2590), which catalyze the conversion of aspartate-β-semialdehyde into ectoine [7, 14], are up regulated in cells grown at 1 M NaCl (6%) compared to cells from 0.1 M NaCl (0.6%). The protein level of EctB was shown to remain elevated at 2 M NaCl. EctA and EctC escaped detection at 2 M NaCl (12%). However, it can be assumed that both enzymes remain elevated at high salinity because the cytoplasmic ectoine concentration increases linearly along with the salt concentration of the medium [54] and the transcription of all three genes, *ectABC*, significantly increases at high salinity.

Ectoine is catabolized by the enzymes for *d*egradation *o*f *e*ctoine (Doe). This pathway allows its utilization as a carbon and nitrogen source by *H*. *elongata* [17]. The degradation (Fig 1) proceeds via Nα-acetyl-diaminobutyric acid to diaminobutyric acid (DABA). DABA can either flow off to aspartate or re-enter the ectoine synthesis pathway. This closes the cycle of synthesis and degradation, which is powered by acetylation and deacetylation of DABA and Nα-Ac-DABA, respectively. The enzymes DoeA (ectoine hydrolase, Helo_3665), DoeB (Nα-acetyl-L-2,4-diaminobutyrate deacetylase, Helo_3664) and DoeD (L-2,4-diaminobutyrate transaminase, Helo_3661) are up regulated at 0.1 M NaCl (0.6%) compared to 1 M NaCl. Whether the protein content of DoeA and DoeB is further regulated at 2 M NaCl (12%) cannot be stated at the moment, since both enzymes escaped detection at 2 M NaCl. However, the transcription of the genes *doeA*, *doeB*, and *doeD* is down regulated with increasing salinity showing a very pronounced decline in transcription from 0.1 M NaCl to 1 M NaCl (0.6% to 6% NaCl).

Aspartate can be generated by transamination of oxaloacetate and is a biosynthetic precursor of ectoine. Interestingly, the enzymes aspartokinase (ASK) (LysC, Helo_3742) and aspartate-β-semialdehyde dehydrogenase (Asd, Helo_2235), leading from aspartate to aspartate-β-semialdehyde, are not affected by increasing salinity. In many organisms, the ASK is an important target for regulating the synthesis of amino acids that belong to the aspartate family [53]. *H*. *elongata* possesses only one ASK, namely LysC, which belongs to the ASKβ homology subdivision [17]. According to the allosteric-specificity grouping of ASKβ enzymes, LysC from *H*. *elongata* is predicted to be sensitive to allosteric regulation by Thr and Lys [55]. Recently, experimental data have been provided by the Galinski group indicating that LysC in *H*. *elongata* is only feed-back regulated by Thr alone [56]. No connection of LysC to the regulation of the ectoine metabolism could be found so far [17] and the unchanged protein level of LysC at all tested salinities is in agreement with this notion.

*Halomonas elongata* possesses two putative transaminases that can potentially convert oxaloacetate to aspartate namely the aspartate transaminase AspC (Helo_4120) and the aromatic amino acid aminotransferase PhhC (Helo_2252), which may also function as an aspartate transaminase (EC 2.6.1.1) [57, 58]. According to the MS analysis the AspC content remains unchanged at all salinities while PhhC is increase by the factor 2.5 at 1 M NaCl (6%).

The transcription of the ectoine-specific transporter operon *teaABCD* is increased at high salinity according to the RT-qPCR analysis. This is in agreement with previous Northern hybridization experiments, which showed an increased transcription of *teaA* at high salt concentration. The protein content of the substrate binding protein TeaA (Helo_4274) [59, 60] and the regulatory cytoplasmic ATP-binding protein TeaD (Helo_4277) [61] was elevated in cells grown at 1 M NaCl (6%). The two membrane components of the ectoine transporter, TeaB (Helo_4275) and TeaC (Helo_4276), could not be quantified from the membrane proteome. However, since the *teaABCD* genes are organized as an operon [59] there is no reason to doubt that the content of all four Tea proteins is increased at high salinity.

### Ammonia assimilation

Being the entry point for ammonia into metabolism, glutamate is always important, but in the case of *H*. *elongata* this importance is even greater, since glutamate itself acts as an osmoregulatory solute and provides the amino groups for the synthesis of ectoine. Immediately after an osmotic up-shock, *H*. *elongata* increases the cytoplasmic K^+^ concentration by uptake via transporters TrkH and TrkI. Concomitantly, the cytoplasmic glutamate concentration is increased. Contrary to other investigated bacteria [10], the levels of potassium and glutamate in *H*. *elongata* remain increased for an extended period of time (>120 min) even in the presence of ectoine [11]. The dual function of glutamate as an osmolyte and nitrogen source for ectoine synthesis might also explain why the K^+^-glutamate concentration in *H*. *elongata* is increased for more than 2 h following an osmotic up-shock. Together with glutamate, the level of glutamine increases as well and reaches its maximum concentration 15 min after the osmotic up-shock, which is approximately half the concentration of glutamate [11]. However, glutamine increases only transiently and falls back to the level before the osmotic shock after approximately 90 min. The two major pathways for nitrogen assimilation end in glutamate: Glutamate can be synthesized either by glutamate dehydrogenase via reductive amination of α-ketoglutarate or by glutamate synthase (GOGAT, Helo_3752–3753), which, in combination with glutamine synthetase (GS, Helo_1126), allows for high affinity nitrogen assimilation.

#### Glutamate synthase

**Fig 5:**
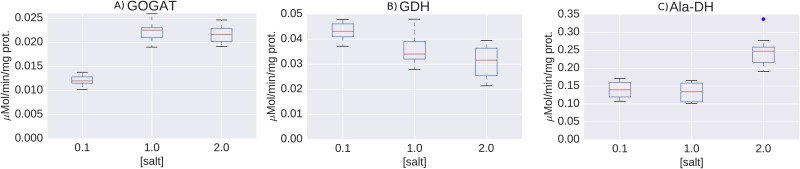
Activity of ammonia assimilating enzymes. A) GOGA, B) Glutamate dehydrogenase (GDH), C) Alanine dehydrogenase (Ala-Dh). Three cultures were grown at each salt concentration (0.1 M, 1 M and 2 M NaCl) and four extracts per culture were prepared (three for Ala-Dh).

Proteomic profiling indicates higher levels of glutamate synthase at higher salt concentrations. The protein level increases at 1 M NaCl (6%) and remains elevated at 2 M NaCl (12%). The same observation was made for the transcription of the glutamate synthase genes, *gltD* and *gltB*. The genes *gltD* and *gltB* encode the small and large subunit of glutamate synthase and are strongly transcribed at high salt concentration. Finally, enzyme assays also show significantly higher activities at high salt concentrations (see Fig 5A).

#### Glutamate dehydrogenase

The expression of glutamate dehydrogenase (*gdh*, Helo_3049) is not up regulated by salinity, according to the proteomics. This was confirmed both at the transcriptional level and measuring enzyme activities (see Fig 5B)

#### Alanine dehydrogenase

The proteome data indicate up regulation of the enzyme alanine dehydrogenase (Ald, Helo_3819). Although a certain trend was observed, no statistically significant increase in enzyme activity by salt could be measured. It is, however, noteworthy that the activities of the enzyme were much higher than those of any other enzyme included in this study (see Fig 5C). Although direct extrapolation from *in vitro* to *in vivo* cannot be made, activities one order of magnitude higher definitely deserve attention. Alanine dehydrogenase catalyzes the reductive amination of pyruvate to alanine, and it is a less frequent but also viable reaction for the assimilation of ammonia [62]. The assimilated nitrogen can be transferred from alanine to glutamate by alanine transaminase (AlaA, Helo_2013). Thus, these two enzymes together represent a yet undescribed low affinity nitrogen assimilation system.

The enzyme assays also established the cofactor specificity of the enzymes, showing that both GDH and Ala-DH use NADH instead of NADPH as electron donor, which is unusual but still possible for assimilation reactions [63]. Taken together, all these results show that ammonia assimilation in *H*. *elongata* deviates from the known pattern, probably due to the need of coordinating the multiple roles of glutamate, and deserves more attention.

### Further observations

We want to point to a couple of other osmoregulatory mechanisms that have been identified by analysing the proteome data.

1. There is an up regulation of ribosomal proteins at 1 M NaCl (6%), which indicates a down regulation of protein biosynthesis when cells of *H*. *elongata* encounter low salt stress. From a total of 18 quantified ribosomal proteins, 17 were up regulated at 1 M NaCl (6%). Northern analysis of ribosomal RNA carried out in our laboratory corroborates this finding, which showed an increase in 16S rRNA after a sudden shift in salt concentration from 170 mM (1%) to 690 mM (4%) NaCl [59].
2. The expression of the Hda protein (*h*omologous to *D*na*A* protein [64]; Helo_2294) is down regulated at 1 M NaCl (6%). Hda participates in the regulatory inactivation of DnaA. DnaA is the initiator of chromosome replication at the *oriC* site. Hda (like DnaA) belongs to the AAA^+^ family of ATPases and is thought to stimulate the hydrolysis of ATP bound to DnaA and thereby inactivating DnaA function [65]. This finding indicates a down regulation of DNA replication at low salt. The proteome data obtained for protein synthesis and DNA replication are in agreement with the poor growth of *H*. *elongata* at low salt, which reaches growth rates of only μ≈0.2 h^-1^ at 0.17 M NaCl (1%) compared to μ = 0.4 h^-1^ at 0.5 M to 1 M NaCl (3% to 6% NaCl).
3. *E*. *coli* and other Gram-negative bacteria synthesize so-called osmoregulated periplasmic glucans (OMG) at low osmolarity [66, 67]. In *H*. *elongata*, three enzymes potentially involved in biosynthesis of the core glucans are down regulated at 1 M NaCl (6%) according to the proteome data, namely glucosyltransferase OpgH (Helo_3146), and the paralogous pair OpgG (Helo_3145) and OpgD (Helo_1814). Thus, it seems that *H*. *elongata* also synthesizes OMGs under low osmolarity stress conditions.
4. We found 9 outer membrane proteins (OMP; β-barrel proteins) whose expression is affected by salt and all 9 proteins are up regulated with increasing salinity. The putative sucrose porin ScrY (Helo_3673) is the most strongly regulated OMP and one of the most significantly regulated proteins in this study, which increases its content by a factor of 335 at 1 M (6%) compared to 0.1 M NaCl (0.6%). The up regulated OMP Helo_3304 (*iutA*) belongs to the family of TonB receptor proteins and displays strong similarities to OMPs involved in iron sequestration. The potential role in iron uptake is supported by the fact that gene *iutA* is located next to a gene encoding a putative ferric iron reductase. For *B*. *subtilis* it was shown that cells of this species coping with high salt stress experience iron limitation [68].

### Metabolic modeling

**Fig 6:**
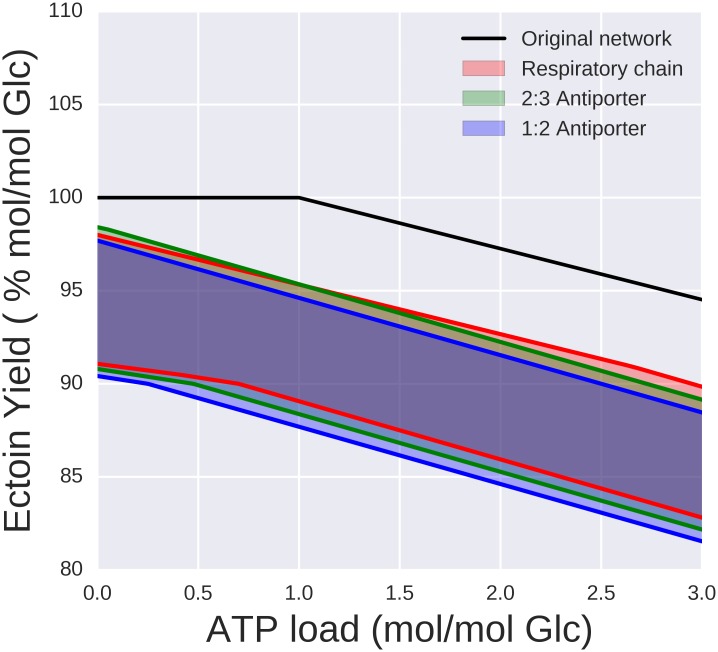
Maximal ectoine yield for different ATP loads (ATP demands external to the network) calculated using Flux Balance Analysis (FBA). The black line shows the potential of the network as inferred from the genome. These yields correspond to EMP glycolysis, TCA cycle anaplerosis through the TCA cycle and ammonia assimilation through GDH or Ala-DH. The three shaded areas show the maximal yields once the experimental findings of this paper are included as constraints (see text for details).

The new findings reported in this study led to extend and modify a previously reported [17] model of ectoine metabolism (see Material and Methods and Supporting Information Table D in the S3 File). Previous sections have already discussed how the modified model helps to understand different subsystems. The question that remains is how the new information modifies our global view. Fig 6 shows the maximum theoretical yields of ectoine as calculated through FBA for different ATP loads, both before and after modifying the model. The black line shows the theoretical yield of ectoine calculated by direct optimization of the full network. The optimal flux distributions were in all cases dominated by EMP and subsequent anaplerosis of the TCA cycle by the malic enzyme. These solutions can be understood as the potential of the network. Modifying the model incorporates the following new findings, namely i) the Entner-Doudoroff pathway is the main pathway for glycolysis, ii) a PPi-dependent 6-phosphofructokinase that alters the ATP-balance of the EMP pathway, and iii) the malic enzyme does not catalyze any relevant anaplerotic reaction. These new findings bring the model closer to how the network actually works and changes the results both quantitatively and qualitatively. Quantitatively, the theoretical yields are lower once the above mentioned constraints are included. The difference may not seem too large, and FBA is known to be robust in this sense, but these relatively small variations in yield hide a completely different qualitative behaviour. There is no single dominant solution anymore but a range of possible solutions that can perform better or worse depending on the circumstances. That involves the concerted action of tightly coupled subsystems for instance the anaplerotic pathways and sodium transport in order to work efficiently. The three shaded areas in Fig 6 show the performance of the network depending on which sodium transport mechanism is active. The upper line in each case is the yield when all ammonia is assimilated through AlaDH or GDH while the lower line corresponds to full assimilation through GOGAT.

### Concluding remarks

Quantitative proteome profiling analysis combined with RT-qPCR analysis and enzyme activity measurements of *H*. *elongata* revealed that not only the obvious mechanisms involved in osmoadaptation are affected by salt (such as compatible solute transport, sodium efflux etc.), but even in central metabolism osmoregulatory changes occur. Most eminent in our opinion, is the necessary shift of carbon flow towards oxaloacetate and the interplay between anaplerotic enzymes and sodium transport. This finding points to a different regulation of ectoine synthesis in *H*. *elongata* compared to less salt tolerant ectoine producers. The formation of aspartate semialdehyde is often the regulatory step in synthesis of aspartate-derived amino acids and many microorganisms have evolved different types of aspartokinase in order to fine-tune these pathways [53]. A number of bacteria synthesizing ectoine possess a specialized aspartokinase ASK-Ect, which is less prone to feedback regulation (by threonine) at high salinity and seems to be adapted to the synthesis of ectoine [69]. *H*. *elongata* (and related halophiles such *C*. *salexigens*) does not possess aspartokinase ASK-Ect and only relies on aspartate kinase LysC. There is no evidence indicating a role of LysC in osmoregulating ectoine synthesis. Based on our investigation we propose that in *H*. *elongata* the significant (osmo)-regulatory event occurs earlier in the metabolic pathway namely at the PEP-pyruvate-oxaloacetate node. This view is also supported by our metabolic modeling.

Our experimental and modelling results indicate that the tandem of anaplerotic enzymes Oad and PEP carboxylase is the most efficient anaplerotic pathway for ectoine synthesis while the glyoxylate shunt is the worst. This explains the inverse correlation in the regulation of these two. Under low salt, ectoine synthesis is not a priority and the glyoxylate shunt (and maybe low activities of the other anaplerotic reactions) provides enough material for biosynthetic processes branching out of oxaloacetate. Upon increases in the salinity of the medium, the anaplerotic reactions catalyzed are up regulated and the glyoxylate shunt fades out.

With respect to the glycolytic pathways, there are some indications (for instance, PPi-dependent phosphofructokinase) that the EMP-pathway might not be active in *H*. *elongata* except for gluconeogenesis and glucose degradation most likely proceeds solely via the ED-pathway, as reported for *C*. *salexigens* [26].

Diverting the carbon flow as an osmoregulatory mechanism implies to throttle back glucose degradation at low salinity to avoid dissipation of carbon source. Our results are in agreement with this notion and indicate down regulation of the ED-pathway at low salinity. However, it might be arguable how well the regulatory mechanisms function at low salt. The work on the metabolic fluxes in *C*. *salexigens* revealed a certain metabolic rigidity that leads to metabolic overflow at low salt [26]. It is known that *H*. *elongata* performs only poorly at low salt and growth rates at that salinity are as low as at 2 M NaCl (12%). In this context, the regulation of certain processes at low salt (0.1 M NaCl, 0.6%) such as periplasmic glucose dehydrogenase (ED-pathway), down regulation of protein biosynthesis and DNA replication can be interpreted as *low salt stress* adaptation.

The combination of high throughput techniques and metabolic reconstructions makes a powerful tool. However, it has to be carefully considered, which of the multiple alternative processes encoded by the genome are indeed active. The tendency of automated tools based on Flux Balance Analysis to find stoichiometrically optimal solutions may shift the focus away from the really important processes. This was shown in the present paper in the case of the pathways for glucose catabolism, nitrogen assimilation and anaplerotic reactions. With the help of a few well-targeted experiments, we can guide the application of these modelling tools and achieve a much better understanding of the metabolic network that is actually operating in *H*. *elongata*. This leads to a completely different list of priorities for the development of industrial producer strains of *H*. *elongata*.

## Supporting Information

S1 File Supporting Information PCR. Figures A to W and Table A.
(DOCX)

S2 File Supporting Information Proteome. Tables B and C.
(DOCX)

S3 File Supporting Information Model. Table D.
(XLSX)
