# Supplementary material for: Osmoregulation in the Halophilic Bacterium Halomonas elongata: A Case Study for Integrative Systems Biology
Source: PLoS One. 2017 Jan 12;12(1):e0168818. doi: 10.1371/journal.pone.0168818 (PMC5231179; doi:10.1371/journal.pone.0168818)
Supplement: S1 File — Figures A to W and Table A. (DOCX) [file pone.0168818.s001.docx]

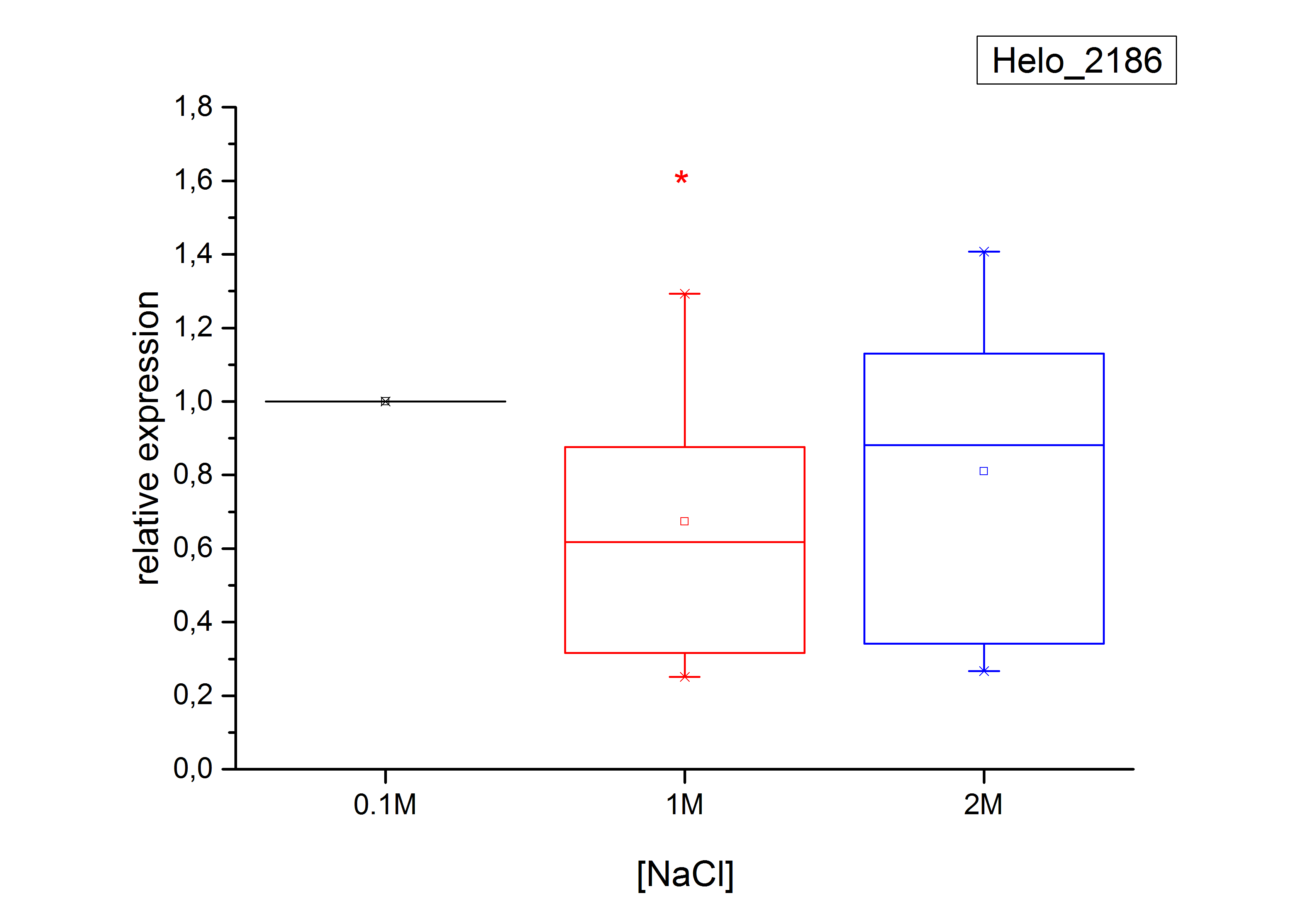


**Figure A. Validation of gene expression by RT-qPCR.**

The relative expression of the *6-phosphofructokinase* gene (Helo_2186) was measured after induction with 1 M and 2 M NaCl compared to a non-induced sample (0.1 M). The results obtained by three independent biological replicate experiments (n=3) are shown here. Boxes represent mean values (small square) ±standard error. Colored asterisks indicate statistical significance of the samples against the control setup (0.1 M), which was converted to 1. Black asterisk indicate statistical significance in between 1 M and 2 M (brackets). All mutant values represent statistically significant relative expression as determined by paired t-test, ^*^P<0.05.

The bar graphs show the medians (line), interquartile ranges (end of boxes), means (squares) and 90% percentiles (whiskers). Different asterisk above plots indicate statistically significant (P=0.05) differences among experimental setup.


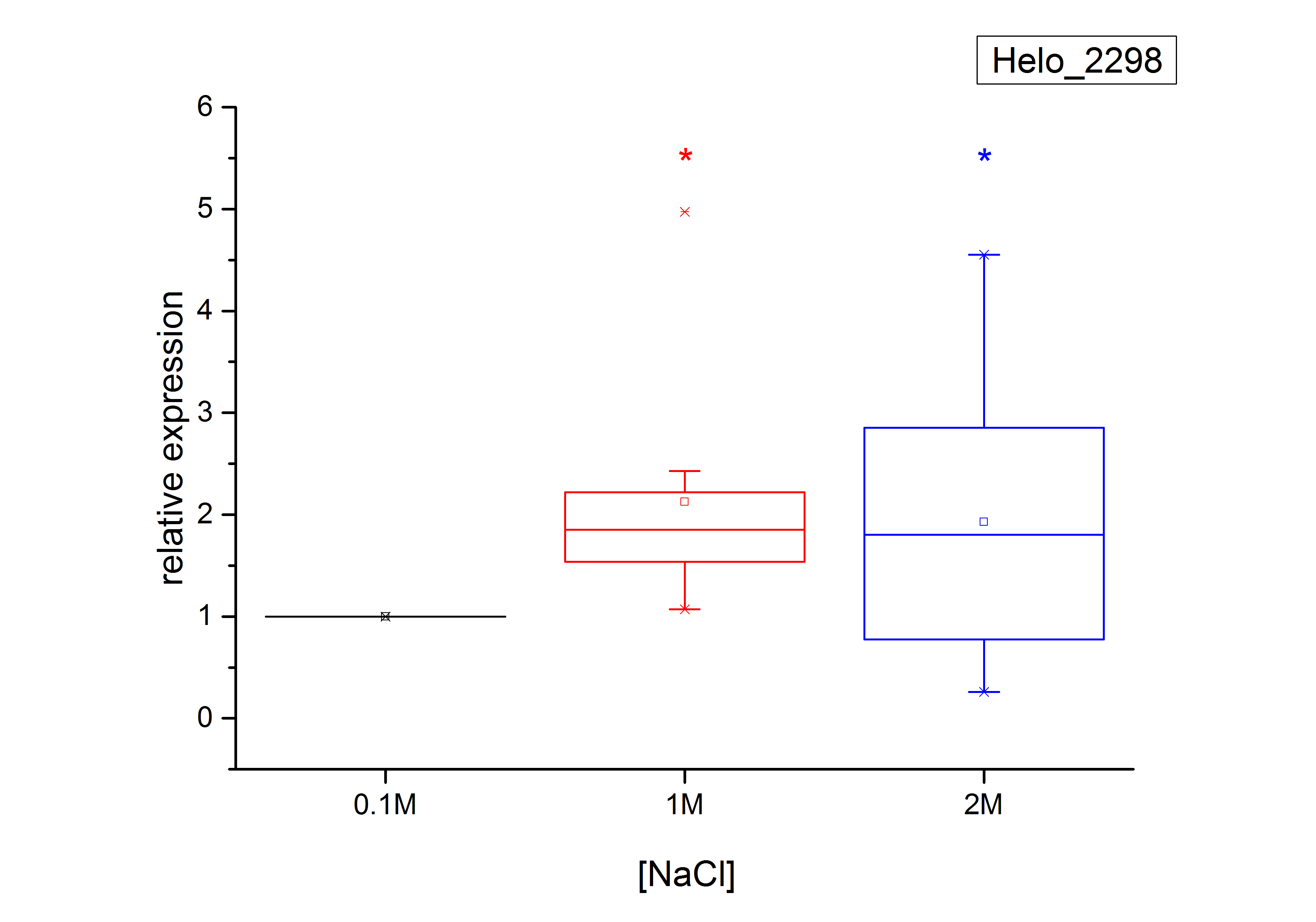


**Figure B. Validation of expression of the fumarase gene by RT-qPCR.**

For details see Figure A.


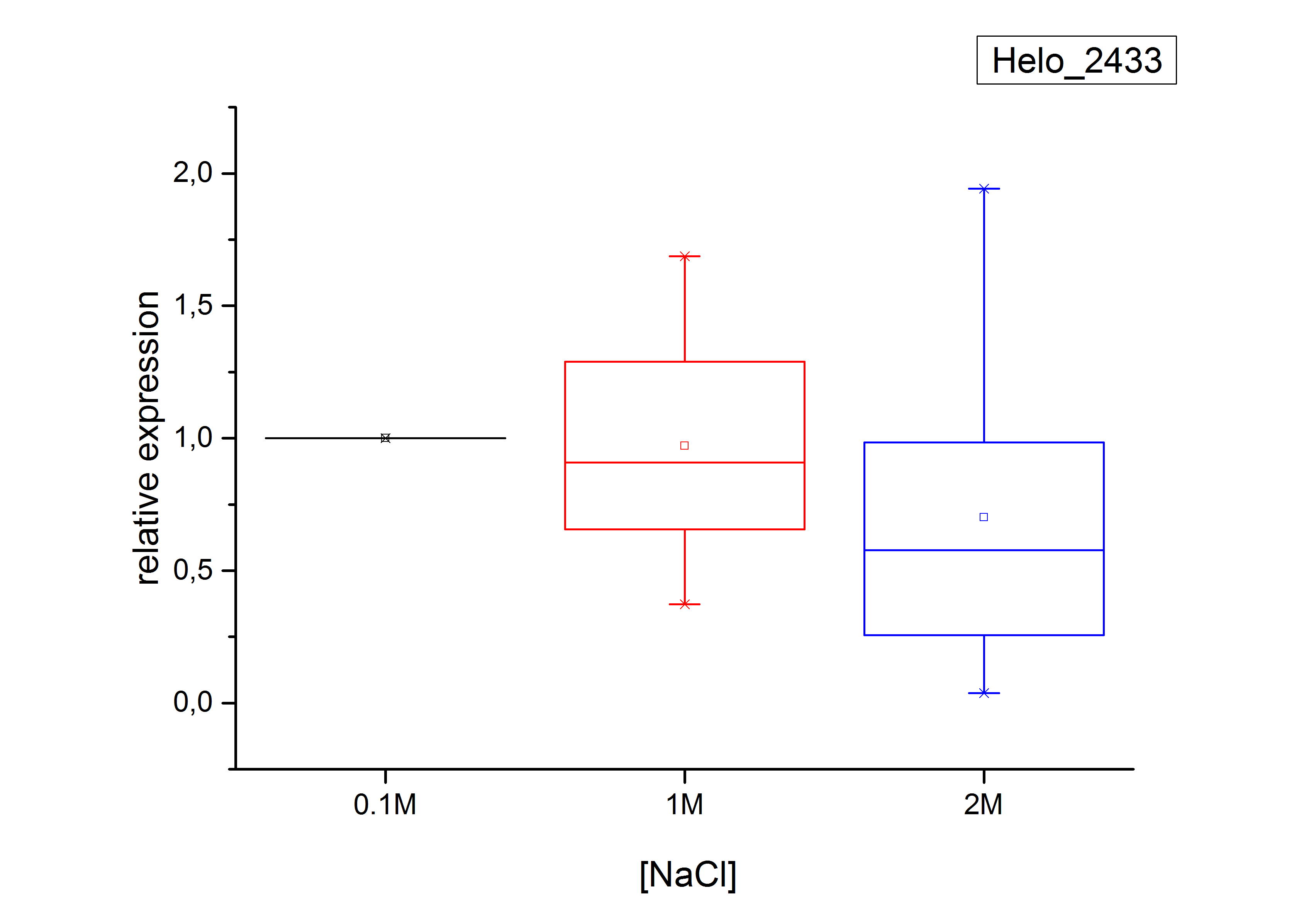


**Figure C. Validation of expression of the phosphoenolpyruvate synthase gene by RT-qPCR.**

For details see Figure A.


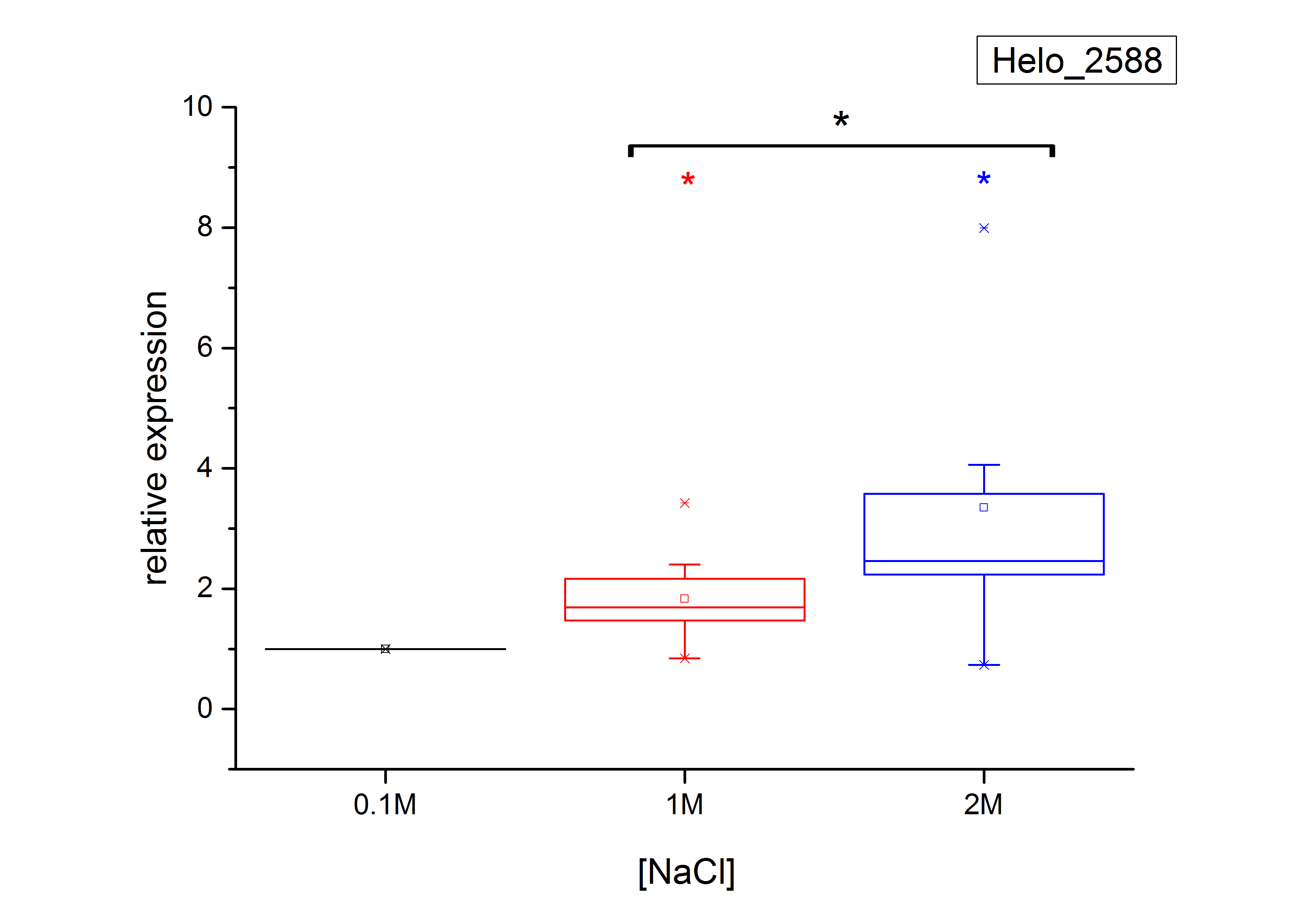


**Figure D. Validation of expression of the L-2,4-diaminobutyrate acetyltransferase (*ectA*) gene by RT-qPCR.**

For details see Figure A.


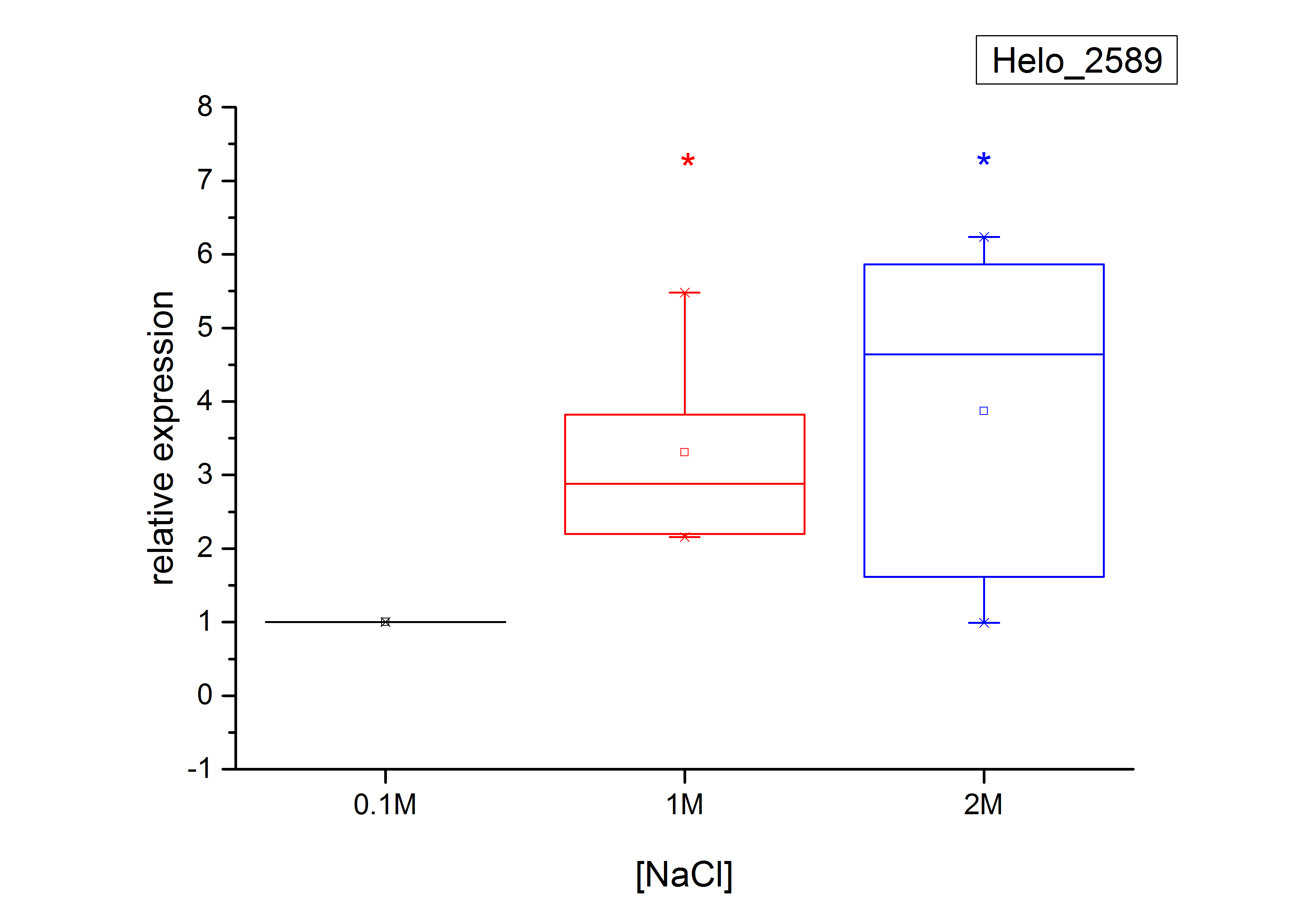


**Figure E. Validation of expression of the aspartate-semialdehyde transaminase gene (*ectB*) by RT-qPCR.**

For details see Figure A.


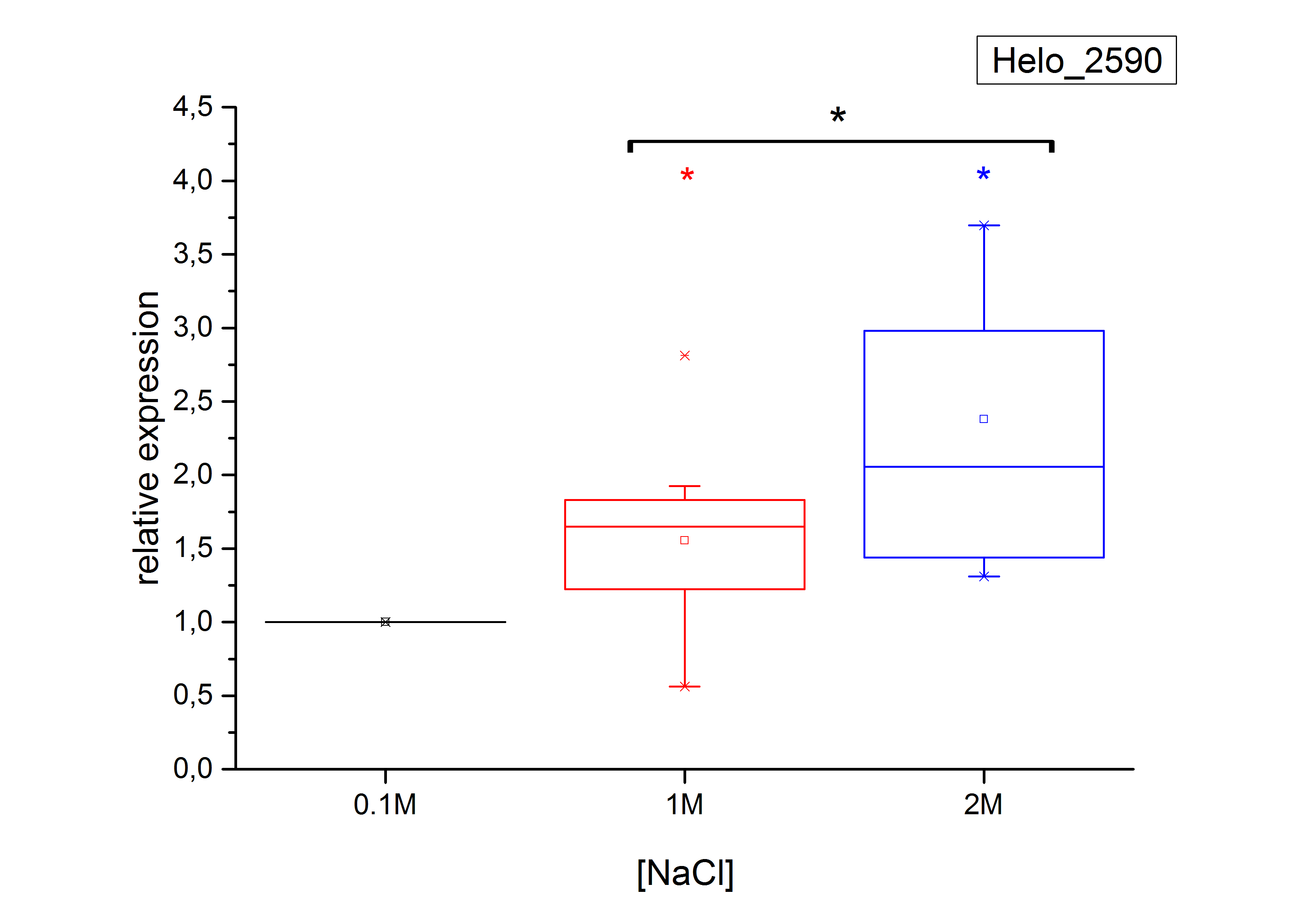


**Figure F. Validation of expression of the ectoine synthase gene (*ectC*) by RT-qPCR.**

For details see Figure A.


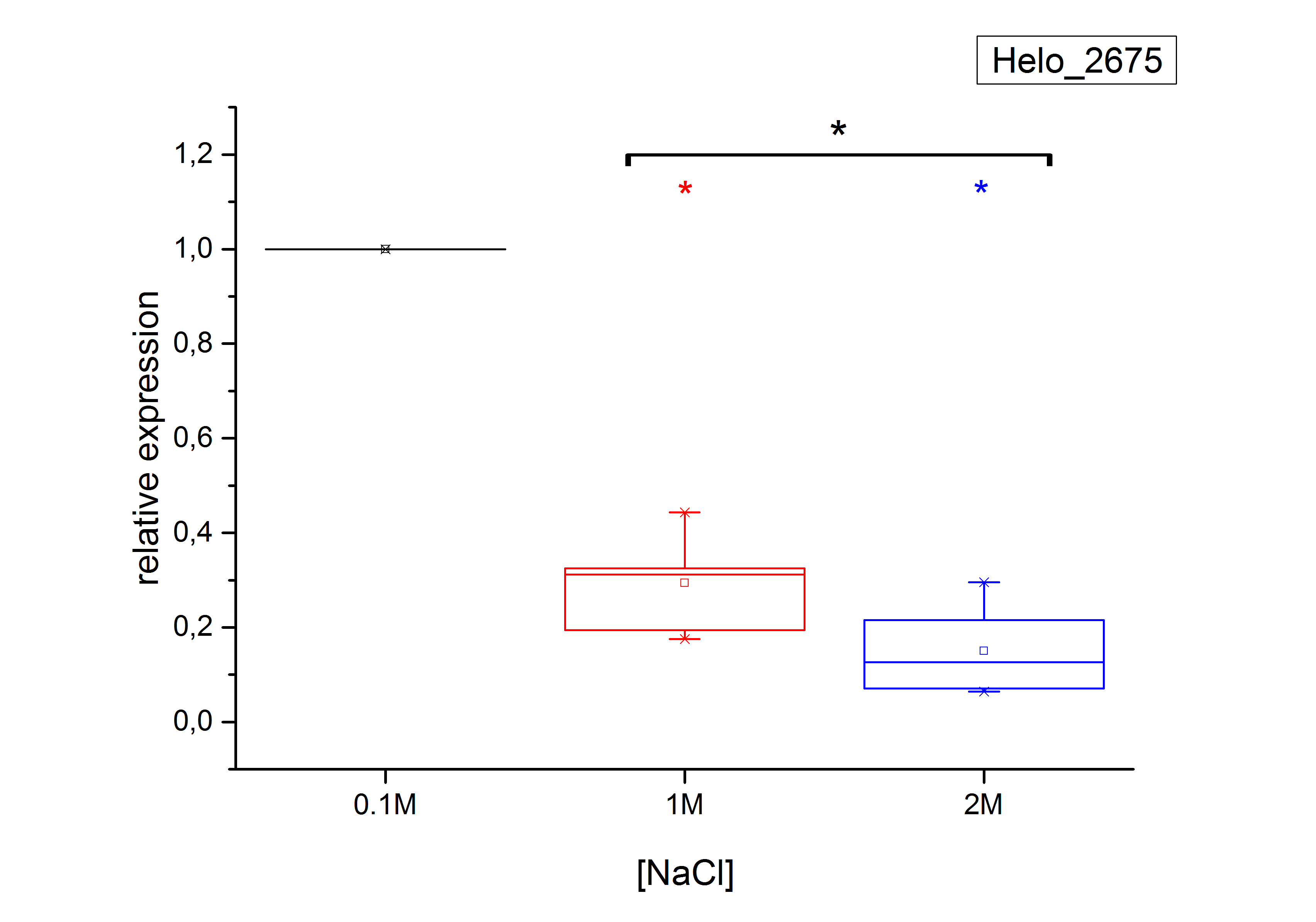


**Figure G. Validation of expression of the ornithine carbamoyltransferase gene by RT-qPCR.**

For details see Figure A.


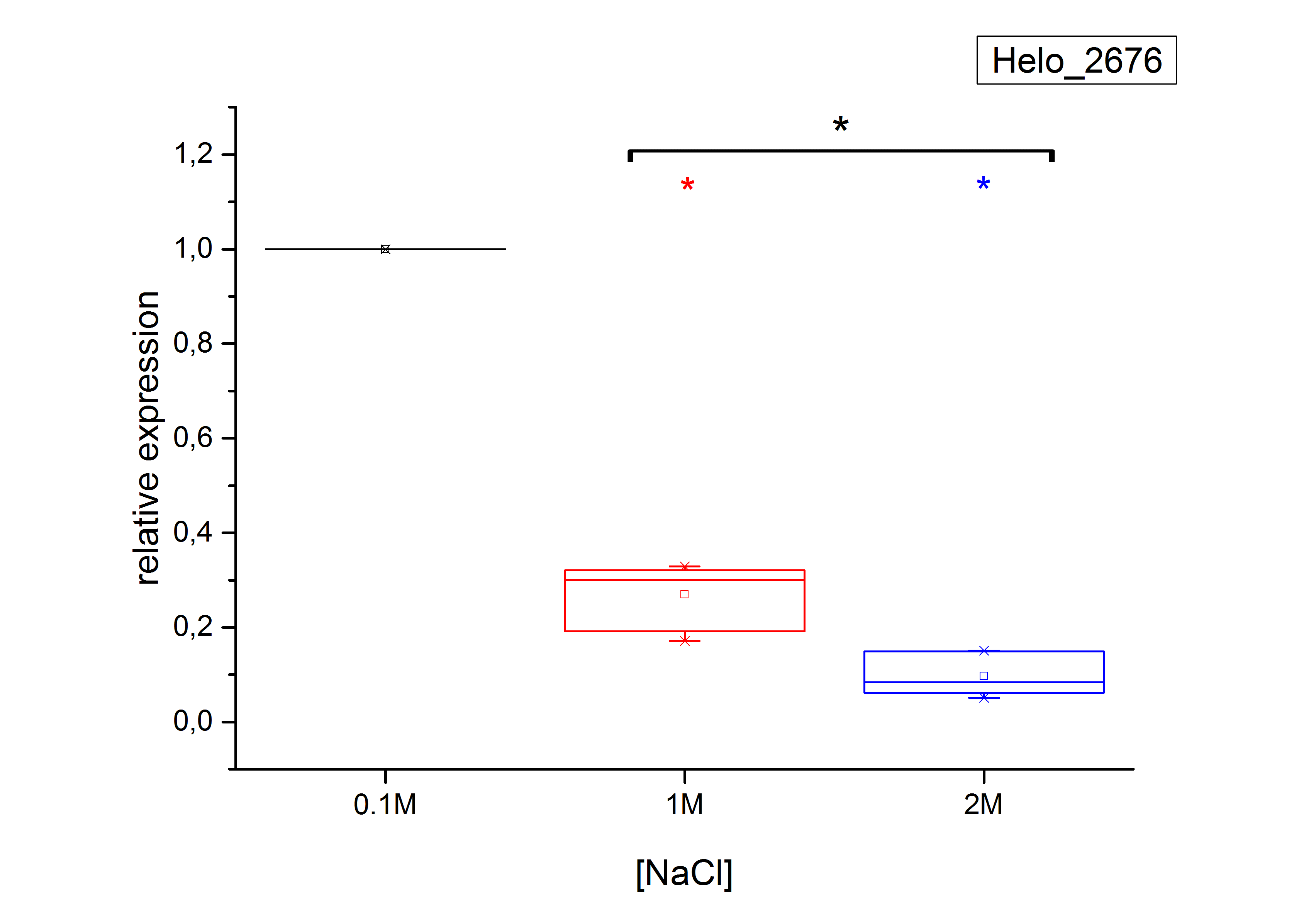


**Figure H. Validation of expression of the arginine deiminase gene by RT-qPCR.**

For details see Figure A.


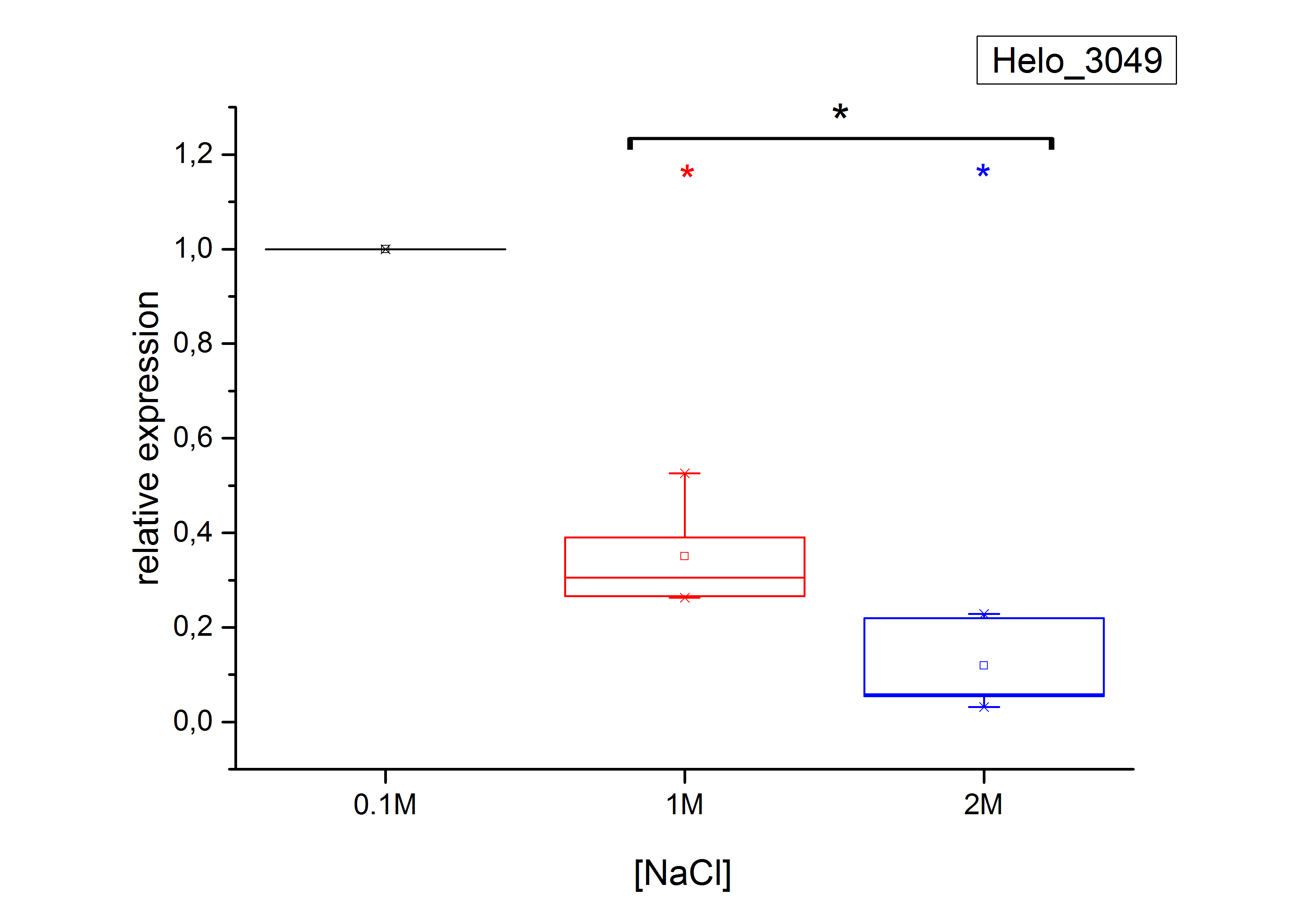


**Figure I. Validation of expression of the glutamate dehydrogenase gene by RT-qPCR.**

For details see Figure A.


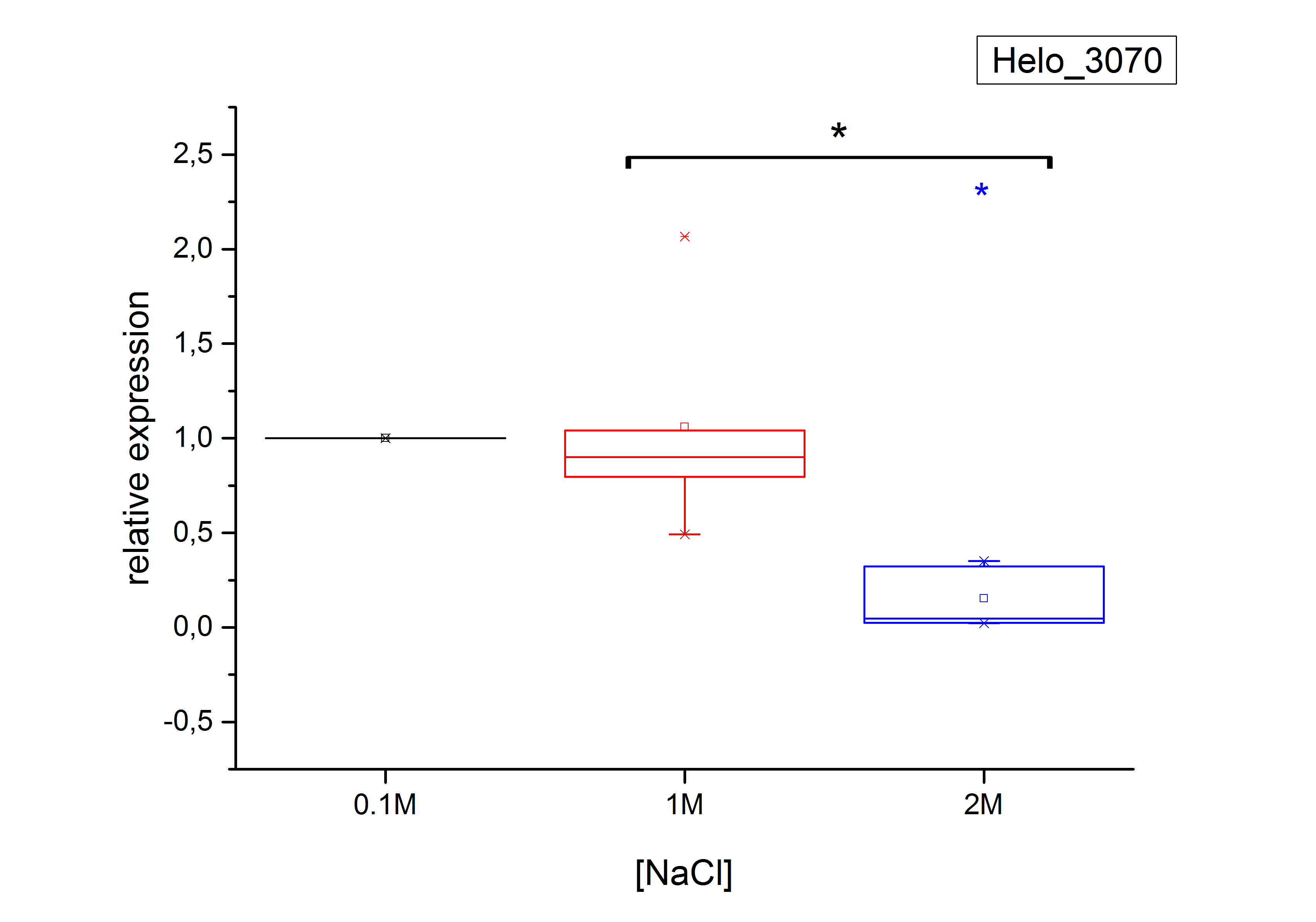


**Figure J. Validation of expression of the isocitrate lyase gene by RT-qPCR.**

For details see Figure A.


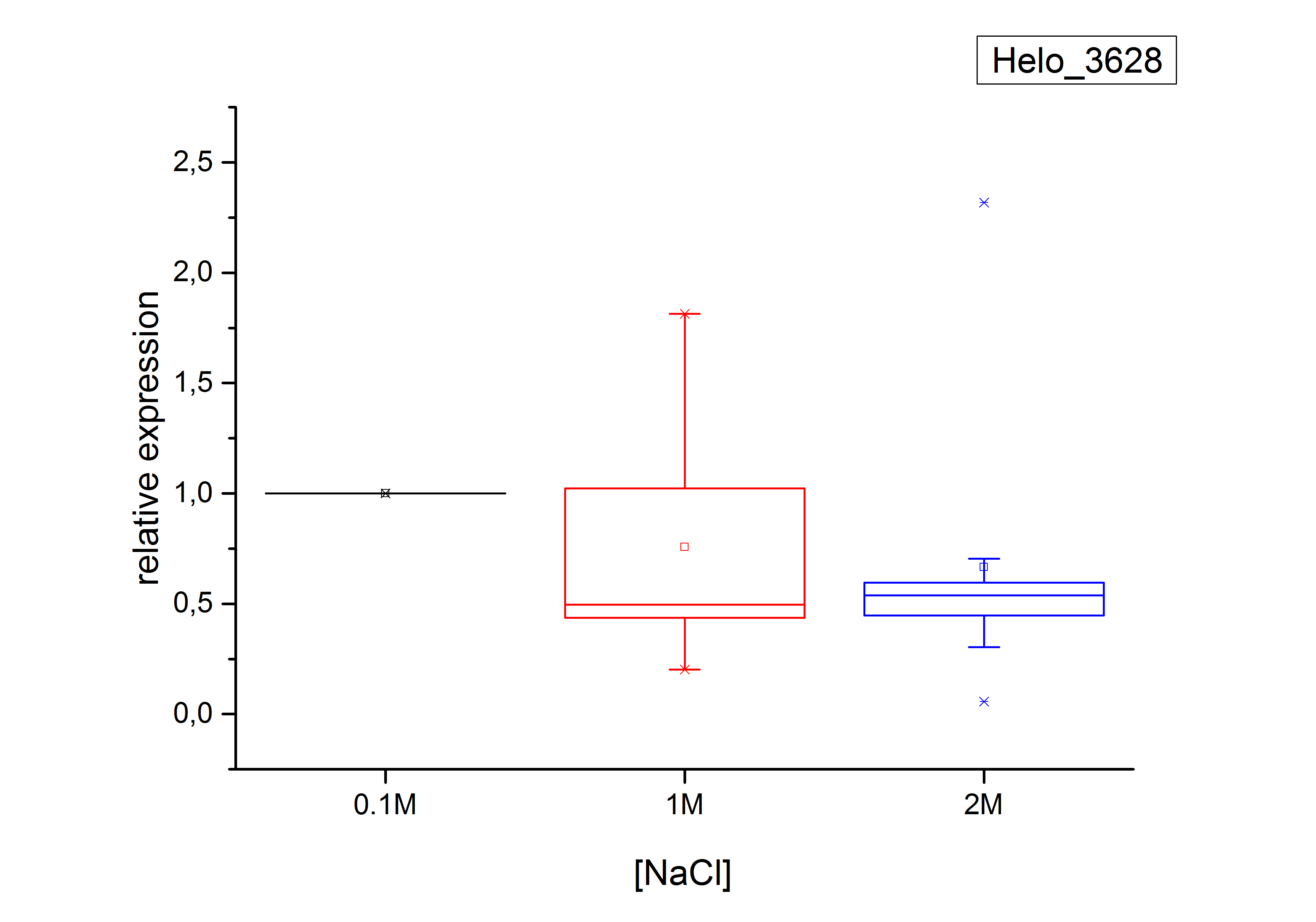


**Figure K. Validation of expression of the** **phosphogluconate dehydratase gene by RT-qPCR.**

For details see Figure A.


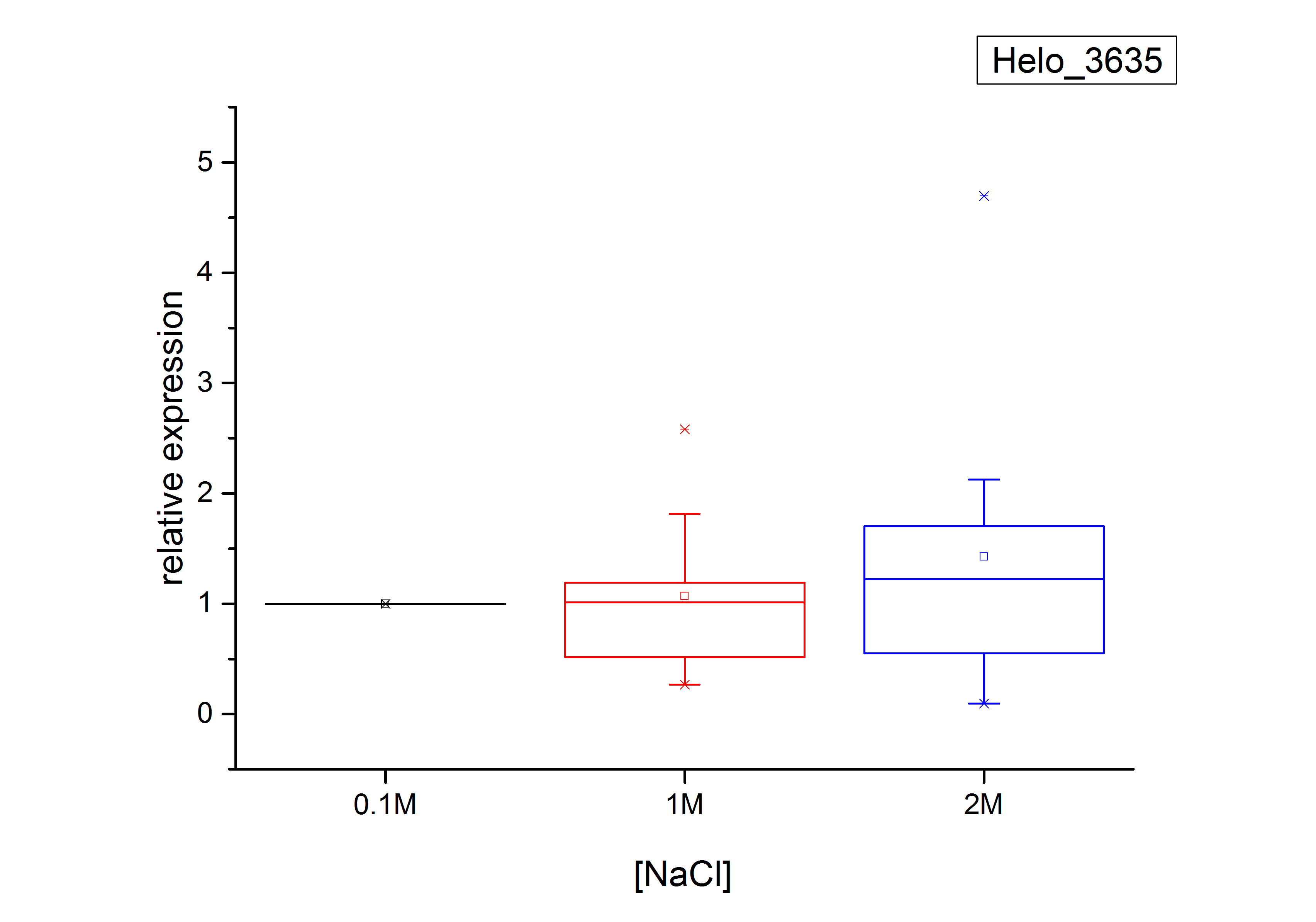


**Figure L. Validation of expression of the** **2-keto-3-deoxy-phosphogluconate aldolase gene by RT-qPCR.**

For details see Figure A.


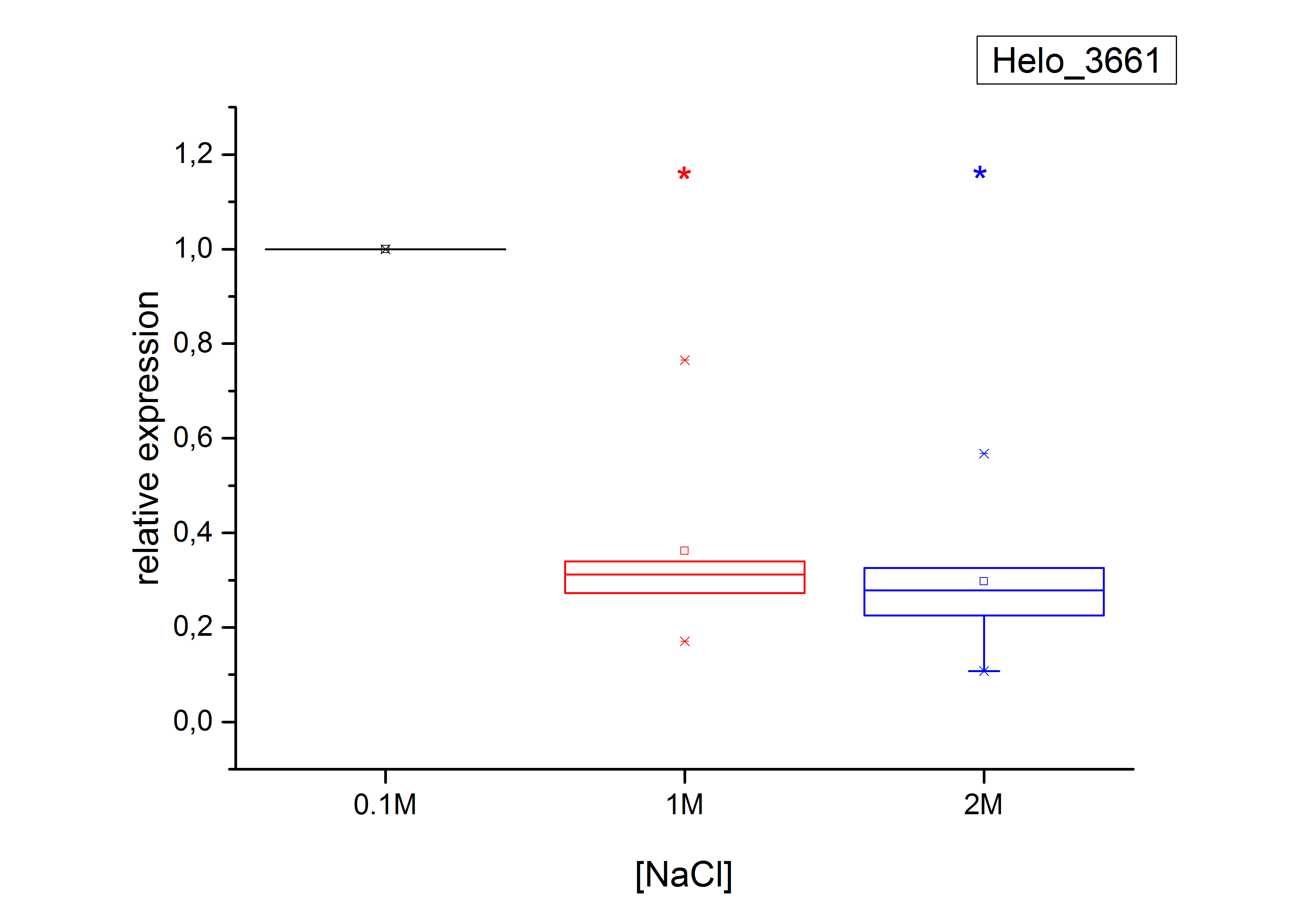


**Figure M. Validation of expression of the L-2,4-diaminobutyrate transaminase gene (*doeD*) by RT-qPCR.**

For details see Figure A.


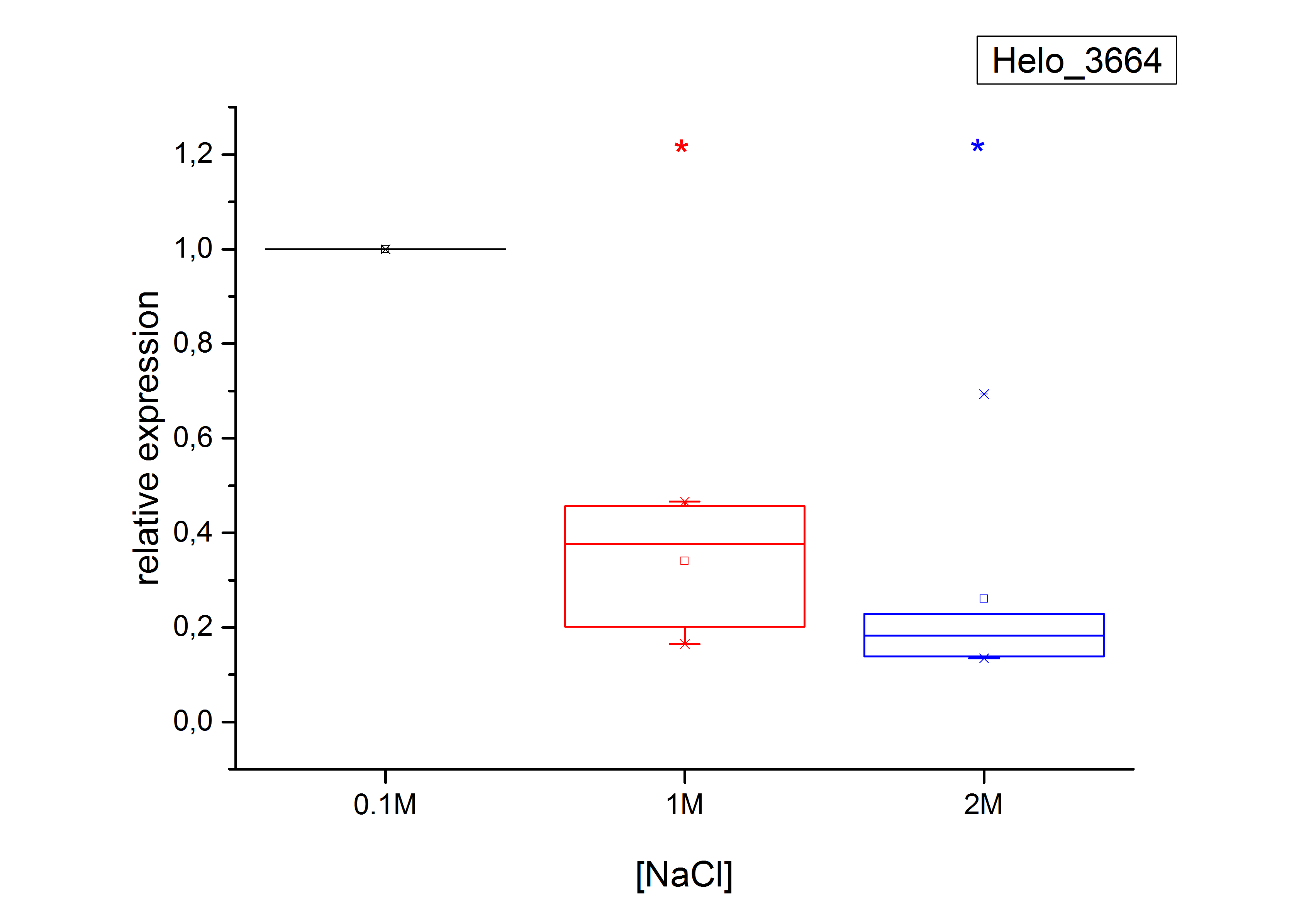


**Figure N. Validation of expression of the** **Nα-acetyl-L-2,4-diaminobutyrate deacetylase gene (*doeB*) by RT-qPCR.**

For details see Figure A.


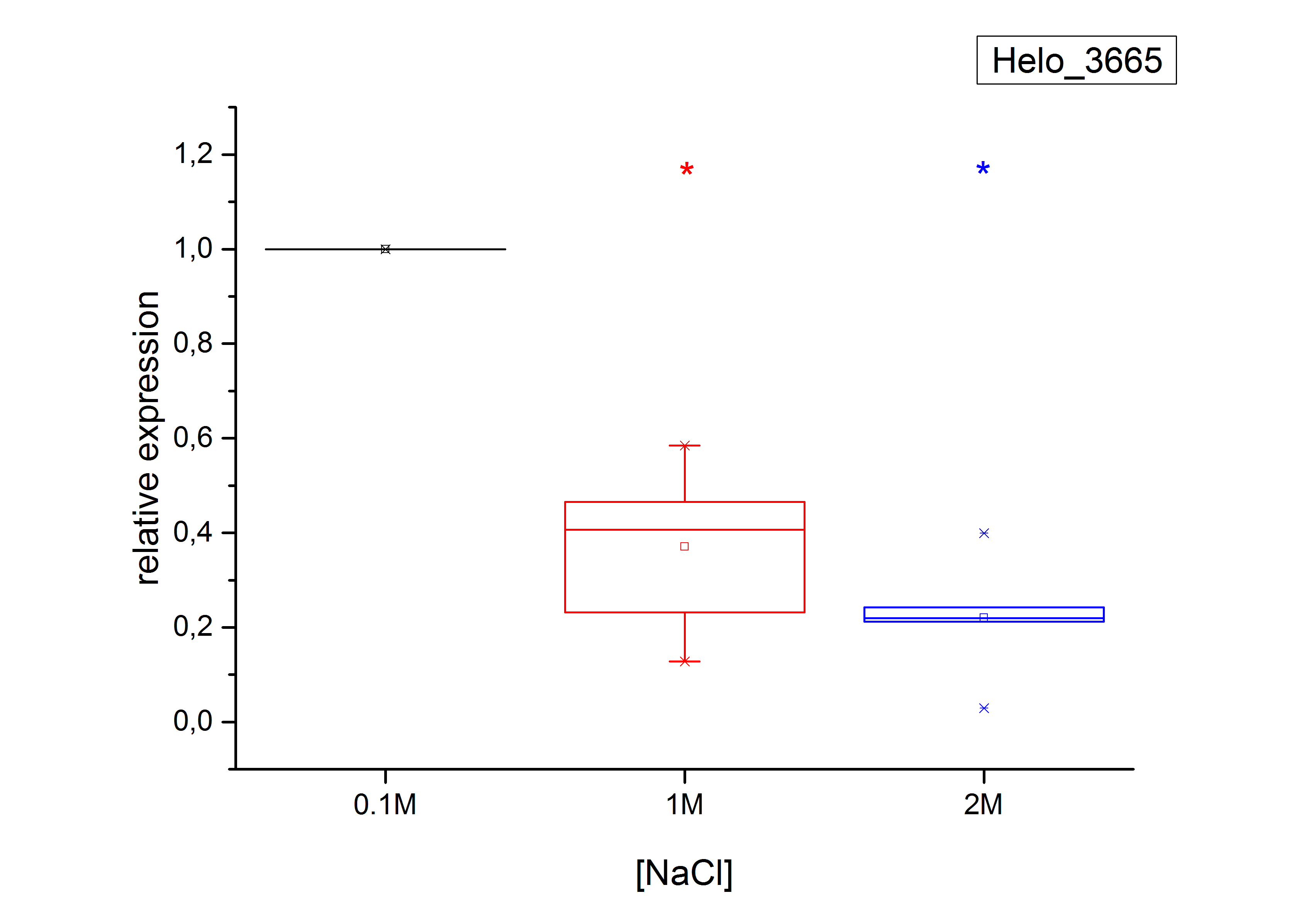


**Figure O. Validation of expression of the** **ectoine hydrolase gene (*doeA*) by RT-qPCR.**

For details see Figure A.


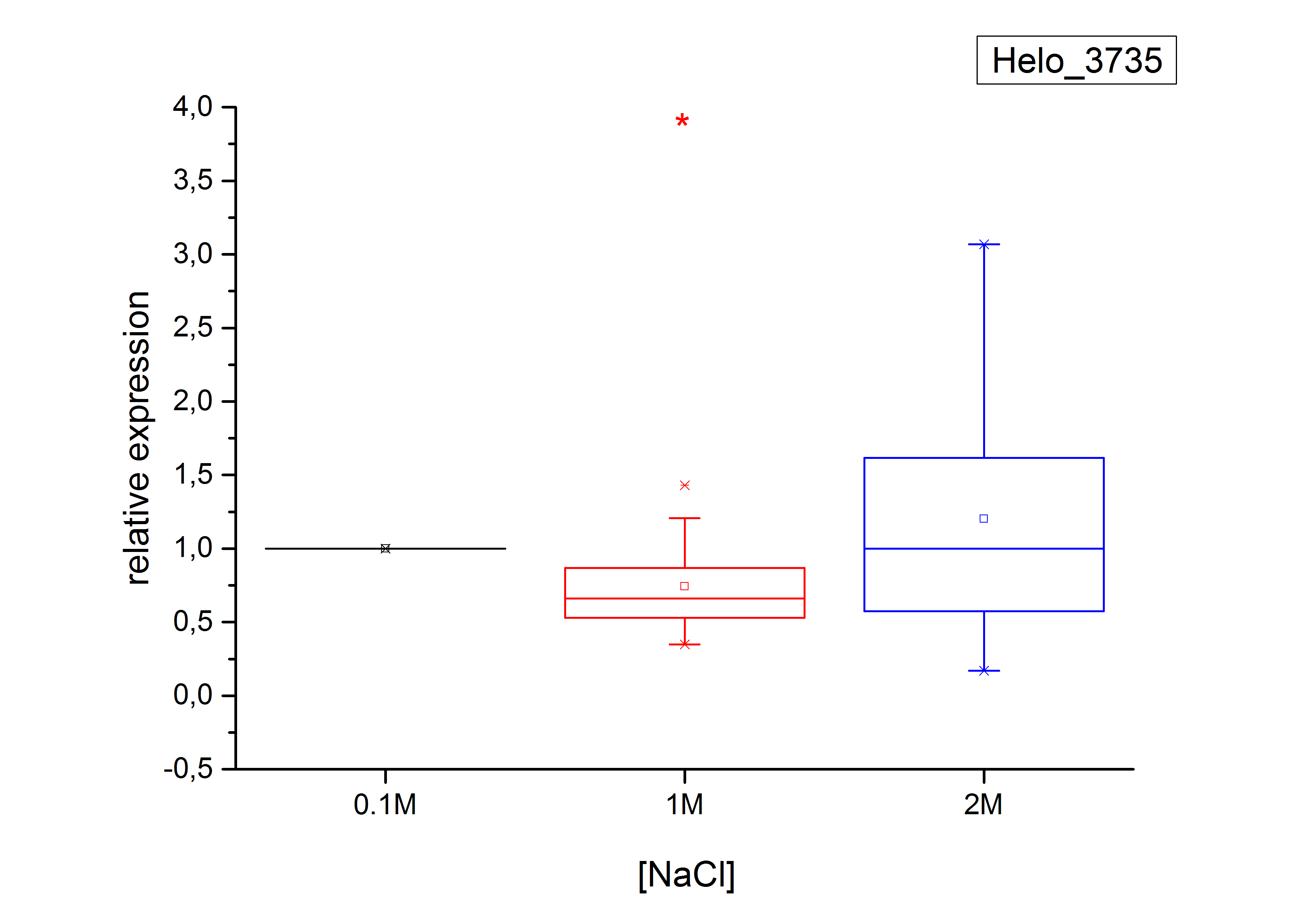


**Figure P. Validation of expression of the oxaloacetate decarboxylase (alpha subunit) gene by RT-qPCR.**

For details see Figure A.


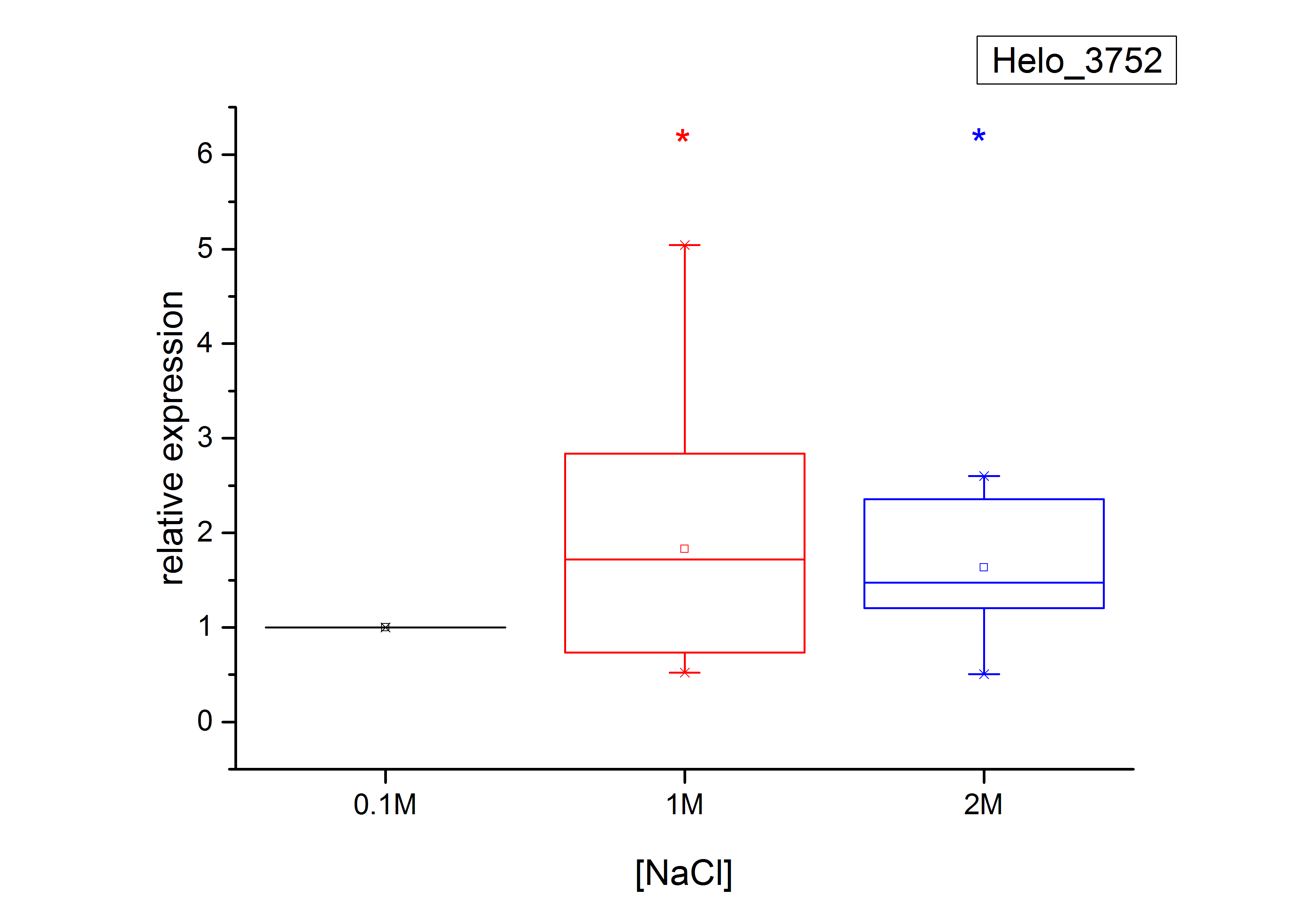


**Figure Q. Validation of expression of the glutamate synthase gene (small subunit) by RT-qPCR.**

For details see Figure A.


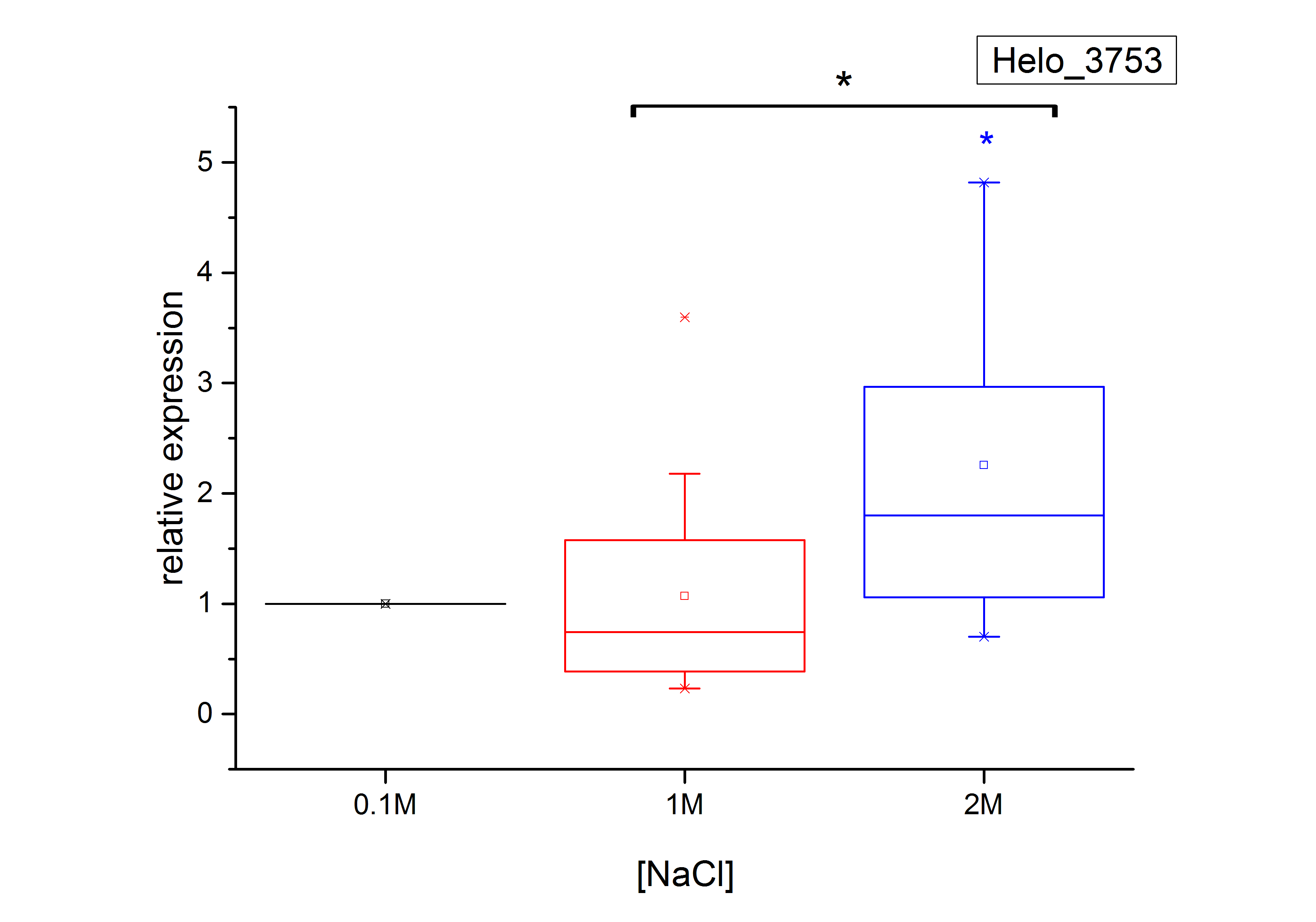


**Figure R. Validation of expression of the glutamate synthase gene (large subunit) by RT-qPCR.**

For details see Figure A.


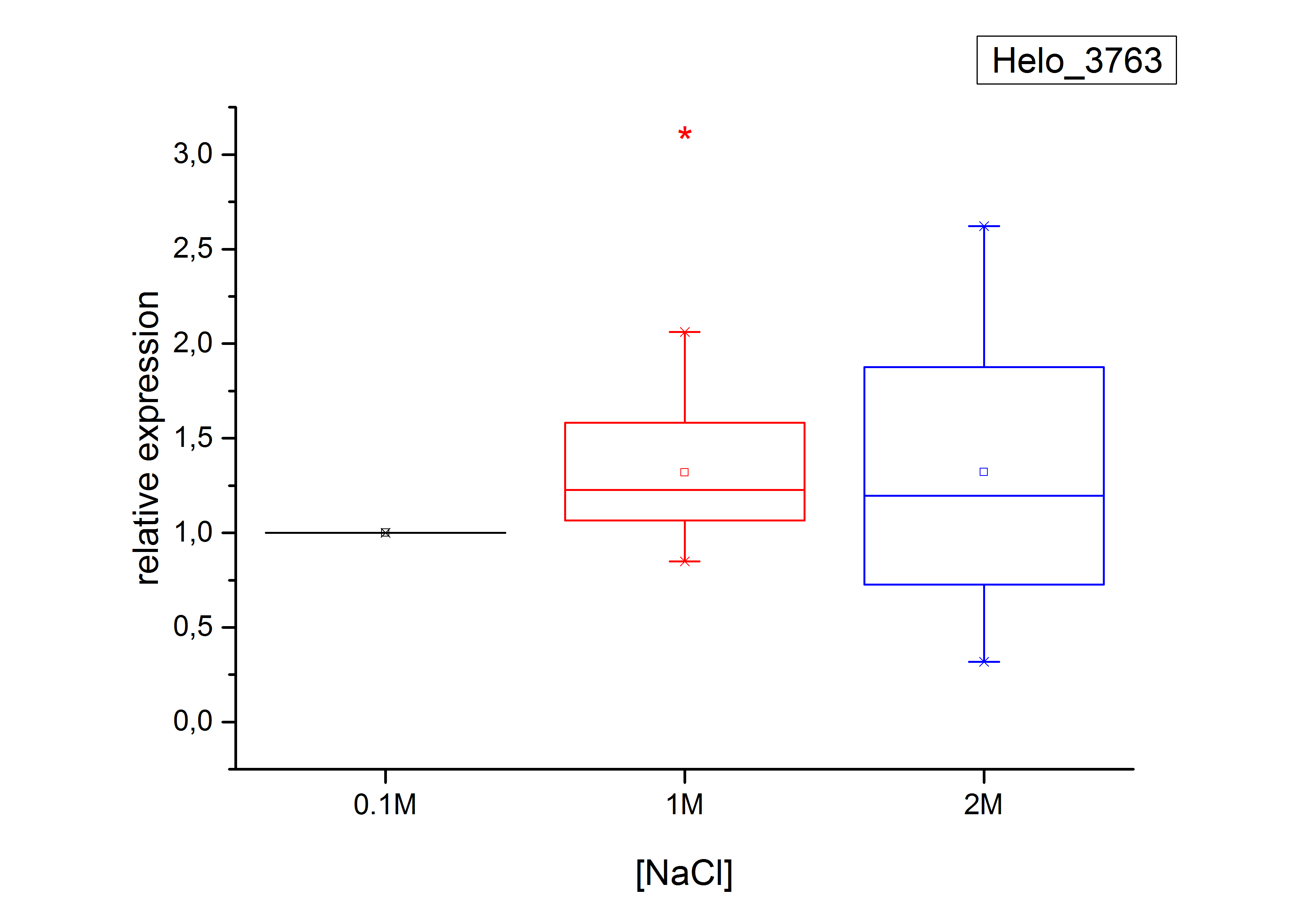


**Figure S. Validation of expression of the malic enzyme gene by RT-qPCR.**

For details see Figure A.


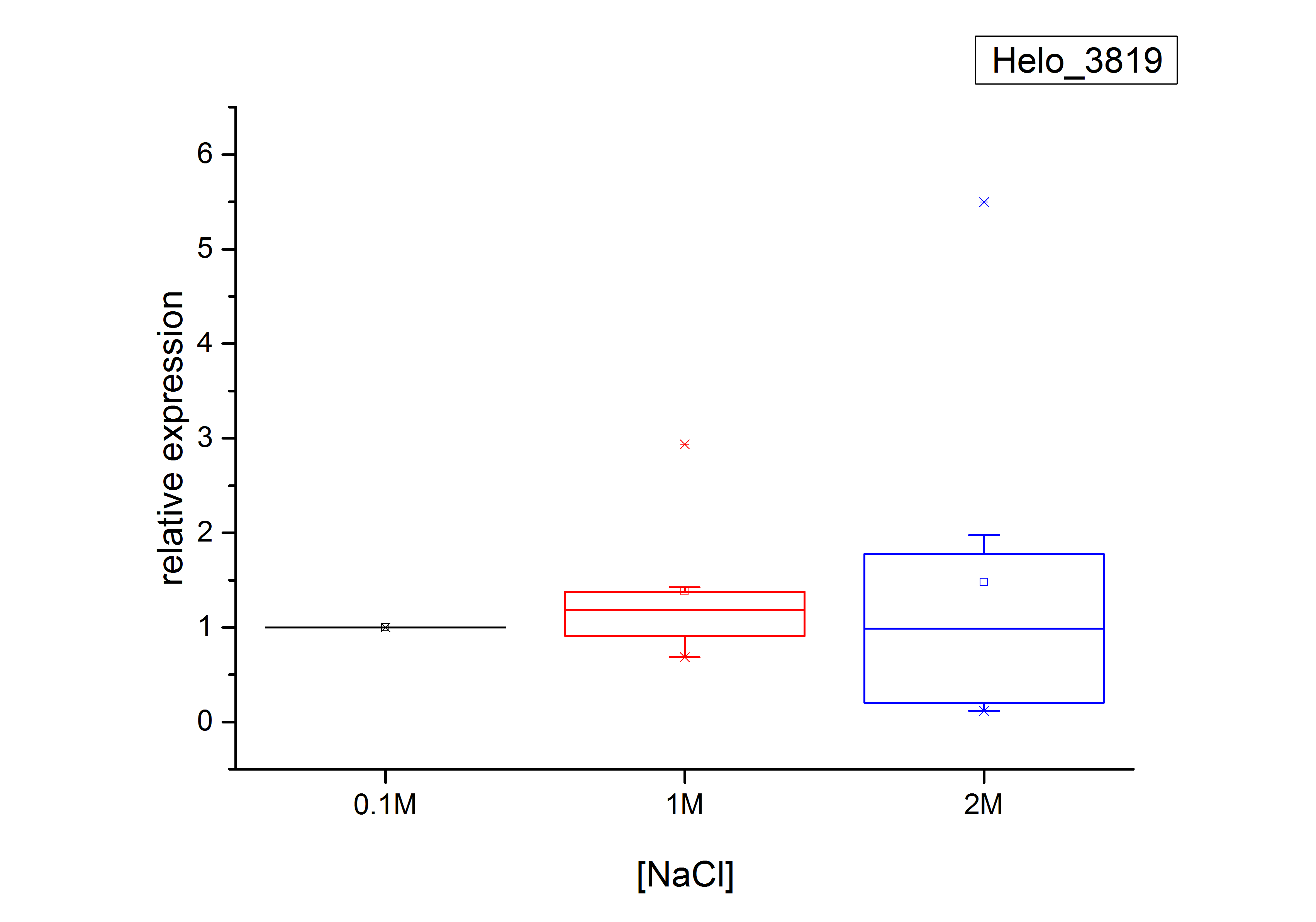


**Figure T. Validation of expression of the alanine dehydrogenase gene by RT-qPCR.**

For details see Figure A.


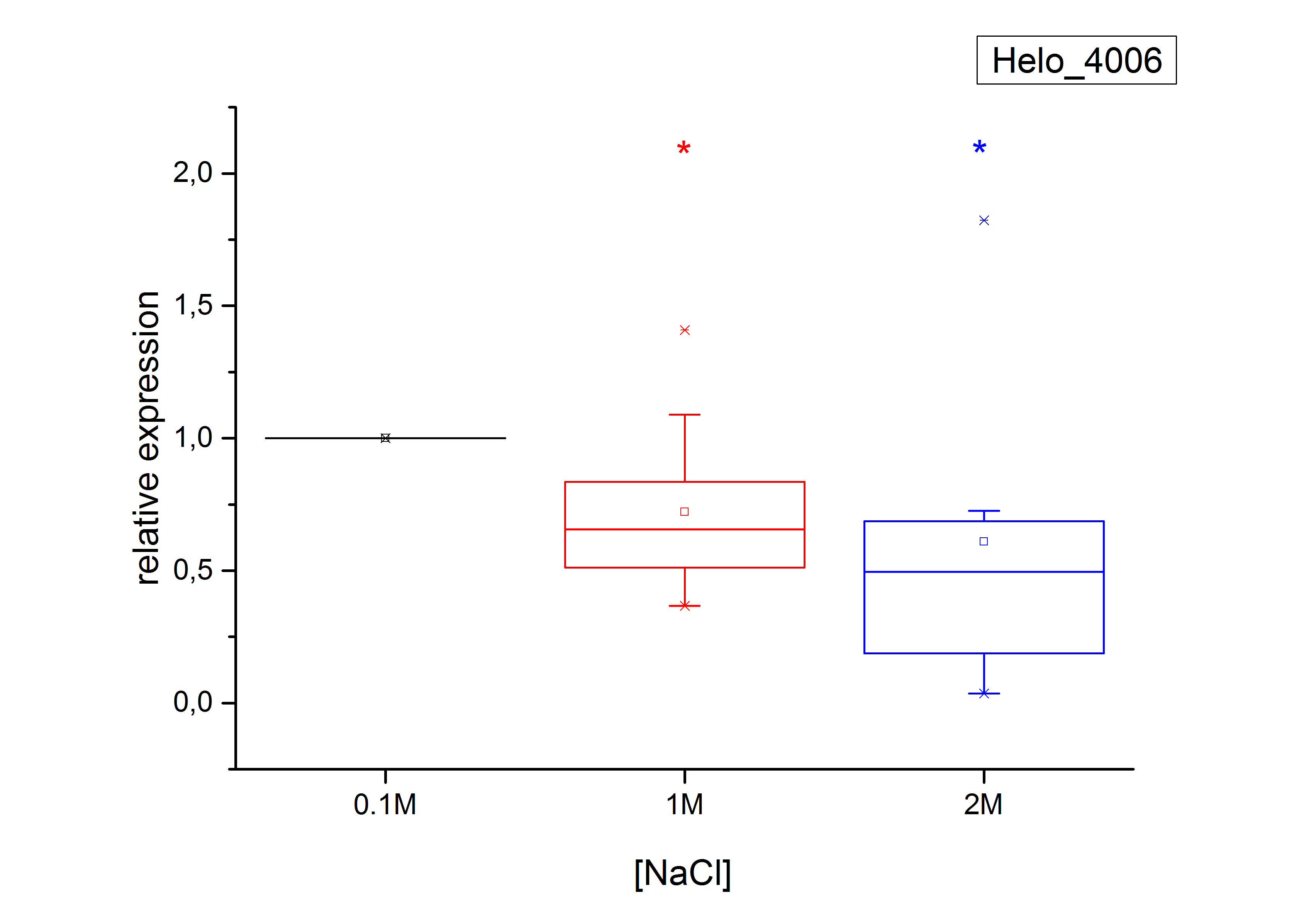


**Figure U. Validation of expression of the glucose dehydrogenase gene by RT-qPCR.**

For details see Figure A.


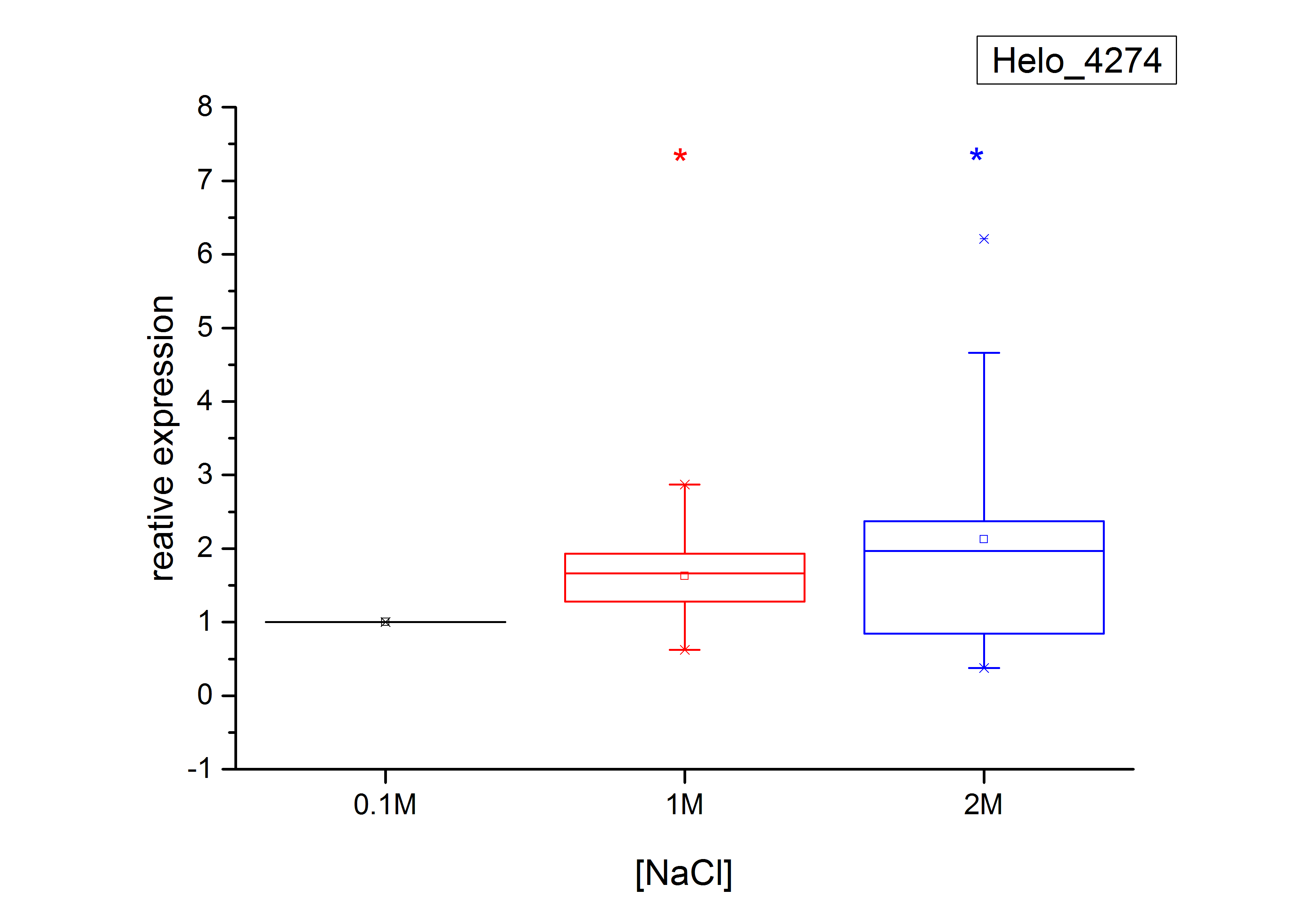


**Figure V. Validation of expression of the substrate-binding protein TeaA gene by RT-qPCR.**

For details see Figure A.


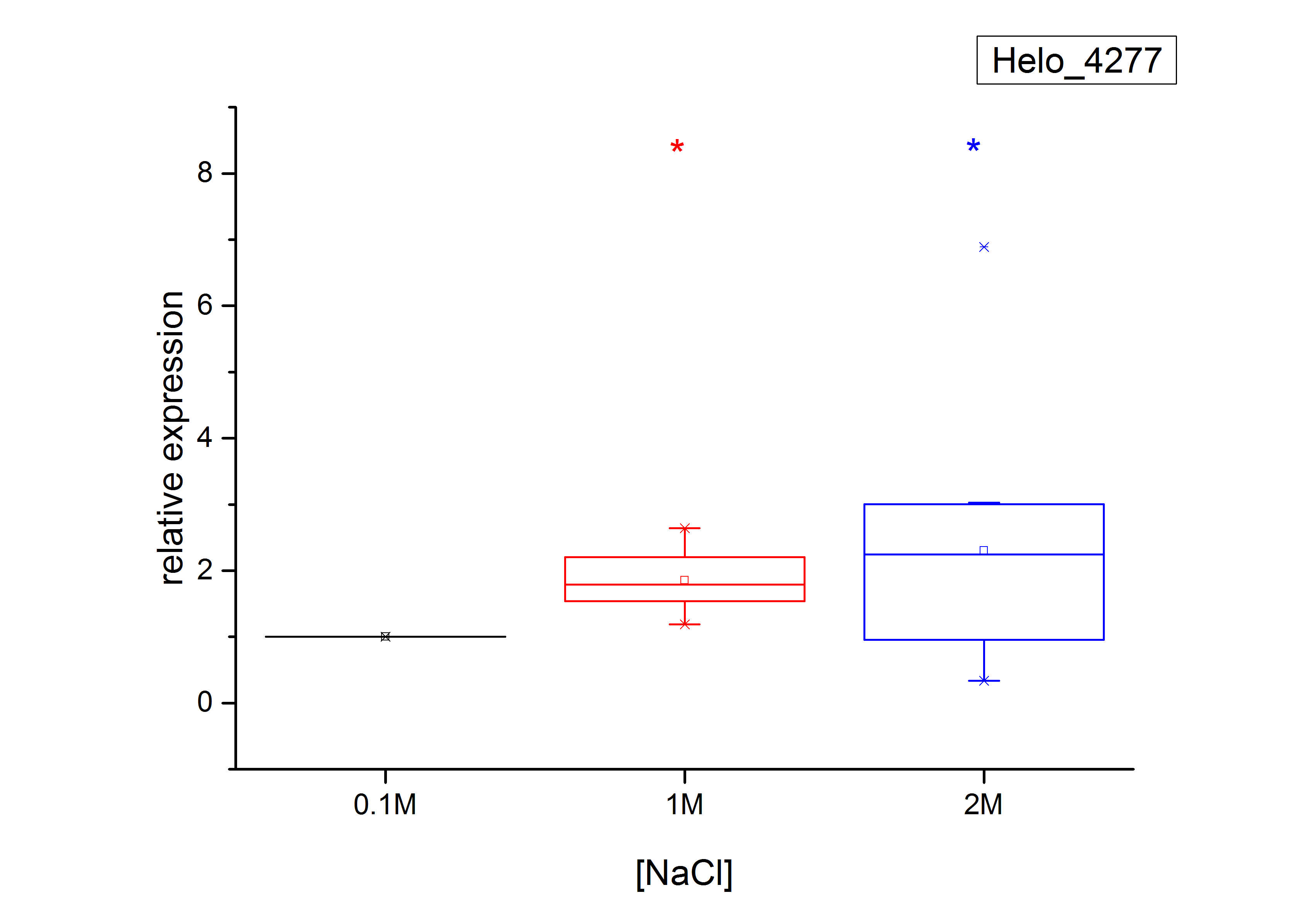


**Figure W. Validation of expression of the *teaD* gene by RT-qPCR.**

For details see Figure A.

**Table A. Primers used for qPCR analysis.**

| **primer** | **gene product** | **primer sequence** |
| --- | --- | --- |
| Helo_2186-1 | 6-phosphofructokinase | TGGCTCTTGACGGACAGAAC |
| Helo_2186-2 |  | GCATGAACTTCTCGCGGTTG |
| Helo_2298-1 | fumarate hydratase, class II | CGAGCAACATGAACGTCAATGA |
| Helo_2298-2 |  | ATCACGTCATTGCTCGACTGTC |
| Helo_2433-1 | phosphoenolpyruvate synthase | TGCCCGAGATTCCATTCAAGAT |
| Helo_2433-1 |  | ATTGATGATGAACTCCAGGCGA |
| Helo_2588-1 | L-2,4-diaminobutyric acid acetyltransferase | TCCACCATCTCGAGACCACTAT |
| Helo_2588-2 |  | GAAGTATTCGCGGCTGTTCAAC |
| Helo_2590-1 | L-ectoine synthase | ACTGCTCCTTCCACATCACC |
| Helo_2590-2 |  | TTGATGGGCCAGATCTTGCC |
| Helo_2675-1 | ornithine carbamoyltransferase | ACCAGGAGCATGTCGATCTTTG |
| Helo_2675-2 |  | TATGAATGAAGTCGACGCCCTT |
| Helo_2676-1 | arginine deiminase | AGCCGGGCGGTTTCCTCA |
| Helo_2676-2 |  | ATTGTTGCCGTCATCCCACTGCTC |
| Helo_3635-1 | 2-keto-3-deoxy- phosphogluconate aldolase | CGGTGCCGATTTCGTGGTGACA |
| Helo_3635-2 |  | GGAAGAACTTGAAGCGGCGGTAGC |
| Helo_3661-1 | diaminobutyrate--2-oxoglutarate transaminase | GCAGCGACGCCAATGAGACC |
| Helo_3661-2 |  | CGATGCCGGAGCCGTGATAGC |
| Helo_3664-1 | N-alpha-acetyl diaminobutyric acid deacetylase | GGCAACCACGGCGACGAGTA |
| Helo_3664-2 |  | CCCGGGAAGCTGCGATTGAGAT |
| Helo_3665-1 | ectoine hydrolase | GCGCTGCGGCTACCCTATCG |
| Helo_3665-2 |  | GCCCCAGTCCTCCACCCACAGA |
| Helo_3735-1 | oxaloacetate decarboxylase, alpha subunit | GACGTCCTGACCGAGATTCC |
| Helo_3735-2 |  | ACTTGTAGCGTTCACCCATCAT |
| Helo_3752-1 | glutamate synthase (NADPH), small subunit | CCGGTGGTGTTCGACAAGTA |
| Helo_3752-2 |  | CAGAACTCCACGCCCATCTC |
| Helo_3753-1 | glutamate synthase (NADPH), large subunit | TGGCCGTGCAGCAGAAGATGT |
| Helo_3753-2 |  | AGCCGCTGTTTGGTGGAGAGTA |
| Helo_3763-1 | malic enzyme | ACATGAAGGTGGCGGCAGTC |
| Helo_3763-2 |  | ATCGGCGTGGGAATAATGTAGTCA |
| Helo_4006-1 | quinoprotein glucose dehydrogenase | TCCCAAGCGCCAGGTGAT |
| Helo_4006-2 |  | AGGGCTTCATCTCGACCGCATAGG |
| Helo_4274-1 | TRAP transporter substrate-binding protein TeaA | TCGCTCAGGCTTTCAAGGGTTATG |
| Helo_4274-2 |  | AAGCCGGGCGACTGGTTCACG |
| Helo_2589-3 | diaminobutyrate--2-oxoglutarate transaminase | GCGCCACAATATCGTCTCCTTC |
| Helo_2589-4 |  | CTGCTGCCGAGGTAGCCATC |
| Helo_3049-3 | glutamate dehydrogenase (NAD+) | AAGCCGTCTGGACAAGGACAAG |
| Helo_3049-4 |  | TGAAGTGCCACACCGACAGG |
| Helo_3070-3 | isocitrate lyase | GCTGGGAGCTCAACCACCT |
| Helo_3070-4 |  | CACATGGGTTTCGAAGTTGTCG |
| Helo_3628-3 | phosphogluconate dehydratase | TCTCCCGCGACGTCATTG |
| Helo_3628-4 |  | GATACGCGCCTTCTCCTTGTTGG |
| Helo_3819-3 | alanine dehydrogenase | ATGAATATCGCGTTGCCCTGAC |
| Helo_3819-4 |  | TGGGGCTCCTTGACCTTGAG |
| Helo_4277-3 | regulatory protein TeaD | GGCGCCGAACTCTATATCCTCTG |
| Helo_4277-4 |  | GTCGTCCGGGATGTCCAACT |
