# Supplementary material for: Osmoregulation in the Halophilic Bacterium Halomonas elongata: A Case Study for Integrative Systems Biology
Source: PLoS One. 2017 Jan 12;12(1):e0168818. doi: 10.1371/journal.pone.0168818 (PMC5231179; doi:10.1371/journal.pone.0168818)
Supplement: S2 File — Tables B and C. (DOCX) [file pone.0168818.s002.docx]

**Table B. Differently expressed proteins at 0.1 M, 1 M, and 2 M (0.1%, 6% and 12%) sodium chloride grouped according to their function.** Major function categories are indicated by underline and indicated by a two- or three-letter tag. Minor function categories are without underline and repeat the tag of the major category. Regulation factors are shown for proteins from membrane (M) and the cytoplasmic (C) samples. Regulation factors are given for samples from 0.1 M NaCl compared to 1 M NaCl (1M NaCl) and samples form 1 M NaCl compared to 2 M NaCl (2 M NaCl). Regulation factors were determined as described in Materials and Methods. ND: not detected, NQ: not quantified. Superscripts (^A-Z^) following the gene code refer to protein complexes consisting of different subunits. Additional (non-quantified or unregulated) subunits are listed in S2 Table.

| **Gene** | **Protein name** | **Regulation factor** | | | |
| --- | --- | --- | --- | --- | --- |
|  |  | **M 1M NaCl** | **M 2M NaCl** | **C 1M NaCl** | **C 2M NaCl** |
| **CS compatible solutes** | | | | | |
| **CS** ectoine synthesis | | | | | |
| Helo_2588 | L-2,4-diaminobutyrate acetyltransferase  (EctA) | N.D. | N.D. | 5,94 | N.D. |
| Helo_2589 | diaminobutyrate--2-oxoglutarate transaminase (EctB) | N.D. | N.Q. | 13,45 | -1,17 |
| Helo_2590 | L-ectoine synthase (EctC) | N.D. | N.Q. | 2,71 | N.D. |
| **CS** ectoine degradation | | | | | |
| Helo_3661 | diaminobutyrate--2-oxoglutarate transaminase  (DoeD) | N.D. | N.D. | -4,01 | 1,96 |
| Helo_3664 | N-alpha-acetyl diaminobutyrate deacetylase (DoeB) | N.D. | N.D. | -3,11 | N.D. |
| Helo_3665 | ectoine hydrolase (DoeA) | N.Q. | N.D. | -6,14 | N.D. |
| **CS** betaine synthesis | | | | | |
| Helo_1859 | choline dehydrogenase BetA (BetA) | N.D. | N.Q. | 2,03 | 2,51 |
| Helo_1860 | betaine aldehyde dehydrogenase BetB (BetB) | N.D. | N.Q. | 2,20 | 2,39 |
| **CIM central intermediary metabolism** | | | | | |
| **CIM** pyruvate metabolism | | | | | |
| Helo_1685 | phosphoenolpyruvate carboxykinase (ATP) (PckA) | N.D. | N.Q. | -3,36 | 1,81 |
| Helo_2433 | phosphoenolpyruvate synthase (PpsA) | 2,54 | 1,53 | -1,08 | 1,20 |
| Helo_3010 | phosphoenolpyruvate carboxylase (Ppc) | 3,52 | 1,79 | 1,17 | 1,17 |
| Helo_3734^A^ | oxaloacetate decarboxylase beta subunit (OadB) | 1,80 | 1,30 | N.D. | N.D. |
| Helo_3735^A^ | oxaloacetate decarboxylase alpha subunit (OadA) | -1,31 | N.Q. | 2,02 | -1,02 |
| Helo_3763 | malic enzyme (NADP) (MaeB) | N.D. | N.Q. | 2,11 | -1,11 |
| **CIM** TCA cycle | | | | | |
| Helo_2298 | fumarate hydratase class II (FumC) | N.D. | N.D. | 2,21 | 1,06 |
| Helo_2437 | aconitate hydratase / 2-methylisocitrate dehydratase (AcnB) | 2,04 | 1,36 | 1,22 | 1,08 |
| **CIM** miscellaneous | | | | | |
| Helo_1220 | L-lactate dehydrogenase (cytochrome) (LldD) | -2,21 | N.Q. | 1,36 | -2,11 |
| Helo_1599 | ureidoglycolate hydrolase (AllA) | N.D. | N.D. | -2,04 | N.D. |
| Helo_2817 | acetaldehyde dehydrogenase (NAD) (AcoD) | N.Q. | N.Q. | -3,72 | 1,01 |
| Helo_3070 | isocitrate lyase (AceA) | N.D. | N.Q. | -4,26 | N.D. |
| Helo_3563 | acetyl-CoA synthetase (Acs) | 2,40 | N.Q. | 1,31 | 1,01 |
| Helo_3667 | glyoxylate/hydroxypyruvate reductase (GhrA) | N.D. | N.D. | -2,02 | N.D. |
| Helo_4242 | glyceraldehyde 3-phosphate dehydrogenase (GapA1) | 1,25 | -1,88 | 1,07 | -1,28 |
| **AA amino acid metabolism** | | | | | |
| **AA** glutamate family (glutamate, glutamine, proline, arginine) | | | | | |
| Helo_2743 | argininosuccinate synthase (ArgG) | 1,92 | 1,18 | 1,27 | -1,10 |
| Helo_1416 | arginine N-succinyltransferase subunit AruF (AruF) | N.D. | N.Q. | -2,03 | 1,54 |
| Helo_1417 | arginine N-succinyltransferase subunit AruG (AruG) | N.Q. | N.D. | -1,87 | N.D. |
| Helo_1418 | N-succinylglutamate 5-semialdehyde dehydrogenase (AruD) | N.D. | N.D. | -1,34 | 2,31 |
| Helo_1552 | succinylglutamate desuccinylase (AruE) | N.D. | N.D. | -1,95 | 1,88 |
| Helo_1802 | bifunctional proline dehydrogenase /  pyrroline-5-carboxylate dehydrogenase (PutA) | N.Q. | N.Q. | -2,29 | 2,01 |
| Helo_1972 | bifunctional ornithine acetyltransferase /  N-acetylglutamate synthase (ArgJ) | N.Q. | N.Q. | 2,07 | 1,21 |
| Helo_2675 | ornithine carbamoyltransferase (ArcB) | N.Q. | N.D. | -1,86 | -8,85 |
| Helo_2676 | arginine deiminase (ArcA) | N.D. | N.D. | -1,89 | -7,99 |
| Helo_4005 | arginine decarboxylase (SpeA) | N.D. | N.D. | -1,84 | -1,14 |
| Helo_3752^B^ | glutamate synthase (NADP) small subunit (GltD) | N.D. | N.Q. | 2,79 | 1,17 |
| Helo_3753^B^ | glutamate synthase (NADP) large subunit (GltB) | 2,37 | 1,67 | 2,25 | 1,19 |
| **AA** aspartate family (aspartate, asparagine, alanine, threonine, methionine, lysine) | | | | | |
| Helo_2252 | aspartate aminotransferase / aromatic amino acid aminotransferase (PhhC) | 2,49 | 1,09 | 1,28 | 1,05 |
| Helo_3136 | aspartate racemase (RacD) | N. D. | N.Q. | -2,10 | N.D. |
| Helo_3609 | alanine racemase (Alr) | N.D. | N.D. | -3,41 | 1,19 |
| Helo_3819 | alanine dehydrogenase (Ald) | N.D. | N.D. | -1,48 | 6,33 |
| Helo_4387 | probable methionine synthase YxjH, cobalamin-independent (YxjH) | N.Q. | N.Q. | -1,88 | -1,48 |
| Helo_1353 | cystathionine beta-lyase (PatB) | N.D. | N.D. | -2,36 | -1,36 |
| **AA** serine family (serine, glycine, cysteine) | | | | | |
| Helo_3446 | sarcosine oxidase alpha subunit | N.D. | N.D. | -3,92 | -1,63 |
| Helo_3449 | glycine hydroxymethyltransferase (GlyA2) | N.D. | N.Q. | -2,71 | N.D. |
| Helo_3598 | glycine hydroxymethyltransferase (GlyA1) | 2,41 | 1,36 | 1,40 | -1,15 |
| **AA** branched-chain amino acids (valine, leucine, isoleucine) | | | | | |
| Helo_1333^C^ | acetolactate synthase catalytic subunit (IlvI) | N.Q. | N.Q. | -1,98 | -1,04 |
| Helo_1334^C^ | acetolactate synthase regulatory subunit (IlvH) | -1,41 | N.Q. | -1,82 | N.D. |
| Helo_1337 | ketol-acid reductoisomerase (IlvC) | N.Q. | -1,32 | -4,27 | -1,39 |
| Helo_1522 | dihydroxy-acid dehydratase (IlvD2) | N.D. | N.Q. | 3,25 | 1,67 |
| Helo_1679 | dihydroxy-acid dehydratase (IlvD1) | N.Q. | 1,69 | 6,51 | -1,06 |
| Helo_2372^D^ | branched-chain alpha-keto acid dehydrogenase component E2 (BkdB) | N.Q. | N.Q. | -5,41 | 5,94 |
| Helo_1515 | methylmalonate-semialdehyde dehydrogenase (MmsA1) | N.D. | N.D. | -14,97 | 1,70 |
| Helo_2423 | isobutyryl-CoA dehydrogenase | N.Q. | N.Q. | -2,70 | 2,42 |
| Helo_2933 | probable enoyl-CoA hydratase | N.D. | N.D. | -2,98 | N.D. |
| Helo_2934^E^ | methylcrotonoyl-CoA carboxylase alpha subunit (MccA) | N.Q. | N.Q. | -3,03 | N.D. |
| Helo_2935 | hydroxymethylglutaryl-CoA lyase (HmgL1) | N.D. | N.Q. | -2,24 | 2,77 |
| **AA** aromatic amino acids (phenylalanine, tyrosine, tryptophan, histidine) | | | | | |
| Helo_2476 | urocanate hydratase (HutU) | N.D. | N.D. | 1,00 | 3,87 |
| Helo_4016 | imidazoleglycerol-phosphate dehydratase (HisB) | N.D. | N.D. | -2,07 | N.D. |
| Helo_2239^F^ | tryptophan synthase alpha subunit (TrpA2) | N.D. | N.Q. | 2,54 | N.D. |
| **NUM nucleotide metabolism** | | | | | |
| Helo_1531 | N5-carboxyaminoimidazole ribonucleotide mutase (PurE) | N.D. | N.D. | 2,47 | N.D. |
| Helo_1713 | 3',5'-cyclic adenosine monophosphate phosphodiesterase CpdA (CpdA) | N.D. | N.D. | -1,88 | N.D. |
| Helo_4434^G^ | ribonucleotide-diphosphate reductase beta subunit (NrdF) | N.D. | N.D. | 1,07 | -2,07 |
| Helo_3160 | thymidine phosphorylase (DeoA) | N.D. | N.D. | 1,86 | N.D. |
| **CHM carbohydrate metabolism** | | | | | |
| **CHM** Entner-Doudoroff pathway | | | | | |
| Helo_1553 | gluconolactonase (Gnl) | N.D. | N.D. | -3,81 | N.D. |
| Helo_3277^H^ | gluconate 2-dehydrogenase (acceptor) flavoprotein subunit | N.D. | N.D. | -3,85 | 2,40 |
| Helo_3628 | phosphogluconate dehydratase (Edd) | 2,22 | 1,88 | 1,71 | 1,17 |
| Helo_3635 | 2-keto-3-deoxy-phosphogluconate aldolase (Eda) | N.Q. | N.Q. | 1,89 | N.D. |
| Helo_4006 | quinoprotein glucose dehydrogenase (Gcd) | -1,92 | -1,48 | -1,14 | -1,05 |
| **CHM** miscellaneous | | | | | |
| Helo_1572 | probable glycosyltransferase, type 9 | -12,64 | N.Q. | N.D. | N.D. |
| Helo_1814 | glucan biosynthesis protein OpgD (OpgD) | N.D. | N.D. | -4,08 | -1,46 |
| Helo_1949 | phosphoheptose isomerase (GmhA) | N.D. | N.D. | -2,31 | N.D. |
| Helo_2397 | glucose-1-phosphate thymidylyltransferase (RmlA) | -1,26 | N.Q. | 1,40 | -2,18 |
| Helo_2709 | beta-hexosaminidase (NagZ) | N.D. | N.D. | -2,30 | -1,05 |
| Helo_2796 | L-arabinose 1-dehydrogenase (AraA) | N.D. | N.D. | -1,45 | 1,87 |
| Helo_2797 | L-arabonate dehydratase (araC2) | N.D. | N.D. | -2,07 | 1,03 |
| Helo_2800 | L-arabonate dehydratase (araC1) | N.Q. | N.D. | -6,13 | 1,40 |
| Helo_3145 | glucan biosynthesis protein OpgG (OpgG) | N.D. | N.D. | -2,45 | N.D. |
| Helo_3146 | glucan biosynthesis glucosyltransferase OpgH (OpgH) | -2,15 | -1,26 | N.D. | N.D. |
| Helo_3428 | lytic murein transglycosylase (Slt) | N.D. | N.Q. | -2,18 | 1,08 |
| Helo_3492 | ribitol 2-dehydrogenase (RbtD) | N.D. | N.Q. | 47,02 | N.D. |
| Helo_3596 | UDP-galactopyranose mutase (RfbD) | N.D. | N.D. | 2,39 | 1,13 |
| Helo_3698 | multiphosphoryl transfer protein, fructose-specific (FruB) | -2,38 | N.Q. | -1,02 | 2,27 |
| Helo_3829 | phosphoglucomutase / phosphomannomutase (AlgC) | 1,89 | 1,02 | 1,37 | -1,13 |
| Helo_4435 | D-fructose-6-phosphate amidotransferase (GlmS) | N.D. | N.Q. | -3,02 | -1,11 |
| **LIP lipid metabolism** | | | | | |
| **LIP** fatty acid metabolism | | | | | |
| Helo_1758^I^ | multifunctional fatty acid oxidation complex alpha subunit (FadB) | -2,47 | N.Q. | -2,20 | -1,45 |
| Helo_1759^I^ | multifunctional fatty acid oxidation complex beta subunit (FadA) | -1,92 | N.Q. | -1,99 | -1,32 |
| Helo_1858 | acyl-CoA dehydrogenase (FadE) | -2,06 | -2,25 | N.D. | N.D. |
| Helo_2118 | long-chain-fatty-acid--CoA ligase (FadD) | N.D. | N.D. | -1,45 | -1,83 |
| Helo_4131 | acetyl-CoA acetyltransferase (PhbA) | N.Q. | N.Q. | -2,80 | -1,88 |
| **LIP** misceallenous | | | | | |
| Helo_1108 | glycerophosphoryl diester phosphodiesterase (GlpQ) | N.D. | N.Q. | N.D. | 2,02 |
| Helo_2426 | glutaryl-CoA dehydrogenase (GcdH) | N.D. | N.D. | 2,64 | -1,08 |
| **COM coenzyme metabolism** | | | | | |
| **COM** biotin synthesis | | | | | |
| Helo_2169 | 8-amino-7-oxononanoate synthase (BioF) | N.D. | N.D. | 1,57 | 2,37 |
| Helo_2170 | biotin synthase (BioB) | N.D. | N.Q. | 1,84 | 2,64 |
| **COM** other coenzymes | | | | | |
| Helo_1204 | 5,10-methylenetetrahydrofolate reductase (MetF) | 2,24 | 1,91 | 1,84 | -1,09 |
| Helo_1831 | CobW family protein (CobW) | 2,18 | 1,48 | 1,91 | 1,97 |
| Helo_1832^J^ | cobaltochelatase subunit CobN (CobN) | -1,81 | N.Q. | 2,84 | 1,61 |
| Helo_1842 | cob(I)yrinic acid a,c-diamide adenosyltransferase (CobO) | N.D. | N.Q. | -2,01 | N.D. |
| Helo_2031 | GTP cyclohydrolase FolE2 (FolE2) | N.D. | N.D. | -1,47 | 2,11 |
| Helo_2550 | nicotinamide-nucleotide adenylyltransferase / ADP-ribose diphosphatase (NadM) | N.D. | N.D. | -1,94 | N.D. |
| Helo_2711 | dephospho-CoA kinase (CoaE) | N.D. | N.D. | -1,96 | N.D. |
| Helo_3148 | phosphomethylpyrimidine synthase (ThiC) | N.Q. | N.D. | -1,32 | 2,00 |
| Helo_3779 | porphobilinogen synthase (HemB) | N.D. | N.D. | 1,40 | -2,35 |
| Helo_4128 | 3-methyl-2-oxobutanoate hydroxymethyltransferase (PanB) | N.D. | N.D. | -1,85 | -1,23 |
| **EM energy metabolism** | | | | | |
| **EM** ATP synthase | | | | | |
| Helo_2667^K^ | V-type ATP synthase subunit A (AtvA) | N.Q. | N.Q. | -2,39 | N.D. |
| Helo_2673^K^ | V-type ATP synthase subunit B (AtvB) | -2,41 | N.Q. | N.D. | N.D. |
| Helo_4443^L^ | F0F1-type ATP synthase epsilon subunit (AtpC) | N.D. | 1,26 | 1,88 | N.D. |
| Helo_4445^L^ | F0F1-type ATP synthase gamma subunit (AtpG) | 1,09 | 1,21 | 1,82 | 1,18 |
| Helo_4448^L^ | F0F1-type ATP synthase subunit b (AtpF) | 1,60 | 1,16 | 3,40 | N.D. |
| **EM** NADH:quinone oxidoreductase | | | | | |
| Helo_2215^M^ | Na(+)-translocating NADH-quinone reductase subunit A (NqrA) | 1,31 | -1,14 | 2,07 | 1,20 |
| Helo_2216^M^ | Na(+)-translocating NADH-quinone reductase subunit B (NqrB) | 1,23 | -1,08 | 3,06 | 1,05 |
| Helo_2220^M^ | Na(+)-translocating NADH-quinone reductase subunit F (NqrF) | 1,38 | N.Q. | 2,85 | 1,11 |
| **ENZ other enzymes** | | | | | |
| **ENZ** proteolysis | | | | | |
| Helo_1358 | aminopeptidase PepA (PepA) | N.Q. | N.Q. | 2,14 | 1,55 |
| Helo_1872 | C-terminal processing peptidase (Prc) | 2,04 | N.Q. | 1,02 | -1,20 |
| Helo_2012 | protease HtpX (HtpX) | -4,52 | N.Q. | N.D. | N.D. |
| Helo_2024 | glutamate carboxypeptidase | N.D. | N.D. | 3,65 | -1,25 |
| Helo_2201 | D-alanyl-D-alanine carboxypeptidase DacA (DacA1) | -2,66 | N.Q. | -1,08 | -1,42 |
| Helo_2347 | ATP-dependent Clp protease ATP-binding subunit ClpX (ClpX) | -1,15 | 2,41 | 1,56 | 1,25 |
| Helo_2445 | peptidase U32 family protein | -1,19 | 2,04 | -1,01 | 1,74 |
| Helo_3026 | serine endoprotease DegP (DegP1) | N.Q. | N.Q. | -4,32 | -1,83 |
| Helo_3611 | peptidase M23 family protein | 1,20 | -2,58 | N.D. | -1,18 |
| Helo_3797 | peptidase M50 family protein | -2,19 | -1,13 | N.D. | N.D. |
| Helo_3870 | peptidase M16 family protein | N.Q. | N.D. | -3,34 | -1,65 |
| **ENZ** miscellaneous enzymes | | | | | |
| Helo_1054 | FAD-dependent oxidoreductase | N.D. | N.D. | -1,91 | N.D. |
| Helo_1093 | ferredoxin--NADP+ reductase (Fpr) | 2,27 | N.D. | 2,27 | 1,42 |
| Helo_1166 | HAD superfamily hydrolase | N.D. | N.D. | 2,45 | N.D. |
| Helo_1190 | oxidoreductase (homolog to zinc-containing alcohol dehydrogenase) | N.D. | N.D. | 2,04 | -1,30 |
| Helo_1209^N^ | GMC family oxidoreductase (homolog to lactose dehydrogenase flavoprotein subunit) | N.Q. | N.Q. | 3,07 | 1,17 |
| Helo_1210^N^ | homolog to lactose dehydrogenase small subunit | N.D. | N.D. | 3,21 | N.D. |
| Helo_1286 | probably oxidoreductase, molybdopterin-containing | N.Q. | N.Q. | 2,04 | 1,17 |
| Helo_1326 | FAD-dependent oxidoreductase | N.D. | N.Q. | -1,48 | -2,00 |
| Helo_1356 | probable oxidoreductase (short-chain dehydrogenase family) | N.D. | N.D. | 19,52 | 1,55 |
| Helo_1409 | peroxiredoxin | N.D. | N.D. | -1,86 | N.D. |
| Helo_1444 | beta-lactamase (BlaA) | N.D. | N.D. | -3,36 | N.D. |
| Helo_1498 | ArsH family oxidoreductase | N.D. | N.Q. | 5,30 | N.D. |
| Helo_1560 | alkaline phosphatase family protein | N.D. | N.Q. | 31,35 | 1,04 |
| Helo_1562 | dihydrodipicolinate synthetase domain protein | N.D. | N.D. | 3,34 | N.D. |
| Helo_1714^O^ | sulfate adenylyltransferase subunit 2 (cysD1) | 2,01 | 1,85 | 1,36 | 1,71 |
| Helo_1715^O^ | sulfate adenylyltransferase subunit 1 (cysN1) | 2,92 | 1,75 | 1,84 | 2,71 |
| Helo_1737 | homolog to phosphoadenosine phosphosulfate reductase | -1,56 | N.Q. | -2,04 | N.D. |
| Helo_1878 | 4-diphosphocytidyl-2-C-methyl-D-erythritol kinase (IspE) | N.D. | N.D. | -1,96 | N.D. |
| Helo_1897 | homolog to formate dehydrogenase alpha subunit | N.D. | N.Q. | -1,07 | 3,08 |
| Helo_1974 | N-carbamoylputrescine amidase (AguB) | N.D. | N.Q. | -2,38 | N.D. |
| Helo_2027 | homolog to gamma-glutamyltranspeptidase | N.D. | N.D. | 3,06 | N.D. |
| Helo_2143 | probable oxidoreductase (short-chain dehydrogenase family) | N.D. | N.D. | -1,93 | 1,50 |
| Helo_2144 | acyl-CoA dehydrogenase family protein | N.D. | N.D. | -2,38 | -1,20 |
| Helo_2148 | probable oxidoreductase (short-chain dehydrogenase family) | N.D. | N.D. | 1,75 | 1,89 |
| Helo_2290 | SGNH family hydrolase | -3,12 | N.Q. | N.D. | N.D. |
| Helo_2331 | FAD-dependent oxidoreductase | N.D. | N.D. | -2,10 | N.D. |
| Helo_2342 | S-ribosylhomocysteine lyase (LuxS) | N.D. | N.D. | 2,14 | N.D. |
| Helo_2387^P^ | sulfate adenylyltransferase subunit 2 (cysD2) | 2,00 | 1,40 | 1,39 | -1,25 |
| Helo_2389^P^ | sulfate adenylyltransferase subunit 1 (cysN2) | 2,23 | N.Q. | 1,21 | -1,38 |
| Helo_2499 | oxidoreductase (homolog to zinc-containing alcohol dehydrogenase) | N.Q. | N.Q. | 1,06 | -6,63 |
| Helo_2542 | 3-hydroxybutyrate dehydrogenase (Bdh) | N.D. | N.D. | -1,92 | 1,04 |
| Helo_2569 | probable oxidoreductase (short-chain dehydrogenase family) | N.D. | N.Q. | -2,47 | N.D. |
| Helo_2613 | NAD(P)H dehydrogenase (quinone) (WrbA) | N.D. | N.D. | -2,04 | N.D. |
| Helo_2805 | alpha-N-arabinofuranosidase | N.D. | N.D. | -9,68 | N.D. |
| Helo_2807 | aldehyde dehydrogenase | N.D. | N.D. | -4,21 | 1,76 |
| Helo_2853^Q^ | respiratory nitrate reductase beta subunit (NarH) | 3,64 | N.Q. | N.D. | -1,66 |
| Helo_2854^Q^ | respiratory nitrate reductase alpha subunit (NarG) | N.Q. | -3,18 | 1,76 | -1,59 |
| Helo_2886 | oxidoreductase (homolog to zinc-containing alcohol dehydrogenase) | N.D. | N.D. | -7,27 | 1,38 |
| Helo_3169 | GFO family oxidoreductase | N.Q. | 1,20 | 4,05 | -1,13 |
| Helo_3189 | N-ethylmaleimide reductase (NemA) | N.D. | N.D. | -2,64 | -1,35 |
| Helo_3204 | GFO family oxidoreductase | N.D. | N.Q. | 2,69 | 1,36 |
| Helo_3381 | gamma-glutamyltranspeptidase (Ggt) | N.D. | N.D. | 1,74 | -1,87 |
| Helo_3394 | poly-beta-hydroxyalkanoate polymerase (PhbC) | -2,57 | N.Q. | N.D. | N.D. |
| Helo_3422^R^ | NAD(P) transhydrogenase beta subunit (PntB) | 2,33 | 1,04 | 2,06 | N.D. |
| Helo_3423^R^ | NAD(P) transhydrogenase alpha subunit (PntA) | 2,34 | 1,09 | N.D. | -1,06 |
| Helo_3466 | formaldehyde dehydrogenase, glutathione-dependent (FrmA) | N.D. | N.Q. | 2,42 | 5,35 |
| Helo_3489 | S-adenosylmethionine decarboxylase (SpeD) | N.D. | N.D. | -1,86 | N.D. |
| Helo_3534 | 2,4-dienoyl-CoA reductase (FadH) | N.D. | N.D. | -2,95 | -1,56 |
| Helo_3658 | HAD superfamily hydrolase | N.D. | N.Q. | -1,82 | N.D. |
| Helo_3761 | peptidoglycan transglycosylase / transpeptidase | -2,27 | -1,45 | -1,04 | N.D. |
| Helo_3881 | GNAT family acetyltransferase | N.D. | N.D. | -3,21 | N.D. |
| Helo_4098 | probable oxidoreductase | -2,26 | N.Q. | N.D. | N.D. |
| Helo_4112 | anhydro-N-acetylmuramic acid kinase | N.D. | N.D. | -2,06 | N.D. |
| Helo_4301 | peroxiredoxin | N.Q. | N.Q. | -1,98 | N.D. |
| Helo_4329 | alkyl hydroperoxide reductase subunit F (AhpF) | N.D. | N.Q. | -1,47 | 4,28 |
| Helo_4414 | rubredoxin--NAD reductase | N.Q. | N.Q. | N.D. | 1,83 |
| **TP Transport** | | | | | |
| **TP** ABC-type transport system | | | | | |
| Helo_1551 | ABC-type transport system ATP-binding protein | -2,90 | 1,48 | 1,31 | 2,53 |
| Helo_1794 | ABC-type transport system periplasmic substrate-binding protein | N.D. | N.Q. | 3,23 | N.D. |
| Helo_1862 | ABC-type transport system periplasmic substrate-binding protein | N.D. | N.D. | 4,87 | 1,75 |
| Helo_1929 | ABC-type transport system ATP-binding protein (probable substrate phospholipid) (MlaF) | -1,92 | -1,58 | -1,06 | -1,77 |
| Helo_2016 | ABC-type transport system ATP-binding protein | N.Q. | 1,88 | N.D. | N.D. |
| Helo_2321 | ABC-type transport system ATP-binding protein | -2,29 | N.Q. | N.D. | N.D. |
| Helo_2362 | ABC-type transport system periplasmic substrate-binding protein | N.D. | N.D. | 3,18 | N.D. |
| Helo_2366 | ABC-type transport system periplasmic substrate-binding protein (probably substrate osmolyte) (OsmX) | N.D. | N.D. | N.D. | -2,04 |
| Helo_2458 | ABC-type transport system ATP-binding/permease protein (CydD) | -2,63 | N.Q. | N.D. | N.D. |
| Helo_2585 | ABC-type transport system periplasmic substrate-binding protein | N.D. | N.D. | 2,18 | 1,33 |
| Helo_2696^S^ | ABC-type transport system periplasmic substrate-binding protein | N.D. | N.D. | -2,33 | 1,39 |
| Helo_2699^S^ | ABC-type transport system ATP-binding protein | -3,18 | N.Q. | N.D. | N.D. |
| Helo_2833 | ABC-type transport system ATP-binding/permease protein | 4,03 | N.Q. | N.D. | N.D. |
| Helo_2985 | ABC-type transport system periplasmic substrate-binding protein | N.D. | N.Q. | 1,90 | -1,36 |
| Helo_3091^T^ | ABC-type transport system ATP-binding protein | N.Q. | -11,43 | N.D. | -6,56 |
| Helo_3094^T^ | ABC-type transport system periplasmic substrate-binding protein | N.D. | N.D. | 78,46 | -5,93 |
| Helo_3340 | ABC-type transport system permease protein | -1,89 | 1,09 | N.D. | N.D. |
| Helo_3356^U^ | ABC-type transport system ATP-binding protein (probable substrate glycine betaine) (GbuA) | N.Q. | 2,08 | 1,96 | 2,33 |
| Helo_3357^U^ | ABC-type transport system permease protein (probable substrate glycine betaine) (GbuB) | 1,09 | 2,17 | N.D. | N.D. |
| Helo_3385 | ABC-type transport system ATP-binding protein (probable substrate sulfate/thiosulfate) (CysA) | N.Q. | 1,37 | N.D. | -2,82 |
| Helo_3600 | ABC-type transport system permease protein | -4,39 | -1,56 | N.D. | N.D. |
| Helo_3674^V^ | ABC-type transport system periplasmic substrate-binding protein | N.D. | N.Q. | 3,79 | -2,55 |
| Helo_3676^V^ | ABC-type transport system permease protein | 3,01 | -1,87 | N.D. | N.D. |
| Helo_3678^V^ | ABC-type transport system ATP-binding protein | 2,39 | -1,88 | 5,41 | -1,82 |
| Helo_3679^V^ | ABC-type transport system ATP-binding protein | 1,64 | -1,35 | 8,62 | -2,08 |
| Helo_3845^W^ | ABC-type transport system periplasmic substrate-binding protein | N.D. | N.D. | -2,18 | 2,46 |
| Helo_3846^W^ | ABC-type transport system periplasmic substrate-binding protein | N.D. | N.D. | -2,10 | N.D. |
| Helo_3849^W^ | ABC-type transport system ATP-binding protein | N.Q. | 2,48 | N.D. | N.D. |
| Helo_3970 | ABC-type transport system periplasmic substrate-binding protein | N.D. | N.D. | 1,94 | -1,54 |
| Helo_4001 | ABC-type transport system periplasmic substrate-binding protein (probable substrate iron) (FutA) | N.D. | N.D. | 2,40 | N.D. |
| Helo_4010^X^ | ABC-type transport system periplasmic substrate-binding protein | N.D. | N.Q. | 2,93 | N.D. |
| Helo_4011^X^ | ABC-type transport system ATP-binding protein | -1,23 | 8,18 | N.D. | N.D. |
| Helo_4154 | ABC-type transport system periplasmic substrate-binding protein | N.D. | N.D. | 1,87 | -1,06 |
| Helo_4316 | ABC-type transport system ATP-binding protein | -2,17 | -1,52 | N.D. | -1,20 |
| Helo_4388^U^ | ABC-type transport system periplasmic substrate-binding protein (probable substrate glycine betaine) (GbuC) | N.D. | N.Q. | 47,77 | 1,71 |
| Helo_4458 | ABC-type transport system periplasmic substrate-binding protein (probable substrate glutamine) (GlnH) | N.D. | N.D. | 2,59 | -1,75 |
| **TP** TRAP transporter | | | | | |
| Helo_1094 | TRAP transporter substrate-binding protein | N.D. | N.D. | -3,03 | -1,63 |
| Helo_1206 | TRAP transporter substrate-binding protein | N.D. | N.D. | -3,41 | N.D. |
| Helo_1527 | TRAP transporter substrate-binding protein | N.D. | N.D. | 2,33 | 1,10 |
| Helo_1776 | TRAP transporter substrate-binding protein | N.D. | N.D. | 2,35 | 1,01 |
| Helo_3703 | TRAP transporter, 4TM/12TM fusion protein | -2,04 | -1,05 | N.D. | N.D. |
| Helo_3707^Y^ | TRAP transporter small transmembrane protein | -1,98 | N.Q. | N.D. | N.D. |
| Helo_3708^Y^ | TRAP transporter substrate-binding protein | N.D. | N.D. | -1,20 | -2,09 |
| Helo_3998 | TRAP transporter large transmembrane protein | -2,24 | N.Q. | N.D. | N.D. |
| Helo_4274 | TRAP transporter substrate-binding protein (substrate ectoine) (TeaA) | N.D. | N.Q. | 3,81 | -1,44 |
| Helo_4277 | UspA domain transporter regulator TeaD (TeaD) | N.D. | N.Q. | 3,22 | N.D. |
| Helo_4425 | TRAP transporter substrate-binding protein | N.D. | N.D. | -1,32 | -1,86 |
| **TP** BCC family transporter | | | | | |
| Helo_3358 | BCC family transporter BetH (substrate glycine-betaine) (BetH) | 1,26 | 2,13 | N.D. | 2,60 |
| Helo_4292 | BCC family transporter | 1,26 | 2,71 | N.D. | N.D. |
| **TP** sodium/proton antiporter family protein | | | | | |
| Helo_1427 | sodium/proton antiporter (NhaD1) | 2,04 | N.Q. | N.D. | N.D. |
| Helo_3371 | sodium/proton antiporter family protein | N.Q. | 3,09 | N.D. | N.D. |
| **TP** potassium transport protein | | | | | |
| Helo_3903 | potassium-efflux system protein KefB (KefB) | -2,36 | N.Q. | N.D. | N.D. |
| **TP** mechanosensitive channel | | | | | |
| Helo_2045 | mechanosensitive ion channel MscK (MscK) | -2,26 | -1,23 | N.D. | N.D. |
| Helo_3378 | mechanosensitive ion channel MscS (MscS1) | -1,48 | -2,18 | -1,02 | N.D. |
| **TP** outer membrane beta-barrel proteins | | | | | |
| Helo_1704 | outer membrane porin | 2,69 | 1,32 | 3,29 | 1,67 |
| Helo_1822 | TonB-dependent receptor BtuB (probable substrate cobalamin) | 2,15 | 1,70 | N.D. | 2,47 |
| Helo_2827 | TonB-dependent receptor | 16,88 | N.Q. | N.D. | N.D. |
| Helo_3304 | TonB-dependent receptor IutA (probable substrate ferric enterobactin) (IutA) | 7,07 | N.Q. | N.D. | N.D. |
| Helo_3316 | TonB-dependent receptor | 4,06 | N.Q. | N.D. | N.D. |
| Helo_3326 | TonB-dependent receptor | 18,22 | N.Q. | N.D. | N.D. |
| Helo_3673 | sucrose porin (ScrY) | 335,34 | N.Q. | N.D. | N.D. |
| Helo_3682 | maltoporin (LamB1) | 1,91 | N.Q. | 1,65 | N.D. |
| Helo_3850 | outer membrane porin | 3,24 | 7,55 | N.D. | N.D. |
| **TP** other transporters | | | | | |
| Helo_1043 | TTT family transporter substrate-binding protein | N.D. | N.D. | 2,57 | 1,01 |
| Helo_1073 | major facilitator superfamily transporter | 3,60 | N.Q. | N.D. | N.D. |
| Helo_1111 | SDF family transporter | -1,82 | 2,58 | N.D. | N.D. |
| Helo_1307 | major facilitator superfamily transporter | 2,40 | 3,98 | N.D. | N.D. |
| Helo_1595 | NCS1 family transporter | -2,80 | N.Q. | N.D. | N.D. |
| Helo_1625 | TTT family transporter substrate-binding protein | N.D. | N.D. | -3,71 | N.D. |
| Helo_1801 | SSS family transporter (probable substrate proline) (OpuE) | -1,83 | N.Q. | N.D. | N.D. |
| Helo_2046 | HlyD family secretion protein | N.Q. | 3,00 | N.D. | N.D. |
| Helo_2047 | HlyB family toxin translocation ATP-binding protein | 2,01 | 2,04 | N.D. | N.D. |
| Helo_2391 | polysaccharide export protein KpsD (KpsD) | 2,68 | -1,39 | -1,22 | -1,24 |
| Helo_2528 | SAF family transporter (probable substrate alanine) | 1,10 | 1,47 | N.D. | 1,82 |
| Helo_2563 | ExbB family protein TolQ (TolQ) | -1,91 | -1,14 | 1,12 | N.D. |
| Helo_2565 | TonB-like protein TolA (TolA) | -2,51 | N.Q. | N.D. | N.D. |
| Helo_2566 | protein TolB (TolB) | N.Q. | N.Q. | -1,92 | -1,13 |
| Helo_3056 | outer-membrane lipoprotein carrier protein LolA (LolA) | N.D. | N.Q. | -1,99 | N.D. |
| Helo_3126 | HAE1 family transporter | -2,86 | N.Q. | N.D. | N.D. |
| Helo_3127 | RND family transporter MFP component | -2,40 | -1,09 | N.D. | N.D. |
| Helo_3085 | SSS family transporter | -1,26 | 1,89 | N.D. | 2,87 |
| Helo_3268 | SDF family transporter (probable substrate serine/threonine) (SstT) | -2,35 | N.Q. | N.D. | N.D. |
| Helo_3548 | SSS family transporter | N.Q. | -1,83 | 1,64 | N.D. |
| Helo_3899 | RND family transporter MFP component | 4,65 | -1,29 | N.D. | N.D. |
| Helo_3900 | HAE1 family transporter | 3,75 | -1,15 | N.D. | N.D. |
| **SIG signal transduction** | | | | | |
| Helo_1575 | response regulator / HTH domain protein | -2,36 | N.Q. | -6,32 | N.D. |
| Helo_1576 | sensor histidine kinase | -7,55 | 1,41 | N.D. | N.D. |
| Helo_1618 | sensor kinase protein | -1,87 | -1,37 | N.D. | N.D. |
| Helo_1706 | PAS domain protein / diguanylate cyclase domain protein | -2,11 | N.Q. | N.D. | N.D. |
| Helo_2122 | response regulator domain protein / PP2C domain protein | N.D. | N.D. | -2,64 | -1,34 |
| Helo_2249 | PAS domain / diguanylate cyclase domain / diguanylate phosphodiesterase domain protein | -2,05 | N.Q. | N.D. | N.D. |
| Helo_2855 | sensor histidine kinase NarX (NarX) | -2,95 | N.Q. | N.D. | N.D. |
| Helo_3015 | sensor histidine kinase / response regulator | -2,23 | -1,28 | -1,03 | N.D. |
| Helo_3246 | sensor histidine kinase | -1,81 | N.Q. | N.D. | N.D. |
| Helo_3689 | sensor histidine kinase BaeS (BaeS) | -2,89 | N.Q. | N.D. | N.D. |
| **REG gene regulation** | | | | | |
| Helo_1156 | XylR family transcriptional regulator | N.D. | N.D. | 1,80 | 1,44 |
| Helo_1394 | IscR family transcription regulator IscR (IscR) | N.D. | N.D. | 1,83 | N.D. |
| Helo_1499 | ArsR family transcription regulator | N.D. | N.D. | 6,89 | N.D. |
| Helo_1652 | PmpR family transcription regulator | N.D. | N.D. | -2,00 | -1,38 |
| Helo_1661 | response regulator/HTH domain protein | N.D. | N.D | 5,53 | N.D |
| Helo_1736 | LysR family transcription regulator CysB (CysB) | 3,44 | N.Q. | 1,46 | -1,22 |
| Helo_2383 | GntR family transcription regulator CsiR (CsiR) | N.D. | N.D. | -1,84 | N.D. |
| Helo_2559 | PmpR family transcription regulator | N.D. | N.D. | N.D. | 2,07 |
| Helo_3464 | CdhR family transcription regulator | N.Q. | N.Q. | -4,39 | N.D. |
| Helo_3503 | PhaR family transcription regulator | N.D. | N.Q. | 3,94 | N.D. |
| Helo_3638 | HexR family transcription regulator HexR (HexR) | 3,56 | N.Q. | N.D. | -1,87 |
| Helo_3663 | AsnC/Lrp family transcriptional regulator DoeX (DoeX) | N.D. | N.D. | -3,90 | N.D. |
| Helo_3686 | MalT family transcription regulator MalT (MalT) | N.D. | -1,55 | 2,00 | N.D. |
| Helo_3740 | LysR family transcription regulator | N.D. | N.D. | -4,36 | N.D. |
| Helo_3901 | TetR family transcription regulator | N.Q. | N.D. | 2,02 | N.D. |
| Helo_4419 | regulatory protein PhoU (PhoU) | N.D. | N.D. | -2,47 | -1,91 |
| **TC transcription** | | | | | |
| Helo_1229 | DNA-directed RNA polymerase beta subunit (RpoB) | 1,40 | 1,86 | 1,25 | 1,30 |
| Helo_1277 | RNA polymerase-associated protein RapA (RapA) | 1,95 | 1,50 | 1,16 | 1,38 |
| Helo_2164 | probable RNA polymerase sigma factor | -2,33 | -1,35 | N.D. | N.D. |
| **TL translation** | | | | | |
| **TL** ribosomal protein | | | | | |
| Helo_1231 | 30S ribosomal protein S12 | N.Q. | 1,69 | 2,15 | 1,51 |
| Helo_1232 | 30S ribosomal protein S7 | N.Q. | -1,21 | 2,02 | N.D. |
| Helo_1235 | 30S ribosomal protein S10 | 1,74 | N.Q. | 2,29 | N.D. |
| Helo_1236 | 50S ribosomal protein L3 | 1,49 | N.Q. | 1,95 | N.D. |
| Helo_1240 | 30S ribosomal protein S19 | N.D. | N.Q. | 2,36 | N.D. |
| Helo_1242 | 30S ribosomal protein S3 | 1,96 | 1,99 | 1,97 | 1,57 |
| Helo_1249 | 30S ribosomal protein S14 | N.Q. | N.Q. | 2,92 | N.D. |
| Helo_1250 | 30S ribosomal protein S8 | N.D. | N.D. | 1,85 | N.D. |
| Helo_1253 | 30S ribosomal protein S5 | 1,07 | 1,21 | 2,08 | N.D. |
| Helo_1258 | 30S ribosomal protein S13 | N.Q. | N.Q. | 1,85 | 1,60 |
| Helo_1259 | 30S ribosomal protein S11 | 1,17 | N.Q. | 2,04 | N.D. |
| Helo_1260 | 30S ribosomal protein S4 | 1,50 | 1,18 | 2,09 | 1,59 |
| Helo_1314 | 30S ribosomal protein S20 | N.D. | N.Q. | 2,24 | N.D. |
| Helo_1942 | 30S ribosomal protein S9 | 1,42 | 1,61 | 2,50 | -1,35 |
| Helo_3525 | 30S ribosomal protein S18 | 1,14 | 1,19 | 2,30 | N.D. |
| Helo_3526 | 30S ribosomal protein S6 | N.Q. | N.Q. | 2,05 | N.D. |
| Helo_3804 | 30S ribosomal protein S2 | 1,82 | 2,06 | 1,45 | 1,36 |
| Helo_3863 | 30S ribosomal protein S16 | N.D. | N.Q. | 2,23 | N.D. |
| Helo_4134 | 30S ribosomal protein S15 | N.D. | N.Q. | 2,26 | N.D. |
| **TL** miscellaneous | | | | | |
| Helo_1233 | translation elongation factor G (FusA) | 1,45 | 1,93 | 1,08 | 1,44 |
| Helo_1234 | translation elongation factor Tu (Tuf) | 1,42 | 2,18 | 1,13 | 1,22 |
| Helo_3022 | translation elongation factor LepA (LepA) | 1,94 | 1,13 | 1,17 | -1,07 |
| Helo_3743 | alanine--tRNA ligase (AlaS) | 1,94 | 1,43 | 1,18 | 1,12 |
| Helo_3843 | lysine--tRNA ligase (LysS) | 2,25 | N.Q. | 1,84 | 1,18 |
| **RRR replication, repair, recombination** | | | | | |
| Helo_1721 | DNA topoisomerase IV subunit A (ParC) | N.Q. | N.Q. | 1,88 | -3,03 |
| Helo_2546 | UvrABC system protein B (UvrB) | 1,36 | 1,14 | -1,89 | -1,06 |
| Helo_2561 | Holliday junction ATP-dependent DNA helicase RuvA (RuvA) | N.D. | N.D. | -1,83 | N.D. |
| Helo_3745 | protein RecA (RecA) | 1,23 | 1,40 | 2,45 | 1,13 |
| Helo_3823 | formamidopyrimidine-DNA glycosylase (MutM) | N.D. | N.Q. | 1,15 | 2,32 |
| Helo_3859 | site-specific tyrosine recombinase XerD (XerD) | N.D. | N.D. | -2,41 | N.D. |
| Helo_3993 | DNA recombination protein RmuC (RmuC) | -2,01 | N.Q. | N.D. | N.D. |
| Helo_4161 | DNA repair protein RecN (RecN) | N.Q. | N.Q. | -1,11 | 2,70 |
| Helo_4426 | DNA-dependent helicase II (UvrD) | 2,09 | 1,37 | 1,43 | 1,42 |
| **MIS miscellaneous other proteins** | | | | | |
| Helo_1006 | NAD-dependent epimerase/dehydratase | N.D. | N.D. | -1,14 | -1,80 |
| Helo_1078 | probable usher pathway chaperone | N.D. | N.D. | -1,92 | N.D. |
| Helo_1134 | CoA transferase family protein | N.Q. | N.D. | 2,10 | -1,63 |
| Helo_1332 | molecular chaperone ClpB (ClpB) | N.Q. | N.Q. | 1,21 | -2,90 |
| Helo_1347 | LOG family protein | N.D. | N.D. | -1,99 | N.D. |
| Helo_1348 | DUF501 family protein | N.D. | N.D. | -2,02 | N.D. |
| Helo_1367 | NAD-dependent protein deacylase (NpdA1) | N.Q. | N.Q. | -2,20 | N.D. |
| Helo_1383 | ribosome maturation GTPase Der (Der) | -2,48 | -1,05 | -1,70 | -1,13 |
| Helo_1384 | outer membrane protein assembly factor BamB (BamB) | 1,58 | -2,48 | 1,16 | 1,36 |
| Helo_1388 | RodZ family protein | -2,14 | N.Q. | 2,17 | 1,14 |
| Helo_1400^Z^ | protein translocase subunit SecD (SecD) | -1,25 | -1,05 | 1,96 | 1,07 |
| Helo_1401^Z^ | protein translocase subunit YajC (YajC) | N.Q. | -1,05 | 2,09 | N.D. |
| Helo_1439 | DUF899 family protein | N.D. | N.D. | -5,75 | N.D. |
| Helo_1445 | YhfK family protein | N.D. | N.Q. | 1,80 | N.D. |
| Helo_1510 | DUF2252 family protein | -1,99 | N.Q. | N.D. | N.D. |
| Helo_1539 | probable oxidoreductase (iron-containing alcohol dehydrogenase family) | N.D. | N.D. | -3,06 | N.D. |
| Helo_1600 | YlbA family protein | N.D. | N.D. | -1,80 | -1,21 |
| Helo_1609 | DUF192 family protein | N.D. | N.D. | -3,08 | N.D. |
| Helo_1632 | 23S rRNA (guanine(745)-N(1))-methyltransferase RlmA (RlmA) | N.D. | N.D. | -3,06 | N.D. |
| Helo_1651 | DUF306 domain protein | -2,35 | -2,51 | -2,07 | N.D. |
| Helo_1673 | helicase domain protein | N.D. | N.Q. | N.D. | -1,88 |
| Helo_1684 | molecular chaperone HslO (HslO) | N.D. | N.D. | 2,69 | 1,43 |
| Helo_1733 | DUF541 family protein | N.D. | N.Q. | -6,10 | -3,85 |
| Helo_1784 | iron-sulfur cluster assembly complex subunit SufB (SufB) | N.D. | N.Q. | 1,10 | 2,19 |
| Helo_1785 | iron-sulfur cluster assembly complex subunit SufC (SufC) | 1,30 | 1,98 | 1,13 | N.D. |
| Helo_1820 | homolog to phosphoglycerate mutase | N.D. | N.D. | -1,92 | N.D. |
| Helo_1879 | outer membrane lipoprotein receptor LolB (LolB) | N.D. | -1,86 | N.D. | N.D. |
| Helo_1891 | GlnA/PuuA domain protein | N.Q. | -2,21 | 1,15 | -1,87 |
| Helo_1924 | lipopolysaccharide export system ATP-binding protein LptB (LptB) | -1,82 | N.Q. | -1,04 | N.D. |
| Helo_1932 | MlaC family protein (MlaC) | -1,53 | -1,99 | -1,11 | N.D. |
| Helo_1934 | BolA family protein | N.D. | N.Q. | 1,84 | N.D. |
| Helo_1950 | UPF0102 family protein | N.D. | N.D. | -2,31 | N.D. |
| Helo_1951 | LpoA family protein | 1,39 | -2,66 | 1,38 | 1,21 |
| Helo_1975 | homolog to agmatine deiminase | N.D. | N.D. | -3,53 | N.D. |
| Helo_2005 | TatD family protein | N.D. | N.D. | N.D. | 1,96 |
| Helo_2030 | DNA-binding protein Zur (Zur) | -2,87 | N.Q. | -3,06 | 4,39 |
| Helo_2123 | STAS domain protein | N.D. | N.D. | -2,05 | N.D. |
| Helo_2163 | RskA family protein | -2,03 | -1,36 | N.D. | N.D. |
| Helo_2182 | thiol:disulfide interchange protein DsbD (DsbD) | -2,54 | -1,31 | N.D. | N.D. |
| Helo_2185 | protein phosphatase domain protein / protein kinase domain protein | -2,00 | 1,16 | N.D. | N.D. |
| Helo_2190 | YabI family protein | -2,97 | N.Q. | N.D. | N.D. |
| Helo_2207 | DUF3141 family protein | -1,78 | -2,11 | N.D. | N.D. |
| Helo_2287 | iron-sulfur cluster assembly protein ApbC (ApbC) | 1,29 | N.D. | -2,34 | -1,19 |
| Helo_2294 | DnaA regulatory inactivator Hda | N.D. | N.D. | -2,07 | N.D. |
| Helo_2336 | DjlA domain protein | N.D. | N.Q. | -2,07 | N.D. |
| Helo_2350 | PpiC-type peptidyl-prolyl cis-trans isomerase family protein | -1,82 | N.Q. | 2,00 | 1,30 |
| Helo_2376 | DUF3584 family protein | 1,41 | 1,91 | 1,27 | 1,63 |
| Helo_2400 | phytanoyl-CoA dioxygenase domain protein | -1,97 | N.Q. | N.D. | N.D. |
| Helo_2421 | acyl-CoA synthetase family protein | N.D. | N.D. | 1,35 | 2,15 |
| Helo_2439 | tRNA-(ms[2]io[6]A)-hydroxylase (MiaE) | N.Q. | N.Q. | 2,34 | N.D. |
| Helo_2540 | glutaredoxin (GrxA) | N.D. | N.D. | 1,87 | N.D. |
| Helo_2607 | ferredoxin domain protein / 4Fe-4S domain protein | 1,79 | 2,63 | 1,35 | 1,37 |
| Helo_2608 | DUF934 family protein | N.D. | N.D. | 1,91 | N.D. |
| Helo_2628 | hydrolase domain protein | N.D. | N.Q. | 2,74 | 1,69 |
| Helo_2683 | THI5 family protein | N.D. | N.D. | -1,85 | N.D. |
| Helo_2700 | DUF2219 family protein | -2,80 | -1,66 | N.D. | N.D. |
| Helo_2704 | RarD family protein | -3,08 | N.Q. | N.D. | N.D. |
| Helo_2846 | non-ribosomal peptide synthetase AT domain protein | N.Q. | N.Q. | 2,17 | N.D. |
| Helo_2874 | molecular chaperone GroES (GroES) | N.D. | N.Q. | -2,22 | N.D. |
| Helo_2875 | molecular chaperone GroEL (GroEL) | -2,29 | N.Q. | -2,35 | -1,06 |
| Helo_2876 | tRNA (cytidine/uridine-2'-O-)-methyltransferase TrmJ (TrmJ2) | N.D. | N.Q. | 2,90 | N.D. |
| Helo_2910 | T6SS cluster protein VasK | 1,38 | -1,22 | 2,80 | -1,30 |
| Helo_2916 | T6SS cluster protein VasF | 1,46 | N.Q. | 1,28 | -2,99 |
| Helo_2917 | T6SS cluster protein VasE | 5,05 | -1,85 | N.D. | N.D. |
| Helo_2954 | ydfM family protein | -2,26 | -1,73 | N.D. | N.D. |
| Helo_3021 | signal peptidase I (LepB) | -2,61 | -1,59 | 1,66 | -1,25 |
| Helo_3033 | ATP-dependent RNA helicase RhlB | 2,26 | 1,48 | 1,62 | 1,31 |
| Helo_3046 | ribosomal RNA large subunit methyltransferase RlmKL | N.D. | N.D. | -2,15 | -1,75 |
| Helo_3057 | DNA translocase FtsK (FtsK) | -2,11 | N.Q. | -1,22 | -1,92 |
| Helo_3090 | DUF1479 family protein | N.D. | N.Q. | N.D. | -8,57 |
| Helo_3121 | CBS domain protein | N.D. | N.D. | -4,20 | N.D. |
| Helo_3129 | alpha/beta hydrolase fold protein | N.D. | N.D. | -2,82 | N.D. |
| Helo_3205 | iolE family protein | N.D. | N.D. | 2,70 | 1,32 |
| Helo_3242 | glutamate synthase domain protein | -1,29 | -2,42 | N.D. | N.D. |
| Helo_3276 | LmbE family protein | 2,72 | 1,26 | N.D. | N.D. |
| Helo_3296 | DUF2075 domain protein | N.Q. | 1,91 | N.D. | 2,52 |
| Helo_3317 | DUF1007 family protein | N.D. | N.D. | -1,99 | N.D. |
| Helo_3322 | DUF1486 family protein | N.D. | N.D. | -2,18 | N.D. |
| Helo_3323 | YwnB family protein | N.D. | N.D. | -2,10 | N.D. |
| Helo_3389 | (dimethylallyl)adenosine tRNA methylthiotransferase MiaB (MiaB) | N.D. | 2,33 | N.D. | 1,64 |
| Helo_3398 | 50S ribosomal protein L3 glutamine methyltransferase (PrmB) | N.D. | N.Q. | 1,22 | -3,81 |
| Helo_3405 | NAD-dependent epimerase/dehydratase | N.D. | N.D. | 1,88 | -1,06 |
| Helo_3426 | esterase domain protein | N.D. | N.D. | 4,62 | N.D. |
| Helo_3442 | DUF294/CBS domain protein | -3,17 | N.Q. | -2,26 | -1,42 |
| Helo_3484 | peptidyl-prolyl cis-trans isomerase SurA (SurA) | N.Q. | -3,23 | 1,02 | N.D. |
| Helo_3506 | MltB family protein | N.Q. | -3,93 | -1,87 | -2,71 |
| Helo_3528 | ribonuclease R (Rnr) | 2,06 | N.Q. | 1,12 | 1,06 |
| Helo_3533 | PrkA family protein | N.Q. | N.Q. | 1,25 | -2,53 |
| Helo_3582 | UPF0118 family protein | -4,20 | -1,59 | N.D. | N.D. |
| Helo_3671 | DUF3108 family protein | N.D. | N.D. | -1,84 | N.D. |
| Helo_3677 | alpha amylase family protein | N.D. | N.Q. | 4,75 | -1,85 |
| Helo_3681 | MalM family protein | N.Q. | -2,51 | N.D. | N.D. |
| Helo_3687 | alpha amylase family protein | N.D. | N.D. | 1,26 | -2,07 |
| Helo_3702 | UspA domain protein | N.D. | N.Q. | -2,49 | N.D. |
| Helo_3714 | tRNA 5-methylaminomethyl-2-thiouridine biosynthesis bifunctional protein MnmC (MnmC) | N.D. | N.D. | -1,84 | N.D. |
| Helo_3777 | twin arginine translocation system protein TatC (TatC) | N.Q. | -1,86 | N.D. | N.D. |
| Helo_3851 | FadL family protein | -1,38 | -2,56 | N.D. | N.D. |
| Helo_3858 | DsbC family protein | N.D. | N.D. | -2,07 | -1,10 |
| Helo_3872 | cell division topological specificity factor MinE (MinE) | N.D. | N.Q. | -1,92 | N.D. |
| Helo_3873 | septum site-determining protein MinD (MinD) | -2,42 | N.Q. | -1,81 | -1,24 |
| Helo_3874 | septum site-determining protein MinC (MinC) | N.D. | N.Q. | 1,88 | N.D. |
| Helo_3923 | YhbW family protein | N.D. | N.D. | 2,06 | -1,56 |
| Helo_4017 | DUF3749 family protein | N.D. | N.Q. | -2,05 | N.D. |
| Helo_4018 | AsmA family protein | -2,42 | N.Q. | N.D. | N.D. |
| Helo_4107 | NlpE domain protein | 1,59 | -2,63 | 1,65 | N.D. |
| Helo_4153 | DUF2218 family protein | N.D. | N.D. | 1,87 | N.D. |
| Helo_4163 | outer membrane protein assembly factor BamE (BamE) | 1,55 | N.Q. | 1,79 | N.D. |
| Helo_4167 | UPF0150 family protein | N.D. | N.Q. | 2,29 | N.D. |
| Helo_4252 | tetratricopeptide repeat protein | N.Q. | -1,08 | 2,09 | -1,03 |
| Helo_4437 | ApbE family protein | -2,20 | -1,54 | -1,12 | N.D. |
| Helo_4454 | ribosomal RNA small subunit methyltransferase RsmG (RsmG) | N.D. | N.Q. | -2,42 | N.D. |
| **HY (conserved) hypothetical proteins** | | | | | |
| Helo_1091 | conserved hypothetical protein | -4,55 | -1,30 | N.D. | N.D. |
| Helo_1340 | conserved hypothetical protein | N.Q. | -1,82 | N.D. | N.D. |
| Helo_1467 | conserved hypothetical protein | 1,17 | 1,80 | N.D. | N.D. |
| Helo_1812 | conserved hypothetical protein | N.D. | N.D. | -2,26 | -2,51 |
| Helo_2050 | conserved hypothetical protein | N.D. | N.Q. | -1,61 | -2,03 |
| Helo_2051 | conserved hypothetical protein | N.D. | N.Q. | 2,07 | -2,15 |
| Helo_2192 | conserved hypothetical protein | -2,83 | N.Q. | N.D. | N.D. |
| Helo_2211 | conserved hypothetical protein | 3,42 | N.Q. | N.D. | N.D. |
| Helo_2242 | conserved hypothetical protein | -2,45 | -1,28 | N.D. | N.D. |
| Helo_2286 | conserved hypothetical protein | N.Q. | N.Q. | 2,96 | -1,00 |
| Helo_2297 | conserved hypothetical protein | -6,76 | N.Q. | N.D. | N.D. |
| Helo_2303 | conserved hypothetical protein | N.D. | N.Q. | 1,81 | 1,45 |
| Helo_2390 | conserved hypothetical protein | 2,59 | -1,35 | 1,32 | N.D. |
| Helo_2403 | conserved hypothetical protein | N.Q. | N.Q. | 2,27 | N.D. |
| Helo_2418 | conserved hypothetical protein | -1,67 | -2,12 | N.D. | N.D. |
| Helo_2419 | conserved hypothetical protein | -1,90 | -2,58 | N.D. | N.D. |
| Helo_2440 | conserved hypothetical protein | -1,12 | -1,30 | -2,36 | -1,45 |
| Helo_2861 | conserved hypothetical protein | N.D. | N.Q. | 2,29 | N.D. |
| Helo_2905 | conserved hypothetical protein | -1,77 | N.Q. | 1,90 | -1,87 |
| Helo_3123 | conserved hypothetical protein | N.D. | N.D. | -5,26 | N.D. |
| Helo_3125 | conserved hypothetical protein | -1,43 | -8,78 | N.D. | N.D. |
| Helo_3243 | conserved hypothetical protein | N.D. | N.Q. | 11,00 | N.D. |
| Helo_3303 | conserved hypothetical protein | ND. | N.Q. | -1,83 | -1,67 |
| Helo_3386 | conserved hypothetical protein | -1,29 | -1,82 | N.D. | N.D. |
| Helo_3395 | conserved hypothetical protein | -4,62 | N.Q. | N.D. | N.D. |
| Helo_3505 | conserved hypothetical protein | N.D. | N.D. | -2,74 | N.D. |
| Helo_3549 | conserved hypothetical protein | -1,91 | -1,75 | N.D. | N.D. |
| Helo_3599 | conserved hypothetical protein | N.Q. | N.Q. | -1,84 | -1,09 |
| Helo_3693 | conserved hypothetical protein | N.D. | N.D. | -3,96 | N.D. |
| Helo_3842 | conserved hypothetical protein | N.D. | N.D. | -2,31 | N.D. |
| Helo_3884 | conserved hypothetical protein | N.Q. | -3,03 | 1,06 | N.D. |
| Helo_3909 | conserved hypothetical protein | N.D. | N.D. | -1,35 | -1,83 |
| Helo_3913 | conserved hypothetical protein | -1,86 | -1,63 | N.D. | N.D. |
| Helo_4399 | conserved hypothetical protein | N.Q. | N.Q. | 1,98 | -1,01 |
| Helo_4400 | conserved hypothetical protein | 3,77 | 1,52 | 2,87 | -1,01 |

**Table C. Proteins mentioned in the manuscript that are not quantified or not differentially expressed according to LC-MS/MS analysis**. Major and minor function categories are indicated as described in Table S1. Regulation factors are shown for proteins from membrane (M) and the cytoplasmic (C) samples. Regulation factors are given for samples from 0.1 M NaCl compared to 1 M NaCl (1 M NaCl) and samples form 1 M NaCl compared to 2 M NaCl (2 M NaCl) (for further details see Table S1). ND: not detected, NQ: not quantified. Proteins with lettered superscripts following the gene code refer to protein complexes for which other subunits have been differentially expressed (see Table S1). For proteins with numbered superscripts (^1-4^), none of the subunits were differentially expressed.

| **Gene** | **Protein name** | ***Regulation factor** | | | |
| --- | --- | --- | --- | --- | --- |
|  |  | **M 1M NaCl** | **M 2M NaCl** | **C 1M NaCl** | **C 2M NaCl** |
| **CIM central intermediary metabolism** | | | | | |
| **CIM** pyruvate metabolism | | | | | |
| Helo_3736^A^ | oxaloacetate decarboxylase gamma subunit (OadC) | N.D. | N.D. | N.D. | N.D. |
| Helo_3817 | malic enzyme (NAD) (MaeA) | N.D. | N.Q. | -1,17 | 1,05 |
| Helo_4243 | pyruvate kinase (PykA1) | 1,23 | N.D. | -102. | -1,17 |
| **CIM** TCA cycle | | | | | |
| Helo_2547 | fumarate hydratase class I (FumA) | N.Q. | N.Q. | -1,10 | 1,00 |
| **AA amino acid metabolism** | | | | | |
| **AA** glutamate family (glutamate, glutamine, proline, arginine) | | | | | |
| Helo_1126 | glutamine synthetase (GlnA) | N.D. | N.Q. | 1,14 | 1,19 |
| Helo_1537 | GlnA domain protein | N.D. | N.D. | N.D. | N.D. |
| Helo_2746 | ornithine carbamoyltransferase (ArgF) | N.D. | N.D. | 1,01 | 1,09 |
| Helo_2764 | arginine—pyruvate aminotransferase (AruH) | N.D. | N.D. | N.D. | N.D. |
| Helo_3049 | glutamate dehydrogenase (NAD) (Gdh) | -1,76 | 1,24 | -1,79 | -1,04 |
| **AA** aspartate family (aspartate, asparagine, alanine, threonine, methionine, lysine) | | | | | |
| Helo_2013 | alanine aminotransferase (AlaA) | N.Q. | N.Q. | -1,04 | -1,09 |
| Helo_2235 | aspartate-semialdehyde dehydrogenase (Asd) | N.D. | N.Q. | 1,61 | 1,23 |
| Helo_3742 | aspartate kinase (LysC) | 1,54 | 1,48 | 1,74 | 1,21 |
| Helo_4120 | aspartate aminotransferase (AspC) | 1,62 | N.Q. | 1,19 | 1,05 |
| **AA** branched-chain amino acids (valine, leucine, isoleucine) | | | | | |
| Helo_1133 | hydroxymethylglutaryl-CoA lyase (HmgL2) | N.Q. | N.D. | -1,67 | N.D. |
| Helo_1292 | methylmalonate-semialdehyde dehydrogenase (MmsA2) | N.D. | N.D. | N.D. | N.D. |
| Helo_2370^D^ | branched-chain alpha-keto acid dehydrogenase component E1 alpha subunit (BkdA1) | N.D. | N.D. | N.D. | N.D. |
| Helo_2371^D^ | branched-chain alpha-keto acid dehydrogenase component E1 beta subunit (BkdA2) | N.D. | N.Q. | N.D. | N.D. |
| Helo_2932^E^ | methylcrotonoyl-CoA carboxylase beta subunit (MccB) | N.D. | N.Q. | N.D. | N.D. |
| Helo_4230 | methylmalonate-semialdehyde dehydrogenase (MmsA3) | N.D. | N.D. | N.D. | N.D. |
| **AA** aromatic amino acids (phenylalanine, tyrosine, tryptophan, histidine) | | | | | |
| Helo_2238^F^ | tryptophan synthase beta subunit (TrpB2) | N.D. | N.Q. | -1,15 | -1,24 |
| Helo_4326^1^ | tryptophan synthase beta subunit (TrpB1) | N.D. | N.D. | N.D. | N.D. |
| Helo_4327^1^ | tryptophan synthase alpha subunit (TrpA1) | N.D. | N.Q. | N.D. | N.D. |
| **NUM nucleotide metabolism** | | | | | |
| Helo_3889^2^ | ribonucleotide-diphosphate reductase alpha subunit (NrdA) | 1,53 | N.Q. | -1,02 | -1,03 |
| Helo_3890^2^ | ribonucleotide-diphosphate reductase beta subunit (NrdB) | N.Q. | N.Q. | 1,13 | 1,30 |
| Helo_4433^G^ | ribonucleotide-diphosphate reductase alpha subunit (NrdE) | N.Q. | N.Q. | -1,03 | -1,64 |
| **CHM carbohydrate metabolism** | | | | | |
| **CHM** Entner-Doudoroff pathway | | | | | |
| Helo_1782 | Gluconokinase (GntK) | N.D. | N.D. | N.D. | N.D. |
| Helo_2186 | 6-phosphofructokinase (PfkA) | N.Q. | N.Q. | 1,16 | -1,07 |
| Helo_3278^H^ | gluconate 2-dehydrogenase (acceptor) cytochrome subunit | N.D. | N.D. | N.D. | N.D. |
| Helo_3279^H^ | gluconate 2-dehydrogenase (acceptor) subunit 3 | N.D. | N.D. | N.D. | N.D. |
| Helo_3636 | 6-phosphogluconolactonase (Pgl) | N.D. | N.D. | 1,40 | N.D. |
| Helo_3637 | glucose-6-phosphate 1-dehydrogenase (Zwf) | N.Q. | N.Q. | 1,41 | -1,26 |
| **CHM** pentose phosphate pathway | | | | | |
| Helo_3118 | 6-phosphogluconate dehydrogenase (decarboxylating) | N.D. | N.D. | 1,34 | -1,69 |
| **COM coenzyme metabolism** | | | | | |
| **COM** biotin synthesis | | | | | |
| Helo_2155 | adenosylmethionine-8-amino-7-oxononanoate aminotransferase (BioA) | N.D. | N.D. | N.D. | -1,79 |
| Helo_2166 | dithiobiotin synthetase (BioD) | N.D. | N.Q. | -1,07 | N.D. |
| Helo_2167 | malonyl-CoA O-methyltransferase BioC (BioC) | N.D. | N.Q. | N.D. | N.D. |
| Helo_2168 | pimelyl-[acyl-carrier protein] methyl ester esterase (BioH) | N.Q. | N.D. | N.D. | N.D. |
| **COM** other coenzymes | | | | | |
| Helo_1829^J^ | Probable cobaltochelatase subunit ChlD (ChlD) | N.Q. | 1,66 | -1,06 | 1,33 |
| **EM energy metabolism** | | | | | |
| **EM** ATP synthase | | | | | |
| Helo_2668^K^ | V-type ATP synthase subunit X (AtvX) | N.D. | N.Q. | N.D. | N.D. |
| Helo_2669^K^ | V-type ATP synthase subunit E (AtvE) | N.Q. | N.Q. | N.D. | N.D. |
| Helo_2670^K^ | V-type ATP synthase subunit K (AtvK) | N.Q. | N.Q. | N.D. | N.D. |
| Helo_2671^K^ | V-type ATP synthase subunit I (AtvI) | N.Q. | N.Q. | N.D. | N.D. |
| Helo_2672^K^ | V-type ATP synthase subunit D (AtvD) | N.D. | N.Q. | N.D. | N.D. |
| Helo_4444^L^ | F0F1-type ATP synthase beta subunit (AtpD) | 1,41 | 1,45 | 1,69 | 1,24 |
| Helo_4446^L^ | F0F1-type ATP synthase alpha subunit (AtpA) | 1,34 | 1,33 | 1,68 | 1,17 |
| Helo_4447^L^ | F0F1-type ATP synthase delta subunit (AtpH) | N.Q. | 1,26 | 1,74 | N.D. |
| Helo_4449^L^ | F0F1-type ATP synthase subunit c (AtpE) | N.Q. | N.Q. | N.D. | N.D. |
| Helo_4450^L^ | F0F1-type ATP synthase subunit b (AtpB) | N.D. | N.Q. | N.D. | N.D. |
| **EM** NADH:quinone oxidoreductase | | | | | |
| Helo_2217^M^ | Na(+)-translocating NADH-quinone reductase subunit C (NqrC) | 1,33 | -1,05 | N.D. | 1,11 |
| Helo_2218^M^ | Na(+)-translocating NADH-quinone reductase subunit D (NqrD) | 1,42 | N.Q. | N.D. | N.D. |
| Helo_2219^M^ | Na(+)-translocating NADH-quinone reductase subunit E (NqrE) | N.D. | N.D. | N.D. | N.D. |
| **TP Transport** | | | | | |
| **TP** TRAP transporter | | | | | |
| Helo_4275 | TRAP transporter small transmembrane protein (substrate ectoine) (TeaB) | N.D. | N.Q. | N.D. | N.D. |
| Helo_4276 | TRAP transporter large transmembrane protein (substrate ectoine) (TeaC) | N.Q. | N.Q. | N.D. | N.D. |
| **TP** BCC family transporter | | | | | |
| Helo_1580 | BCCT family transporter BetG (substrate glycine-betaine) (BetG) | N.D. | N.Q. | N.D. | N.D. |
| **TP** MRP-type sodium/hydrogen antiporter | | | | | |
| Helo_3507^3^ | Mrp-type sodium/proton antiporter system subunit G (mrpG1) | N.Q. | N.Q. | N.D. | N.D. |
| Helo_3508^3^ | Mrp-type sodium/proton antiporter system subunit F (mrpF1) | N.D. | N.D. | N.D. | N.D. |
| Helo_3509^3^ | Mrp-type sodium/proton antiporter system subunit E (mrpE1) | N.D. | N.D. | N.D. | N.D. |
| Helo_3510^3^ | Mrp-type sodium/proton antiporter system subunit D (mrpD1) | N.Q. | N.Q. | N.D. | N.D. |
| Helo_3511^3^ | Mrp-type sodium/proton antiporter system subunit C (mrpC1) | N.D. | N.D. | N.D. | N.D. |
| Helo_3512^3^ | Mrp-type sodium/proton antiporter system subunit B (mrpB1) | N.D. | N.Q. | N.D. | N.D. |
| Helo_3513^3^ | Mrp-type sodium/proton antiporter system subunit A (mrpA1) | N.Q. | N.D. | N.D. | N.D. |
| Helo_3514^4^ | Mrp-type sodium/proton antiporter system subunit AB (mrpAB2) | N.Q. | 1,23 | N.D. | N.D. |
| Helo_3515^4^ | Mrp-type sodium/proton antiporter system subunit C (mrpC2) | N.D. | N.D. | N.D. | N.D. |
| Helo_3516^4^ | Mrp-type sodium/proton antiporter system subunit D (mrpD2) | 1,22 | 1,07 | N.D. | N.D. |
| Helo_3517^4^ | Mrp-type sodium/proton antiporter system subunit E (mrpE2) | N.Q. | N.Q. | N.D. | N.D. |
| Helo_3518^4^ | Mrp-type sodium/proton antiporter system subunit F (mrpF2) | N.D. | N.D. | N.D. | N.D. |
| Helo_3519^4^ | Mrp-type sodium/proton antiporter system subunit G (mrpG2) | N.D. | N.D. | N.D. | N.D. |
| **TP** sodium/proton antiporter family protein | | | | | |
| Helo_1086 | sodium/proton antiporter family protein | -1,44 | N.Q. | N.D. | N.D. |
| Helo_1557 | malate/lactate antiporter (MleN) | N.Q. | N.Q. | N.D. | N.D. |
| Helo_1901 | sodium/proton antiporter family protein | N.Q. | N.Q. | N.D. | N.D. |
| Helo_2323 | sodium/proton antiporter (NhaD2) | N.Q. | N.Q. | N.D. | N.D. |
| Helo_2474 | sodium/proton antiporter family protein | N.Q. | N.Q. | N.D. | N.D. |
| Helo_2682 | potassium/proton antiporter (NhaP1) | N.Q. | N.Q. | N.D. | N.D. |
| Helo_3921 | sodium/proton antiporter family protein | N.D. | N.D. | N.D. | N.D. |
| **TP** potassium transport protein | | | | | |
| Helo_1371 | trk system potassium uptake protein TrkH (TrkH) | N.D. | N.Q. | N.D. | N.D. |
| Helo_1372 | trk system peripheral membrane protein TrkA (TrkA) | N.Q. | 1,39 | N.D. | N.D. |
| Helo_1450 | trk system potassium uptake protein TrkI (TrkI) | N.D. | N.Q. | N.D. | N.D. |
| **TP** mechanosensitive channel | | | | | |
| Helo_3171 | mechanosensitive ion channel MscS (MscS3) | -1,41 | -1,11 | N.D. | N.D. |
| Helo_4248 | mechanosensitive ion channel MscS (MscS2) | -1,36 | 1,33 | 1,38 | 1,76 |
| **TP** other transporters | | | | | |
| Helo_2217 | sodium-translocating NADH-quinone reductase subunit C (NqrC) | 1,33 | -1,05 | N.D. | 1,11 |
| Helo_2218 | sodium-translocating NADH-quinone reductase subunit D (NqrD) | 1,42 | N.Q. | N.D. | N.D. |
| Helo_2219 | sodium-translocating NADH-quinone reductase subunit E (NqrE) | N.D. | N.D. | N.D. | N.D. |
